# The Taxonomic Significance of Species That Have Only Been Observed Once: The Genus *Gymnodinium* (Dinoflagellata) as an Example

**DOI:** 10.1371/journal.pone.0044015

**Published:** 2012-08-30

**Authors:** Anne E. Thessen, David J. Patterson, Shauna A. Murray

**Affiliations:** 1 Center for Library and Informatics, Marine Biological Laboratory, Woods Hole, Massachusetts, United States of America; 2 School of Biotechnology and Biomolecular Sciences, University of New South Wales, Sydney, Australia; 3 Sydney Institute of Marine Sciences, Mosman, Australia; Université Paris Sud, France

## Abstract

Taxonomists have been tasked with cataloguing and quantifying the Earth’s biodiversity. Their progress is measured in code-compliant species descriptions that include text, images, type material and molecular sequences. It is from this material that other researchers are to identify individuals of the same species in future observations. It has been estimated that 13% to 22% (depending on taxonomic group) of described species have only ever been observed once. Species that have only been observed at the time and place of their original description are referred to as oncers. Oncers are important to our current understanding of biodiversity. They may be validly described species that are members of a rare biosphere, or they may indicate endemism, or that these species are limited to very constrained niches. Alternatively, they may reflect that taxonomic practices are too poor to allow the organism to be re-identified or that the descriptions are unknown to other researchers. If the latter are true, our current tally of species will not be an accurate indication of what we know. In order to investigate this phenomenon and its potential causes, we examined the microbial eukaryote genus *Gymnodinium*. This genus contains 268 extant species, 103 (38%) of which have not been observed since their original description. We report traits of the original descriptions and interpret them in respect to the status of the species. We conclude that the majority of oncers were poorly described and their identity is ambiguous. As a result, we argue that the genus *Gymnodinium* contains only 234 identifiable species. Species that have been observed multiple times tend to have longer descriptions, written in English. The styles of individual authors have a major effect, with a few authors describing a disproportionate number of oncers. The information about the taxonomy of *Gymnodinium* that is available via the internet is incomplete, and reliance on it will not give access to all necessary knowledge. Six new names are presented – *Gymnodinium campbelli* for the homonymous name *Gymnodinium translucens* Campbell 1973, *Gymnodinium antarcticum* for the homonymous name *Gymnodinium frigidum* Balech 1965, *Gymnodinium manchuriensis* for the homonymous name *Gymnodinium autumnale* Skvortzov 1968, *Gymnodinium christenum* for the homonymous name *Gymnodinium irregulare* Christen 1959, *Gymnodinium conkufferi* for the homonymous name *Gymnodinium irregulare* Conrad & Kufferath 1954 and *Gymnodinium chinensis* for the homonymous name *Gymnodinium frigidum* Skvortzov 1968.

## Introduction

It is estimated that there are 1.9 million described living species [1], less than one fifth of this number of described extinct species [2], and a debatable number of species left to be described but most estimates of the number of living species are in the region of 10 million [3]. These estimates are directly or indirectly based on the current inventory of species, but that inventory is uncertain given that not all species have been reliably described [4]. Of particular concern are species that are known from a single report. Such reports may not be of species previously unknown to science, but may be of damaged or teratological specimens, stages in the life history, or extremely variant forms of known species. The treatment of these descriptions as being of valid taxa would lead to the overestimation of known biodiversity.

The term ‘singleton’ has been used for taxa known from a single specimen in a sampling event, uniques being represented by more individuals but only in a single sample [5]. These terms are used both in the context of sampling and taxonomy. We introduce the term 'oncers' as a term limited to taxonomy, to refer to those species that have been described from a single collection event (whether one or multiple cells were observed), and for which no new data has been added at any time by subsequent studies. As many as 30% of species may fall into this category [5]. Oncers might reflect rare species [6], species with very limited geographical distributions, or species in tightly defined niches. Alternatively, oncers may be poor descriptions that unjustifiably add to our tally of species. We analyze the dinoflagellate genus *Gymnodinium* Stein 1879 [7] with the aim of quantifying the number of oncers and better understanding their nature.

Our observations not only bear on issues relating to the nature of the species and their descriptions, but on the online digital resources upon which we increasingly depend [8]. Within the sciences, taxonomy is especially reliant on nomenclatural and taxonomic acts that are located in literature published at any time in the last 250 years. Major digitization efforts are underway, such as the Biodiversity Heritage Library (BHL) which seeks to digitize biodiversity literature and will make more of the taxonomic and nomenclatural oinformation available. While new technologies bring advantages [9], [10], any research that relies on digital resources is vulnerable to the quantity and quality of digitized materials and to the application of copyright restrictions [11].

## Materials and Methods

Names of *Gymnodinium* species were collected from AlgaeBase (www.algaebase.org), Index Nominum Algarum (http://ucjeps.berkeley.edu/INA.html), the Global Names Index (http://gni.globalnames.org/) and Google searches of the internet that would access dedicated online resources such as dinoflaj (dinoflaj.smu.ca) and CEDiT (http://www.dinophyta.org/) and from recent reviews [7], [12]–[14]. A literature search was conducted for the original description using each name. If a name was found not to be code compliant, erroneously formed, or a nomen nudum, it was not considered. Each item was reviewed for information such as the number of words in the description, where the described material was collected, how often the taxon was observed and in how many collections, the language in which the description was written, the number of cells observed, the number of images available, how many other taxa were compared to the new species, information on type materials, and whether uninterpreted records (such as photographs) were included. All non-conflicting proposed synonymies were accepted.

In addition to the analysis of the literature we evaluated (July 2010) BHL (http://www.biodiversitylibrary.org), GBIF (http://www.gbif.org), GenBank (http://www.ncbi.nlm.nih.gov/genbank/), ISI/Web of Knowledge (http://www.webofknowledge.com) and Google Scholar (using the species name in quotes to obtain exact matches). The results are shown in Appendix S1. We included results for junior synonyms and misspelled names (Appendix S2). A name is considered misspelled if it deviates from the spelling in the original description and is not a code-compliant amendation.

A species was determined to be a oncer if all of the following criteria were met.

1. The literature and internet search failed to provide any evidence for observations of the species other than those in the original description.
2. Observations of organisms used for the original description were based on a single sample. If a species was observed on more than one occasion or in more than one place, but reported in a single publication, it was not treated as a oncer.
3. No type culture or laboratory strain is available. If a researcher can view the species alive at any time in the laboratory it is not considered a oncer even if no field observations have been recorded.

The quality, quantity and nature of the description were not used to define oncers. The availability of sequences, drawings and photographs taken during the original description do not prevent a species from being a oncer.

Throughout the following section we use the following terms as defined here:

1. *observed* – the species was actually seen
2. *reported* – the species is mentioned, but no new observations were made
3. *described* – refers to the original description only

## Results and Discussion

### Assessment of Species

Below is an alphabetical list of all *Gymnodinium* species found that satisfy the taxonomic criteria given in the Methods section. All names are accompanied by a brief description of their taxonomic history. All species that were determined to be oncers are labeled with an asterisk.

1. ***Gymnodinium absumens*** Schiller 1957* – This species was described by Schiller from several individuals collected in Lake Neusiedl, a freshwater lake in Central Europe [15]. He included five drawings of this species and a 152 word description in German that gave quantitative cell size measurements. It has not been observed since.
2. ***Gymnodinium achromaticum*** **Lebour 1917**– This species was described by Lebour based on a single cell found in the estuarine waters of Plymouth Sound, England [16]. She drew two images of the cell, ventral and side view. No quantitative measurements are available in her 40 word description in English. This species was referred to in several publications, but was not seen again until 1936 off the coast of Massachusetts by Lackey [17]. It was seen again in 1938 in brackish waters in Belgium [18]. Conrad and Kufferath [18] provided no new images nor morphological features, but provided some details of the environment in which the cell was found. The earliest quantitative measurements appeared in Kofoid & Swezy [19] who presumably calculated them from the original Lebour drawings, considering there is no evidence of new observations. In 1925, Lebour republished her description of *G. achromaticum* with the Kofoid and Swezy [19] measurements despite not having observed the species again [20]. Schiller published a German account of the species without new observations [21]. It was not until the 1960’s that *G. achromaticum* was again seen in Plymouth Sound [22]. Margalef reported seeing *G. achromaticum* in the NW Mediterranean [23]. In 1982, Dodge published a short account of *G. achromaticum* with a new image, presumably redrawn from Lebour [24]. The species was reported from the Aegean Sea in 2007 [25]. Two observations have been reported to GBIF. There are three unique drawings available depicting this species and no photographs.
3. ***Gymnodinium achroum*** Schiller 1957* - This species was described by Schiller from a few individuals collected in the freshwater Lake Neusiedl [15]. He included two drawings of this species, cell size measurements and a 165 word description in German. It has not been observed since.
4. ***Gymnodinium acutiusculum*** Okolodkov 1997* – This species was described by Okolodkov based on a single individual collected in the Greenland Sea [26]. There is one drawing in his 268 word, English description and no photographs. Cell measurements and some habitat information were given. This species has been observed once and no additional information can be found.
5. ***Gymnodinium adriaticum*** **(Schmarda) Kofoid & Swezy 1921**– This species was initially described as *Peridinium adriaticum* by Schmarda [27] who included 12 drawings and a 126 word description in German. Many individuals were found in salt pools near Trieste, Italy. He observed the species on two occasions, once in Trieste and again in Venice. It has not been observed since its discovery, despite being reported in the literature. Diesing transferred this species to *Heteraulacus* [28] and later to *Heteroaulax* [29]. Kofoid & Swezy finally placed it within *Gymnodinium* [19]. Very little information is available on *G. adriaticum*. *Peridinium adriaticum* Schmarda 1846 should not be confused with the homonym *Peridinium adriaticum* Broch 1910 which has been renamed *P. brochi* [19].
6. ***Gymnodinium aequatoriale*** **Hasle 1960**– This species was described by Hasle from hundreds of individuals collected from the equatorial Pacific Ocean [30]. She included five drawings, cell measurements and a 228 word, English and Latin description. It has one observation in GBIF.
7. ***Gymnodinium aeruginosum*** **Stein 1883**– This species was described by Stein using samples from an Austrian pond [31]. He gave no explicit text description, but does include four figures and descriptive figure captions in German. Stein did not include quantitative measurements from direct observations, but those can be found in later publications [31]–[36]. This species has been reported numerous times since its first description and seems to have a cosmopolitan distribution in freshwater ponds, bogs and rivers from oligo- to eutrophic waters in the temperate zone. Klebs reported this species from Java [37]. This species has numerous reports due to its appearance in many protistan guidebooks. In addition to the original four drawings, 14 additional drawings and two published photographs are available. Popovsky & Pfiester declared *G. viride* Penard 1891, *G. acidotum* Nygaard 1949, *G. p. dorhni* Wawrik and *G. campaniforme* Popovsky 1971 to be synonymous with *G. aeruginosum* Stein 1883 [12]. *G. campaniforme* Popovsky was described from material collected from a drinking-water reservoir in the Czech Republic [38]. *G. viride* Penard was described from Switzerland [39]. *G. acidotum* Nygaard was described from Danish ponds [40]. *G. p. dohrni* Wawrik was described from Austrian fish ponds [41]. Of these three, *G. campaniforme* is the only one that has not been observed since its original description outside the synonymy.
8. ***Gymnodinium aesculum*** Baumeister 1943* – This species was described by Baumeister from German waters in a 552 word description in German and has not been observed again [42]. The description included four drawings, some cell measurements and was based on several individuals.
9. ***Gymnodinium aestivale*** Skvortzov 1968* – This species was described by Skvotzov from Northern Manchuria, China [43]. His 69 word, Latin and English description was accompanied by length and width measurements of the cell and one drawing. This species has not been observed since description.
10. ***Gymnodinium affine*** Dogiel 1906* – This species was described by Dogiel from cysts in the Gulf of Naples [44]. His 433 word description in German contained four drawings. This species has not been observed since its original description.
11. ***Gymnodinium agaricoides*** **Campbell 1973**– This species was described by Campbell from the polyhaline portion of Gales Creek, North Carolina, USA by observing several individuals in eight samples [45]. He included three drawings and some quantitative measurements in his 216 word description in English. It has since been observed in Greek waters [46] and the Chesapeake Bay [47].
12. ***Gymnodinium agiliforme*** **Schiller 1928**– This species was described by Schiller from the Adriatic Sea [48]. He gave four drawings in his 177 word, Latin and German description which also contained some quantitative information about size of the cells and their habitat. He reported the species again with no new observations [21]. In 1982 this species was observed in the subarctic Pacific [49]. In 1998 the species was observed in Russian waters [36]. It was observed in Romania [50], Spain [51] and the Sea of Okhostk [52]. There are 281 observations of *G. agiliforme* within the GBIF database.
13. ***Gymnodinium alaskensis*** Bursa 1963* – This species was described by Bursa from small freshwater ponds near Barrow, Alaska [53]. He viewed several cells and gave three drawings in his 316 word description in English that includes quantitative and qualitative cell morphology information. This species has not been observed since its first description.
14. ***Gymnodinium allophron*** Larsen 1994* – Larsen described this species from Hobson’s Bay (marine waters), Australia using eight living cells [54]. His 234 word, Latin and English description included four photos and one drawing. The description also contained quantitative measurements of cell size. It has not been observed since.
15. ***Gymnodinium amphiconicoides*** Schiller 1957* – Schiller described this species from material collected from freshwater Lake Neusiedl [15]. He observed at least two individuals, because he gives a range of measurements, but does not specify how many cells he observed. Three drawings were given in his 104 word description in German. This species has not been observed since.
16. ***Gymnodinium amphityphlum*** Larsen 1994* – Larsen described this species from marine, Australian waters [54]. He observed over 20 living cells to draft his 353 word, Latin and English description. He gave three photos, one drawing and quantitative measurements of cell size. This species has not been observed since.
17. ***Gymnodinium amphora*** **Kofoid & Swezy 1921**– This species was described by Kofoid and Swezy from La Jolla, California [19]. They observed only one cell and gave two high quality drawings, quantitative measurments of morphology and a 544 word description in English. The species was reported by Schiller with no new observations or images [21]. It has been observed in the Mediterranean Sea [55] and the Gulf of Mexico [56].
18. ***Gymnodinium amplinucleum*** **Campbell 1973**– This species was described by Campbell from the polyhaline section of Gales Creek in North Carolina, USA [45]. At least two individuals were observed in one sample. Campbell gave two drawings and a 210 word description in English containing quantitative measurements of cell size. This species has also been observed in the Chesapeake Bay [47].
19. ***Gymnodinium antarcticum*** **Thessen, Patterson and Murray** nom. nov. See *Gymnodinium frigidum* Balech 1965.
20. ***Gymnodinium arcticum*** **Wulff 1919**– This species was described by Wulff from the Barents Sea [57]. He gave four drawings and a 126 word description in German. He also gave a range of cell measurements so it is assumed that he saw at least two cells. Schiller and Lebour reported *G. arcticum*, but did not observe it [20], [21]. However, the species has been observed in the Strait of Georgia [58], near Japan [59], in Plymouth Sound [60], near Svalbard [61], off the east coast of the USA [62], in the Aegean Sea [63], the Russian Arctic [64], the Chesapeake Bay [65], in the Black Sea [66] and near Russia [36]. There are 136 records of this species in GBIF. There are a total of nine published drawings available and no photographs.
21. ***Gymnodinium arcuatum*** **Kofoid 1931**– This species was described by Kofoid [67]. In his 297 word English description, he did not give a range for the cell length and width, but did state that the species was common in Mutsu Bay, Japan. We conclude that while Kofoid saw many of this species, the actual description and measurements are based on only one cell. In 1933, Schiller reported the species without making new observations [21]. Sixty years later, Konovalova observed the species and gave two new drawings [36]. It was also observed in the Strait of Taiwan [68] and in the Black Sea [69]. There are three drawings and no photographs available.
22. ***Gymnodinium arenicolus*** **Dragesco 1965**– This species was described by Dragesco in from the sands off Roscoff, France [70]. His 885 word description in French was based on many cells and included nine drawings and cell measurements. This species has also been known as *G. arenicola* and *G. arenicolum* (Appendix S2). It has been observed in British waters [60].
23. ***Gymnodinium armoricanum*** Villeret 1953* – This species was described by Villeret from Lande d’Ouée, France [71]. He gave a 192 word description in French including cell measurements, habitat information and six drawings. This species has not been observed since its description.
24. ***Gymnodinium atomatum*** Larsen 1994* – Larsen described this species based on observations of six living cells from marine, Australian waters [54] as part of a broader survey [72]. His 296 word, English and Latin description contained two photographs, one drawing and cell morphology measurements. This species has not been observed since its original description.
25. ***Gymnodinium attenuatum*** **Kofoid & Swezy 1921–** This species was described by Kofoid and Swezy from material collected off La Jolla, California [19]. Their 464 word, Enlgish-language description was based on observations of three individuals. They provided one line drawing and quantitative measurements of cell morphology. Schiller reported the species, but made no new observations [21]. This species has been observed in the Mediterranean Sea [55].
26. ***Gymnodinium aurantium*** **Campbell 1973–** This species was described by Campbell from the mesohaline portion of Gales Creek, North Carolina, USA [45]. He provided a 167 word description in English with cell size measurments and four drawings. There is possibly some confusion between this species and *Pfiesteria piscicida* because the details needed to distinguish them are not observable via light microscopy [73]. This species has been observed in the Chesapeake Bay [74].
27. ***Gymnodinium auratum*** **Kofoid & Swezy 1921–** This species was described by Kofoid and Swezy from observations of one cell [19]. It was collected off La Jolla, California. The authors gave a 562 word description in English with two drawings and quantitative cell measurements. Schiller reported the species with no new observations [21]. It has been observed in the Gulf of Mexico [56], Mediterranean Sea [55], the Mexican Pacific [75] and the Black Sea [76].
28. ***Gymnodinium aureolum*** **(Hulburt) Hansen 2000–** This species was originally described as *Gyrodinium aureolum* by Hulburt from marine waters near Woods Hole, Massachusetts, USA [77]. Campbell observed the cells in the polyhaline portion of Gales Creek, North Carolina, USA in 1973 [45]. It was then transferred to *Gymnodinium* by Hansen based on observations of laboratory cultures [78]. Hansen provides a 1000+ word description with 18 drawings and photographs. The morphology and phylogeny of this species was thoroughly treated by Tang and co-workers using cells cultured from the Elizabeth River, Virginia, USA [79]. This group provided additional photographs of this species. Cultures are available from the Cawthron Institute Culture Collection of Microalgae and held at the University of Tasmania School of Plant Science Algal Culture Collection.
29. ***Gymnodinium aureum*** **Kofoid & Swezy 1921**– This species was described by Kofoid and Swezy from the marine waters near La Jolla, California [19]. Their 632 word description in English was based on observations of two cells and included quantitative morphological information. Schiller reported the species with no new observations [21]. Since its description, it has been observed in New Jersey waters [80], Yucutan, Mexico [81], the Mediterranean Sea [55] and one cell was observed in San Diego Bay [82]. *Gyrodinium aureum* was later synonymized with this species [19].
30. ***Gymnodinium australe*** **Playfair 1919**–This species was described by Playfair from freshwater in Sydney, Australia by observing many individuals [33]. His 300 word, Latin and English description included cell measurements and three drawings. A previous name for this species is *Gymnodinium fuscum* var. *cornifax* (Schilling) Playfair. This species has been reported by Day et al. [83]. It has been observed in multiple locations across New South Wales, Australia [33].
31. ***Gymnodinium australense*** Ruinen 1938* – This species was described by Ruinen from Australia by observing many cells [84]. The description is 193 words long and in German. Ruinen gave four drawings and cell measurements. This species has not been seen since its description and no photographs are available.
32. ***Gymnodinium austriacum*** **Schiller 1933**– This species was described by Schiller from freshwater Lake Attersee in Austria [21]. His description was 119 words long and in German with six drawings and was based on observations from 45 cells. He included cell size measurements and habitat information. Popovsky and Pfiester synonymized *G. tridentatum* Schiller, *G. cruciatum* Thompson, *G. thompsonii* (Thompson) Kiselev, *G. waltzii* Baumeister, *G. titubens* Christen and *G. autumnale* Christen with this species [12]. However, the images for *G. austriacum* Schiller and *G. cruciatum* Thompson do not resemble each other, meaning that the Popovsky and Pfiester synonymy could be wrong. This species has been observed in Japanese waters [34], Ohio, USA [85] and the Czech Republic [86].
33. ***Gymnodinium baccatum*** **Balech 1965**– This species was described by Balech from Antarctica by observing many individuals [87]. His 331 word description in English was accompanied by two drawings and contained cell size measurements. In 1976 Balech again observed the species and provided another drawing [88]. There is one record of this species in GBIF. This species has been observed in the Mediterranean Sea [55].
34. ***Gymnodinium baicalense*** **Antipova 1955**– This species was described from Lake Baikal, Russia and is said to be endemic to this area [89]. The 315 word, Russian description gives quantitative cell size measurements and three drawings. This species has been observed numerous times in Lake Baikal, Russia [90], [142], but nowhere else to date. Later work provided fuller statements of cell morphology and life cycle, stating that *Gymnodinium baicalense* var. *minor* Antipova is really a life stage of *G. baicalense* Antipova [91]. Published drawings and photographs are available. Five sequences are available in GenBank under the name *Gymnodinium* sp. (FJ024300, FJ024301, FJ024302, FJ024303, FJ024304). Phylogenetic analysis shows that it is most closely related to *Gymnodinium aureolum* (Hulburt) Hansen [92].
35. ***Gymnodinium baumeisteri*** Schiller 1957* – This species was described by Schiller from freshwater Lake Neusiedl, Austria [15]. He did not specify how many cells were observed to write the description, but since a range of measurements were given for the length and width we can assume he observed at least two cells. His 224 word description in German was accompanied by four drawings. This species has not been observed since its description.
36. ***Gymnodinium biciliatum*** **Ohno 1911**– This species was described by Ohno from a freshwater pond in Japan [93]. His 73 word description in German was offset by 37 drawings. This species was unique in the presence of three flagella, two of which were longitudinal. Kofoid and Swezy discussed the possibility that the appearance of two flagella was an optical illusion caused by rapid movement of the flagella in living cells [19]. Schiller reported the species with no new observations [21]. In 1970, Bicudo and Skvortzov observed *G. biciliatum* in Brazilian waters, but make a point to mention that their cells definitely had one longitudinal flagellum [94]. Popovsky and Pfiester also reported the species, but say nothing about the flagella [12]. They stated that the species has been observed in Japan and South America.
37. ***Gymnodinium biconicum*** **Schiller 1928**– This species was described by Schiller from the Adriatic Sea [48]. He did not specify how many cells were observed to write the description, but there must have been at least two. His 92 word, Latin and English description included cell measurements and one drawing. Schiller [21] reported the species again but with no new observations. Wood observed the species in Australian waters [95]. It has been observed in the Gulf of Mexico [56], the Black Sea [66], the Mediterranean Sea [55] and on the east coast of the USA [62]. This species has 14 records listed in GBIF.
38. ***Gymnodinium bicorne*** **Kofoid & Swezy 1921**– This species was described by Kofoid and Swezy from La Jolla, California, USA [19]. Their 560 word description in English was based on one individual and was accompanied by two detailed drawings and morphological measurements. The species was observed again by Wailes, but was labeled as “scarce” [58]. This species has been seen in the tropical Atlantic [96].
39. ***Gymnodinium bifurcatum*** Kofoid & Swezy 1921* – This species was described by Kofoid and Swezy from marine waters near La Jolla, California, USA [19]. Their 690 word description in English was based on one individual and was accompanied by two detailed drawings. They gave an extensive, quantitative morphological characterization of the single observed cell. This species was reported by Schiller despite having no new observations [21]. It has not been observed since its first description.
40. ***Gymnodinium bilobatum*** van Meel 1969* – This species was described by Van Meel from Belgium [97]. His 146 word description in French included two drawings and was based on one individual. Cell size measurements were given. This species has not been observed since its description.
41. ***Gymnodinium birotundatum*** **van Goor 1925**– This species was described by Van Goor from oligohaline Dutch waters [98]. He did not specify the number of cells used to craft the description, but gave a range for length and width, so we can assume there were at least two cells involved. The description was over 1000 words long and included one drawing, cell size measurements and habitat description. Conrad and Kufferath observed this species in mesohaline waters in Belgium [18]. It has also been observed in British waters [60].
42. ***Gymnodinium bisaetosum*** Lindemann 1928* – This species was described by Lindemann from a German lake [99]. His 61 word description in German contained one drawing and no cell measurements. It was described entirely from cysts and has not been observed since.
43. ***Gymnodinium boguensis*** **Campbell 1973**– This species was described by Campbell from Gales Creek, North Carolina, USA by observing at least two cells [45]. The 138 word description in English has two drawings and cell morphology measurements. It has been observed in the Chesapeake Bay [65].
44. ***Gymnodinium bonaerense*** **Akselman 1985**– This species was described by Akselman in from the coast of Argentina [100]. His 1000+ word, Latin and Spanish description includes quantitative information about cell morphology and habitat. He included three drawings and three photographs. It has not been observed in the field since its description; however, type material was deposited at the National Institute of Fisheries Research and Development in Argentina (INIDEP). The authors cannot confirm that this material is available to other researchers.
45. ***Gymnodinium caerulescens*** Schiller 1957* – This species was described by Schiller from freshwater Lake Neusiedl, Austria [15]. His 92 word description in German was based on several individuals and was accompanied by two drawings. He gave some quantitative cell measurements. It has not been observed since.
46. ***Gymodinium campbelli*** **Thessen, Patterson & Murray 2012** - Campbell gave an account of a species that he called *Gymnodinium translucens* from the polyhaline portion of Gales Creek, North Carolina, USA [45]. His account was accompanied by a drawing that does not match the drawings in Kofoid and Swezy’s description of *G. translucens*. As we think Campbell used this name by mistake, we have (below) re-named this species *Gymnodinium campbelli* Thessen, Patterson & Murray. This species has been observed in the Chesapeake Bay [47].
47. ***Gymnodinium canus*** **Kofoid & Swezy 1921**– This species was described by Kofoid and Swezy from a single cell found near La Jolla, California [19]. Their 650 word description in English was accompanied by three detailed drawings and quantitative morphological details. Schiller reported the species in German with no new observations [21]. It has been observed in the Mediterranean [55] and the Black Sea (http://phyto.bss.ibss.org.ua/wiki/Gymnodinium_canus).
48. ***Gymnodinium capitatum*** **Conrad & Kufferath 1954**– This species was described by Conrad and Kufferath from Belgium [18]. Their 248 word description in French was based on observations of one cell and included two line drawings and one approximate height measurement. It has been observed in British waters [60] and in the sediments of Gwangyang Bay, South Korea [101].
49. ***Gymnodinium caput*** **Schiller 1928**– This species was described by Schiller from the Adriatic Sea and reported again later with no new observations [48], [21]. The original description contained information from several cells, five drawings and quantitative cell sizes. This species has been observed in the Mediterranean Sea [55].
50. ***Gymnodinium cassiei*** Norris 1961* – This species was described by Norris from New Zealand [102]. He used at least two cells to craft his 172 word, Latin and English description that included one image. Basic cell size measurements were given. It has not been observed since.
51. ***Gymnodinium catenatum*** **Graham 1943**– This species was originally described by Graham from a bloom in the Gulf of California, with a 384 word description in English [103]. This species is a known producer of toxins and is thus heavily studied. It has been observed many times all over the world [104]. There are 122 occurrence records in GBIF and 102 sequences in GenBank. Cultures are available from the Australian National Algae Culture Collection, the Canadian Center for the Culture of Microorganisms, the Scandinavian Culture Collection of Algae and Protozoa, the Microbial Culture Collection – Japan, the Provasoli-Guillard National Center for Culture of Marine Phytoplankton and the Cawthron Institute Culture Collection of Microalgae. Cultures are also held at University of Tasmania School of Plant Science Algal Culture Collection, but are not for sale.
52. ***Gymnodinium chiastosporum*** **(Harris) Cridland 1958**– This species was first described as *Tetrodinium chiastosporum* by Harris from a freshwater pond in the UK [105]. This was based on observations of a non-motile stage. Later, Cridland noticed, in the same location, that the motile phase of this species was a *Gymnodinium*, and named it *Gymnodinium hippocastanum* [106]. This 651 word, Latin and English description contained three drawings and quantitative morphological measurements. Poposvky and Pfiester drew together *Dinastridium chiastosporum*, *Gymnodinium hippocastanum*, *Dinastridium sexangulare* and *Tetradinium chiastosporum* under *G. chiastosporum* [12]. They also mentioned that this species has been observed in Great Britain and the Czech Republic. There are three records in GBIF.
53. ***Gymnodinium chinensis*** **Thessen, Patterson and Murray** nom. nov. See *Gymnodinium frigidum* Skvortzov 1968.
54. ***Gymnodinium chukwanii*** Ballantine 1961* – This species was described by Ballantine from a freshwater fish pond in Zanzibar [107]. The 485 word, English and Latin description was based on many cells and provided four drawings and cell size measurements. It has not been reported since.
55. ***Gymnodinium cinctum*** **Kofoid & Swezy 1921**– This species was described by Kofoid and Swezy from marine waters near La Jolla, California, USA [19]. The 330 word description in English mentions finding the species three times, but the length and width measurements were not given as a range. This suggests that either the cells were remarkably similar in size or the description was based on only one of the found individuals. Two drawings were included. Schiller provided a German account of the species with no new observations [21]. Wood observed the species in Australian waters and included a new drawing [95]. Hada observed the species as cysts in Antarctic waters [108]. He gave a new drawing and measurements. However, the images in Kofoid and Swezy and Hada do not look like the same species [19], [108], Hada 1970). That could be because Hada observed a cyst while Kofoid and Swezy observed a vegetative cell. It is not clear how Hada knew the cyst he observed was *G. cinctum* Kofoid & Swezy 1921. It has been observed in Japanese waters [109], the Mediterranean Sea [55], the Black Sea [66] and the Gulf of Mexico [56].
56. ***Gymnodinium cnecoides*** **Harris 1940**– This species was described by Harris from a freshwater pond in the UK by examining one cell [105]. Popofsky and Pfiester synonymized *Gymnodinium saginatum* and *Gymnodinium luteofaba* with this species [12]. They also report that the species has been found in Great Britain and Poland. There is one GBIF record. It has been reported in Lake Tovel, Italy [110], a swamp in the Czech Republic [86], Lake Gölköy, Turkey [111], a bog in Wisconsin [112], and the Chesapeake Bay [47].
57. ***Gymnodinium cnodax*** Conrad & Kufferath 1954* – This species was described by Conrad and Kufferath from Belgium [18]. Their 147 word description in French was based on one cell and has one drawing. Cell size measurements and some habitat information were given. It has not been seen since.
58. ***Gymnodinium coeruleum*** **Dogiel 1906**– This species was described by Dogiel from saline waters in the Gulf of Naples [44]. His 467 word description in German was based on observations from two cells. It contained two drawings but no cell measurements. This species was observed in marine waters off La Jolla, California, USA [19]. The record of the California observations was accompanied by detailed cell measurements. Schiller reported the species with no new observations [48]. Wood observed it in Australian waters, but called it *Gymnodinium coerulatum* Dogiel [95]. This species should not be confused with *G. coeruleum* Antipova 1955 which was described from Lake Baikal, Russia and was later observed in the Angara River Basin, Russia [89], [113]. Molecular data show that *Gymnodinium coeruleum* Antipova (GenBank # FJ024299) is closely related to *Gyrodinium helveticum* (Penard) Takano & Horiguchi 2004 and morphological observations also align the two entities [92]. *G. coeruleum* Dogiel has been observed in the Chesapeake Bay [47].
59. ***Gymnodinium colymbeticum*** **Harris 1940**– This species was described by Harris from a freshwater pond in Reading, UK [105]. His 103 word, Latin and English description contained two drawings and cell size measurements. Popovsky and Pfiester synonymized *Gymnodinium pulvisculus* Klebs 1912 with this species, but made no new observations [12] They also noted that *Gymnodinium pulvisculus* Pouchet 1885 was not *G. colymbeticum*, but was a synonym of *Oodinium poucheti* (Lemmerman) Chatton 1912. This species has not been observed and labeled as *G. colymbeticum* since its description. However, observations of *G. pulvisculus* could be observations of this species or *Oodinium poucheti*.
60. ***Gymnodinium concavum*** Skvortzov 1968* – This species was described by Skvortzov from Northern Manchuria, China [43]. His 135 word, Latin and English description gave cell measurements and two drawings. This species has not been observed since description.
61. ***Gymnodinium conicum*** **Kofoid & Swezy 1921**– This species was first described as *Gymnodinium viridis* by Lebour from a single specimen in Plymouth Sound, England [16]. Kofoid and Swezy synonymized it with *Gymnodinium conicum* without making any new observations [19]. They reused the Lebour drawing in their publication and gave a 384 word, English account. The Lebour description gave one quantitative measurement, so the additional measurements in Kofoid and Swezy must be calculated from the drawings. Lebour and Schiller reported the species with no new observations [20], [21]. It was not until 1938 that Conrad and Kufferath observed the species in Belgium, but no new images were created [18]. They gave new cell size measurements and a brief description of habitat. Dodge reported the species but made no new observations [24].
62. ***Gymnodinium contractum*** Kofoid & Swezy 1921* – This species was described by Kofoid and Swezy from seven cells collected in marine waters near La Jolla, California, USA [19]. Their 539 word description in English contained two detailed drawings and morphological measurements. Schiller gave an account in German with no new observations [21]. It has not been seen since.
63. ***Gymnodinium corii*** **Schiller 1928**– This species was described by Schiller from the Adriatic Sea [48]. His 115 word, German-language description was accompanied by three drawings and was based on at least two cells. He gave some cell size measurements and a brief habitat description. Schiller again reported this species in 1933, but with no new observations [21]. It has been reported from the South China Sea [114] and the Mediterranean Sea [55]. There are four sequences available in GenBank.
64. ***Gymnodinium corollarium*** **Sundström, Kremp & Daugbjerg 2009**– This species was described from the Baltic Sea [115]. The Latin and English description was over 1000 words long and contains several photographs from a light and electron microscope. The description of cell morphology and ultrastructure in addition to an investigation of the habitat and physiology was extensive. A type culture is available. There is one sequence available in GenBank. It has not been reported from any additional locations, possibly because it is a relatively new species. Cultures are available from the Scandinavian Culture Collection of Algae and Protozoa.
65. ***Gymnodinium corpusculum*** (Perty) Saville-Kent 1880/81* – This species was originally described by Perty as *Peridinium corpusculum* from freshwater in Switzerland [116]. Perty included one drawing and one length measurement. This species was transferred to *Gymnodinium* by Saville-Kent [117]. His 46 word description in English did not contain images. This species has not been observed since Perty.
66. ***Gymnodinium costatum*** **Kofoid & Swezy 1921**– This species was described by Kofoid and Swezy near the marine waters of La Jolla, California, USA from observations of many cells [19]. It was later reported by Schiller with no new observations [21]. This species has been observed in Australian waters [95], the Gulf of Mexico [56], the Chesapeake Bay [47], the Mexican Pacific [75] and the Mediterranean Sea [55]. There are two published drawings and one scanning electron micrograph of this species available online.
67. ***Gymnodinium cryophilum*** **(Wedemayer, Wilcox & Graham) Hansen & Moestrup 2000**– This species was described by Wedemayer, Wilcox and Graham as *Amphidinium cryophilum* using a 1000+ word, Latin and English description and 11 images [118]. Hansen and Moestrup transferred it to *Gymnodinium* [7]. As *A. cryophilum* Wedemayer, Wilcox & Graham 1982, the species had its morphology and behavior thoroughly characterized [118]. Several drawings and photographs are available. A type culture was deposited at the University of Wisconsin, but the authors cannot confirm that it is available to other researchers.
68. ***Gymnodinium cucumis*** **Schütt 1895**– This species was described by Schütt from near the Mediterranean Sea [119]. His 97 word description in German contained seven quality drawings, but little quantitative information. Since then, it was reported again in the Mediterranean Sea [55].
69. ***Gymnodinium cyaneofungiforme*** **Conrad & Kufferath 1954**– This species was described by Conrad and Kufferath from mesohaline Belgian waters [18]. Their 157 word description in French was based on observations of one individual and contained some cell size measurements, habitat information and three drawings. This species has one record in GBIF.
70. ***Gymnodinium cyaneum*** Schiller 1957* – This species was described by Schiller from the freshwater Lake Neusiedl, Austria [15]. His 96 word description in German was accompanied by three drawings and was based on observations of one individual. Cell size measurements were given. This species has not been observed since its description.
71. ***Gymnodinium danicans*** **Campbell 1973**– This species was described by Campbell from Gales Creek, North Carolina, USA [45]. His 307 word description in English contained nine drawings. It has also been observed in the lower Chesapeake Bay and its tributaries [74], [120], [121] and in Australia [13]. Note, this species should not be confused with *Gymnodinium danicas* Casto-Sánchez 1998, which is a erroneous name and may be an observation of *Peridiniella danica* in the Mexican Pacific [75].
72. ***Gymnodinium danubiense*** Schiller 1957* – This species was described by Schiller from freshwater near Vienna, Austria [15]. His 103 word, German-language description is based on observations from at least two individuals and is accompanied by two drawings and cell size measurements. It has not been observed since its description.
73. ***Gymnodinium deformabile*** Schiller 1957* – This species was described by Schiller from freshwater near Vienna, Austria [15]. His 109 word, German-language description was based on many cells and is accompanied by three drawings. Cell size measurements were given. It has not been seen since its description.
74. ***Gymnodinium dentatum*** **Larsen 1994**– This species was described by Larsen from Australian marine waters [54]. His 336 word description in English was based on observations of 12 living cells and contained five images. It has also been reported from the Beaufort Sea [122].
75. ***Gymnodinium depressum*** Skvortzov 1968* – This species was described by Skvortzov from Northern Manchuria, China [43]. His 81 word, Latin and English description included cell measurements and one drawing. This species has not been observed since its description.
76. ***Gymnodinium devorans*** Schiller 1957* – This species was described by Schiller from freshwater Lake Neusiedl, Austria [15]. His 128 word description in German was based on many cells and was accompanied by six drawings and cell measurements. It has not been seen since its description.
77. ***Gymnodinium diamphidium*** Norris 1961* – Norris described this species from New Zealand using observations made on one individual [102]. His 394 word description in Latin and English contained four drawings and cell size measurements. It has not been observed since.
78. ***Gymnodinium diploconus*** **Schütt 1895**– This species was described by Schütt from around the Mediterranean Sea [119]. There was no explicit text description, but there were four drawings with informative captions. Quantitative measurements are missing. There are six records of this species in GBIF.
79. ***Gymnodinium discoidale*** **Harris 1940**– This species was described by Harris from a freshwater pond in Reading, UK [105]. His 152 word, description in Latin and English was based on one cell and was accompanied by three drawings and some cell measurements. Popovsky and Pfiester synonymized *Glenodinium eurystomum* Harris with this species and stated that it had been found in Great Britain, Czech Republic and Germany [12]. This species has two records in GBIF. There are four unique drawings and no photographs published.
80. ***Gymnodinium dissimile*** **Kofoid & Swezy 1921**– This species was described by Kofoid and Swezy from the marine waters near La Jolla, California, USA [19]. Their 515 word description in English was based on one cell, contained a quantitative morphological description and was accompanied by two detailed drawings. Schiller gave a description in German without new observations [21]. This species has been observed in the Gulf of Mexico [56], Chesapeake Bay [47], and the Mediterranean Sea [55].
81. ***Gymnodinium dodgei*** Sarma & Shyam 1974* – This species was described by Sarma and Shyam from pools of water in India [123]. Their 271 word description in English was based on many individuals and included five images (drawings and photographs). Cell size measurements were given. It has not been seen since its original description.
82. ***Gymnodinium dogieli*** Kofoid & Swezy 1921* - This species was described by Kofoid and Swezy from marine waters near La Jolla, California, USA [19]. Their 847 word, description in English was based on many cells and was accompanied by three detailed drawings and quantitative morphological description. Schiller gave an acount in German without new observations [21]. This species has not been seen since its description.
83. ***Gymnodinium doma*** Kofoid & Swezy 1921* – This species was described by Kofoid and Swezy from marine waters near La Jolla, California, USA [19]. Their 627 word, English-language description was based on observations of one cell and contained two detailed drawings and quantitative morphological measurements. Schiller gave an account in German with no new observations [21]. This species has not been observed since its description.
84. ***Gymnodinium dorsalisulcum*** **(Hulburt, McLaughlin & Zahl) Murray, de Salas & Hallegraeff 2007**– This species was originally described as *Katodinium dorsalisulcum* by Hulburt, McLaughlin and Zahl and was later transferred to *Gymnodinium* by Murray, de Salas and Hallegraeff who observed it in Australian waters [77], [124]. Their 1000+ word, English-language description contained five photographs and extensive morphological and molecular characterization. There are three sequences available in GenBank. A culture is available from the Australian National Algae Culture Collection.
85. ***Gymnodinium endofasciculum*** **Campbell 1973**– This species was described by Campbell from Gales Creek, North Carolina, USA [45]. His 183 word, description in English was based on at least four cells and contained two drawings. This species has been found in Spanish waters [125], near Spitzbergen [126], in the Baltic Sea (http://test.b-neat.org/species_sheet/?id=1000882) and in the Chesapeake Bay [47]. It has one record in GBIF.
86. ***Gymnodinium enorme*** **Ballantine 1964**– This species was described by Ballantine from British waters using a 16 word description in English with no images or measurements [127]. She synonymized *Gymnodinium irregulare* Conrad & Kufferath with this species.As *Gymnodinium irregulare* is preoccupied, we rename (below) *Gymnodinium irregulare* Conrad & Kufferath 1954 as *Gymnodinium conkufferi* Thessen, Patterson and Murray 2012. This species has one occurrence record in GBIF.
87. ***Gymnodinium eucyaneum*** Hu 1983* – This species was originally described in Chinese as *Gymnodinium cyaneum* by Hu [128]. This name was already occupied, so in 1983 it was changed to *Gymnodinium eucyaneum* Hu [129]. This 136 word, Latin and English description was based on many cells from Wuchang, China and contained a photograph from a light microscope and a drawing. Cell size measurements were given. It has not been observed since its description.
88. ***Gymnodinium eufrigidum S*** chiller 1957* – This species was described by Schiller from freshwater near Vienna, Austria [15]. His 134 word, description in German is accompanied by two drawings and was based on observations of many cells. Cell measurements were given. It has not been observed since its description.
89. ***Gymnodinium excavatum*** **van Meel 1969**– This species was described by Van Meel from Belgium [97]. His 80 word description in French was based on one cell and was accompanied by one drawing and cell size measurements. It has been reported from Chinese waters [130], Lake Geneva [131] and the Black Sea [69]. This species has one record in GBIF.
90. ***Gymnodinium exechegloutum*** Norris 1961* – This species was described by Norris from the waters around New Zealand [102]. His 262 word description in Latin and English was based on at least two cells and was accompanied by one drawing. The description gave some cell size measurements. It has not been observed since.
91. ***Gymnodinium filum*** **Lebour 1917**– This species was described by Lebour from Plymouth Sound, England [16]. Her 79 word, description in English was based on one cell and was accompanied by two drawings. Kofoid and Swezy, Schiller and Dodge all report the species with no new observations [19], [21], [24]. This species has been observed on the east coast of the USA [62], the Mediterranean Sea [55], [132] and in Scandinavian waters (http://nordicmicroalgae.org/).
92. ***Gymnodinium flavum*** **Kofoid & Swezy 1921**– This species was described by Kofoid and Swezy from the marine waters near La Jolla, California, USA [19]. Their 637 word description in English was based on observations of many cells and was accompanied by two drawings and quantitative morphological measurements. Schiller wrote a German description with no new observations [21]. Wood observed the species in Australian waters [95]. Balech and Kopczyńska observed the species in Antarctic waters [88], [133]. This species has also been observed in the Gulf of Mexico [56], Delaware Bay [134], the Black Sea [66], the Mediterranean Sea [55] the Chesapeake Bay [47] and has been seen several additional times in La Jolla, California, USA [135]. This species is known to discolor the water yellow when it reaches bloom concentrations.
93. ***Gymnodinium fossarum*** **Conrad & Kufferath 1954**– This species was described by Conrad and Kufferath from Belgium [18]. Their 259 word, French-language description was based on observations of one cell and was accompanied by three drawings. Cell size measurments and a brief description of the habitat were given. It has not been observed since its description.
94. ***Gymnodinium frigidum*** Woloszynska 1952* – This species was described by Woloszynska from a lake in the Tatra mountains, Poland [136]. There was no text description, but one drawing was included with a four-word caption in Polish “Tatry, Morskie Oko. Przetrwalnik” describing the location where the species was found. This species should not be confused with *G. frigidum* Balech 1965 or *G. frigidum* Skvortzov 1968 which are both homonyms that are renamed below.
95. ***Gymnodinium frigidum*** **Skvortzov 1968**– This species was described from Northern Manchuria, China [42]. A 97 word description was given in Latin and English and accompanied by one drawing. It has not been observed since. This species should not be confused with the homonyms *G. frigidum* Woloszynska 1952 or *G. frigidum* Balech 1965. We provide the new name *Gymnodinium chinensis* for this species.
96. ***Gymnodinium frigidum*** **Balech 1965**– This species was described from Antarctica with a 230 word description in English [87]. Two drawings are included. It has been observed in the Arctic region (http://dw.sfos.uaf.edu/rest/metadata/ArcOD/2007P6), the Pacific near Russia [137] and the Black Sea [69]. This species has 36 GBIF observations. This species should not be confused with the homonymous *G. frigidum* Woloszynska 1952 or *G. frigidum* Skvortzov 1968. We provide the new name *Gymnodinium antarcticum* for this species.
97. ***Gymnodinium fukushimai*** Hada 1966* – This species was described from a sample collected at McMurdo, Antarctica [138]. The 126 word description in English included cell measurements and one drawing. This species has not been observed since its description.
98. ***Gymnodinium fulgens*** **Kofoid & Swezy 1921**– This species was observed at two different times by Lebour in Plymouth Sound, UK [16]. She labeled it as *Gymnodinium pseudonoctiluca* Pouchet 1885. Kofoid and Swezy, based on differences in cell morphology in the figures, later separated *G. pseudonoctiluca sensu* Lebour 1917 from *G. pseudonoctiluca* Pouchet 1885 and renamed the Lebour version *G. fulgens* [19]. They did this without making direct observations of cells. They gave a 429 word, English-language description with one drawing. It has not been observed since 1917, but since Lebour observed the species on two separate occasions, we do not regard this as a oncer.
99. ***Gymnodinium fuscum*** **(Ehrenberg) Stein 1883**– This species was originally described as *Peridinium fuscum* [139]. It was transferred to *Gymnodinium* by Stein [31]. Popovsky and Pfiester synonymized *Gymnocystodinium gessneri* Baumeister, *Cystodinium gessneri* (Baumeister) Bourrelly and *Gymnodinium caudatum* Prescott with this species [12]. This is a very common, cosmopolitan freshwater species that has been observed many times (see [136], [108], [137], [138], [140] for some recent examples). It is the type species for the genus *Gymnodinium*. Hansen et al. enhanced the original description of this species with light and electron microscopical observations [143]. A culture is available from the Provasoli-Guillard National Center for Culture of Marine Phytoplankton.
100. ***Gymnodinium fusiforme*** **Kofoid & Swezy 1921**– This species was first described as *Spirodinium fusus* by Meunier from Arctic waters (Meunier 1910). Kofoid and Swezy transferred it to *Gymnodinium* without making any new observations [19]. Since then it has been observed in Arctic waters [144] and North African coastal waters [132].
101. ***Gymnodinium galeaeforme*** **Matzenauer 1933**– This species was described by Matzenauer from the Indian Ocean [145]. His 85 word, description in German was based on observations of one cell and was accompanied by three images and cell size measurements. This species has been observed in Australian waters [95], the Gulf of Mexico [56], the Mediterranean Sea [55] and the Black Sea [66]. There are 15 occurrence records for this species in GBIF.
102. ***Gymnodinium galeatum*** **Larsen 1994**– This species was described by Larsen from Australian marine waters [54]. His 298 word, description in Latin and English was based on observations from 11 living cells and was accompanied by three photographs, one drawing and quantitative cell measurements. He also observed the species in Danish waters. This species has been observed in the Sea of Japan [146] and in the Beaufort Sea [122].
103. ***Gymnodinium galeiforme*** Okolodkov 1997* – This species was described by Okolodkov from the Norwegian Sea [26]. His 324 word, English-language description was based on observations of one cell and contained one image. It has not been reported since its original description.
104. ***Gymnodinium galesianum*** **Campbell 1973**– This species was described by Campbell from Gales Creek, North Carolina, USA [45]. His 230 word, description in English was based on observations of many cells and contained four drawings. This species has one occurrence record in GBIF. It has also been reported from the Chesapeake Bay [74].
105. ***Gymnodinium gelbum*** **Kofoid 1931**– This species was described by Kofoid from the Mutsu Sea in Japan [67]. The 238 word, English-language description was based on two encysted cells, accompanied by one drawing and contained cell size measurements. Schiller gave an account in German with no new observations [21]. It has been observed in the Gulf of Mexico [56], the Mediterranean Sea [55], the Black Sea [66], in Australian waters [95] and off the Indian coast [147]. This species has 24 observations in GBIF. There are two drawings and no photographs published for this species.
106. ***Gymnodinium gibbera*** **Schiller 1928**– This species was described by Schiller from the Adriatic Sea [48]. His 94 word description in German was based on at least two cells, accompanied by two drawings and contained cell size measurements. He reported the species again without new observations [21]. The species has been observed in the Gulf of Mexico [56] and the Black Sea [66]. Two drawings and no photographs of this species have been published.
107. ***Gymnodinium glandiforme*** **Conrad & Kufferath 1954**– This species was described by Conrad and Kufferath from mesohaline Belgian waters [18]. Their 134 word, English-language description was based on one cell and was accompanied by one drawing. They included cell size measurements and a brief habitat description. There is one observation of *G. glandiforme* in GBIF.
108. ***Gymnodinium glaucum*** **Schiller 1957**– This species was described by Schiller from the freshwater Lake Neusiedl, Austria [15]. His 93 word description in German was based on observations of many cells and was accompanied by three drawings. It included cell size measurments and comments on the habitat. This species has been observed in the North Sea [148] and the Black Sea [66].
109. ***Gymnodinium gleba*** **Schütt 1895**– This species was described by Schütt from near the Mediterranean Sea [119]. He did not give a full text description, rather wrote an informative caption for one figure which does not contain cell measurements. One cell of this species was collected and figured by Kofoid and Swezy from marine waters near La Jolla, California, USA [19]. They included an extensive morphological description, giving a range of values for the cell dimensions. This is strange considering they only observed one individual. Drira et al. observed the species in North African coastal waters [132]. Schiller provided a German description with no new observations [21]. There are two detailed drawings published to aid in identification of this species, but no photographs.
110. ***Gymnodinium gracile*** **Bergh 1881/82**– This species was first described by Bergh in German [149]. He gave a lengthy (612 words) text description including quantitative measurements and two drawings. Kofoid & Swezy also observed this species near La Jolla, California, USA, but called it *Gymnodinium abbreviatum* [19]. To add to the confusion, they included *Gymnodinium gracile* Bergh in their species list. Perhaps because the Kofoid and Swezy description was in English, subsequent reports of this species were under the name *Gymnodinium abbreviatum*. *G. gracile* Bergh has been observed world-wide [132], [150], [20], [67], [58], [75] and is considered to be an oceanic species.
111. ***Gymnodinium gracilentum*** **Campbell 1973**– This species was described by Campbell from Gales Creek, North Carolina, USA [45]. His 103 word, description in English was based on observations of many cells and contained four images. The ecology of this species as a mixotroph has been described and a culture has been isolated [151]. This species has been observed in the Baltic Sea (http://test.b-neat.org/species_sheet/?id=1000888) and in the Øresund, Denmark [151].
112. ***Gymnodinium grammaticum*** **(Pouchet) Kofoid & Swezy 1921**– This species was originally described as *Gymnodinium punctatum* var. *grammaticum* from the Atlantic near France [152] and was later emended [19]. The 312 word, English-language description contained a detailed morphological description and one drawing. Schiller reported the species with no new observations [21]. This species has been observed in the Gulf of Naples [153], the Adriatic Sea [48], in Australian waters [95], in the Pacific Ocean near New Zealand [102], in the Gulf of Mexico [56], in the Chesapeake Bay [47] and in the Black Sea [66]. It has 17 observations in GBIF. There are four unique drawings published to aid with identification.
113. ***Gymnodinium granii*** Schiller 1957* – This species was described by Schiller from the freshwater Lake Neusiedl, Austria [15]. His 203 word description in German was based on observations of many cells and was accompanied by seven drawings. He included cell size measurments and some habitat information. It has not been reported since its description.
114. ***Gymnodinium guttiforme*** **Larsen 1994**– This species was described by Larsen from Australian marine waters [54]. His 327 word description in Latin and English was based on observations of eight living cells and was accompanied by three photographs and one line drawing. A detailed morphological description including measurements was given. It has not been observed since its description.
115. ***Gymnodinium guttula*** **(Hada) Balech 1976**– This species was originally described by Hada as *Gymnodinium cinctum* [108]. It was renamed by Balech [88]. The Balech 159 word description in Spanish and English was based on observations of at least two cells and was accompanied by one drawing and cell size measurements. This species has been observed repeatedly in the Southern Ocean [133].
116. ***Gymnodinium hamulus*** **Kofoid & Swezy 1921**– This species was described by Kofoid and Swezy from the beach sands near La Jolla, California, USA [19]. Their 362 word description in English was based on multiple individuals and contained two detailed drawings and quantitative morphological description. Schiller gave an account in German despite having made no new observations [21]. This species has also been observed in the Ría de Vigo in Spain [154].
117. ***Gymnodinium herbaceum*** **Kofoid 1921**– This species was described by Kofoid from the Bay of Naples [19]. His 429 word description in English was accompanied by two drawings and was based on observations of many individuals. One length measurement was given. Schiller reported the species and gave an account in German, but made no new observations [21]. This species has been observed in the Mexican Pacific [75].
118. ***Gymnodinium heterostriatum*** **Kofoid & Swezy 1921**–Kofoid and Swezy observed this species off the coast of La Jolla, California, USA [19]. This species has been observed many times all over the world [20], [67], [58], [95], [60], [155], [82], [156], [36], [64], [157], [126]. A new account was given by Elbrächter [158]. Accounts can be found in French, English, German and Russian. Fourteen photographs are available, but no sequences are in GenBank. This species has 81 records in GBIF.
119. ***Gymnodinium hiemale*** **(Schiller) Popovsky 1990**– This species was first described by Schiller as *Massartia hiemalis* from Rust, Germany during the winter [159]. He gave eight drawings of the species. Popovsky observed the species in Austria, the Czech Republic and Switzerland and transferred it to *Gymnodinium* [12]. He gave a 147 word account in English with cell measurements and four new drawings. Popovsky synonymized *Katodinium hiemale* (Schiller) Loeblich 1965 and *Katodinium intermedium* Christen 1959 with this species. This species should not be confused with *Gymnodinium hiemale* Woloszynska 1917 and *Gymnodinium hiemale* Skvortzov 1927, both of which have been synonymized with *Woloszynskia pascheri* (Suchlandt) von Stosch 1973. There are 12 observation records associated with this name in GBIF, but we are unsure to which *G. hiemale* concept these refer.
120. ***Gymnodinium hiroshimaensis*** **Hada 1968**– This species was first observed by Hada in Japanese waters [160]. It was referred to as *Gymnodinium* sp. and was accompanied by one figure. It was not officially named until 1968 by Hada and described in English (69 words) after being observed a second time in the port of Itsukaichi, Japan [161].
121. ***Gymnodinium huber-pestalozzii*** Schiller 1957* – This species was described by Schiller in freshwater from Vienna, Austria [15]. His 235 word description in German was based on at least two cells and featured four drawings and cell measurements. Schiller synonymized *Gymnodinium austriacum* Schiller in Huber-Pestalozzi [21] with this species despite the fact that the Huber-Pestalozzi report contained no new observations. The synonymy has been ignored throughout the literature. This species has not been observed since its original description.
122. ***Gymnodinium hulburtii*** **Campbell 1973**– This species was described by Campbell from Gales Creek, North Carolina, USA [45]. His 208 word description in English was based on one individual and was accompanied by one line drawing. It has also been observed in the Chesapeake Bay, USA [47].
123. ***Gymnodinium impatiens*** **Skuja 1964**– This species was described by Skuja from Sweden [162]. His 514 word description in German included five drawings. There are two sequences available for this species in GenBank. A strain of this species is available at the Culture Collection of Algae at the University of Cologne.
124. ***Gymnodinium impudicum*** **(Fraga & Bravo) Hansen & Moestrup 2000**– This species was originally described as *Gyrodinium impudicum* by Fraga and Bravo [163]. Hansen and Moestrup renamed it *Gymnodinium impudicum* based on the apical groove structure [7]. This species has been observed in Spanish waters [125], the Mexican Pacific [75] and isolated from South Korean waters (Table 1 in [164]). Phylogenetic studies suggest that some strains of *G. impudicum* are really *G. litoralis* [164]. Sequences with GenBank numbers AF200674 and EF616465 are probably *G. litoralis* [164]. Cultures of this species are available from the Scandinavian Culture Collection of Algae and Protozoa, the Cawthron Institute Culture Collection of Microalgae and the Provasoli-Guillard National Center for Culture of Marine Phytoplankton. Photographs are available.
125. ***Gymnodinium incertum*** **Herdman 1924**– This species was originally described by Herdman from damp sand at Port Erin, Isle of Man, England [165]. Her 77 word description in English was based on one individual and included one line drawing and one basic cell size measurement. This species has also been seen in the Adriatic Sea [48] and the Port of Antifer, France [157]. Schiller and Dodge reported the species with no new observations [21], [24]. It has one occurrence record in GBIF. Two drawings and one photograph are available.
126. ***Gymnodinium incisum*** **Kofoid & Swezy 1921**– This species was described by Kofoid and Swezy from material collected off La Jolla, California, USA [19]. Their 567 word description in English was based on observations of one individual and contained one line drawing. The description contained detailed, quantitative morphological information. Schiller reported the species with no new observations [21]. It has also been observed in the Gulf of Mexico [56] and the Mediterranean Sea [55].
127. ***Gymnodinium incoloratum*** **Conrad & Kufferath 1954**– This species was described by Conrad and Kufferath in Belgian waters [18]. Their 361 word description in French included 12 drawings and was based on at least seven cells. Later it was observed in British waters [60], shrimp ponds in NW Mexico [166], the Chesapeake Bay [47] and in South America [167].
128. ***Gymnodinium inconstans*** van Meel 1969* – This species was described by Van Meel from Belgian waters [97]. His 361 word description in French was based on observations of at least two cells and was accompanied by one line drawing. A range of cell size measurements were given. This species has not been observed since its original description.
129. ***Gymnodinium indicum*** Shyam & Sarma 1975* – This species was described by Shyam and Sarma from a pond in India [168]. Their 88 word description in English was based on several cells and accompanied by nine line drawings. Quantitative cell measurements were given. Popovsky and Pfiester reported this species with no new observations [12]. This species has not been observed since its original description.
130. ***Gymnodinium inerme*** (Schmarda) Saville-Kent 1880/81* – This species was described from Egypt and named *Peridinium inerme* [169]. The description was accompanied by one drawing that lacked many features needed to establish it as a valid species. However, Saville-Kent moved it to *Gymnodinium* [117]. Kofoid and Swezy discussed this problem [19]. Schiller reported the species with no new observations [21]. This species has not been observed since its original description in 1854.
131. ***Gymnodinium instriatum*** **(Freudenthal & Lee) Coats 2002**– This species was originally described by Freudenthal and Lee as *Gyrodinium instriatum* [170]. Coats later renamed this species *Gymnodinium instriatum* based on apical groove configuration of a Chesapeake Bay isolate [7], [171]. This species has been observed in the Mexican Pacific [75]. A culture is available from the Cawthron Institute Culture Collection of Microalgae.
132. ***Gymnodinium intercalaris*** Bursa 1961* ***–*** This species was originally described by Bursa from material collected in the Canadian Arctic [172]. His 253 word description in English was based on observations of at least two individuals and was accompanied by two drawings. One range of cell length measurements was given. This species has not been observed since its original description. It has also been referred to as *G. intercalare*.
133. ***Gymnodinium irregulare*** **Hope 1954** ***–*** This species was described by Hope [173]. He gave a 54 word description in English of cells from Norway that included four drawings. No photographs are available. This species is not to be confused with *G. irregulare* Christen which is a synonym of *G. uberrimum* (Allman) Kofoid & Swezy. As *Gymnodinium irregulare* was preoccupied, we (below) rename *Gymnodinium irregulare* Christen 1959 as *Gymnodinium christenum* Thessen, Patterson & Murray 2012. This species was reported in British waters [60]. It has 27 occurrences in GBIF.
134. ***Gymnodinium japonicum*** **Hada 1974** ***–*** This species was originally described by Hada from material collected in Hiroshima Bay, Japan [174]. His 97 word description in English was based on observations of many cells and was accompanied by two drawings. Cell size measurements were included. This species has been observed in Russian waters [36], in the Kara Sea [175] and in the Black Sea http://phyto.bss.ibss.org.ua/wiki/Gymnodinium_japonicum). This species has also been referred to as *G. japonica*.
135. ***Gymnodinium katodiniforme*** Elbrächter & Schnepf 1979* ***–*** This species was described by Elbrächter and Schnepf from an upwelling region north of Africa [176]. Their 449 word Latin and English description was based on observations of four individuals and included two drawings. Quantitative measurements of cell size were included in the text. This species has not been observed since its original description.
136. ***Gymnodinium klebsi*** **Lindemann 1928**– This species was originally described as *Hypnodinium sphaericum* Klebs 1912 from a swamp in Germany [37]. Klebs gave cell measurements and eight drawings in his German description. The species was then transferred to *Gymnodinium* by Lindemann and since *Gymnodinium sphaericum* was already occupied (*Gymnodinium sphaericum* (Calkins) Kofoid & Swezy 1921) he named it *Gymnodinium klebsi* Lindemann 1928 [99]. He did this without reporting a new observation or giving a species description. All information about this species that is available online is attached to the name *Hypnodinium sphaericum*. This species has been observed from freshwater lakes in North America [177], [85], the Mediterranean Sea [178] and the Black Sea [76]. All of these observations are reported as *Hypnodinium sphaericum* Klebs.
137. ***Gymnodinium knollii*** Schiller 1957* ***–*** This species was described by Schiller from the freshwater Lake Neusiedl on the Austria/Hungary border [15]. His 89 word description in German was based on observations from many cells and included four line drawings. Some cell size measurements were included. This species has not been observed since its original description.
138. ***Gymnodinium kowalevskii*** **Pitzik 1967** ***–*** This species was described by Pitzik from the tropical Atlantic Ocean [179]. The 689 word description in Latin and Russian included seven drawings and cell size measurements. This species has been observed in the Indian Ocean [180]. Pitzik states that the type culture for this species is housed at the Institute for Biology of the Southern Seas, Ukraine, but the authors cannot confirm this. Reports of studies on this culture are contained in a thesis [181].
139. ***Gymnodinium kujavense*** Liebetanz 1925* – This species was described by Liebetanz from Poland [182]. His 74 word, Latin description included cell measurements and two drawings. This species has not been observed since its description.
140. ***Gymnodinium lachmanni*** Saville-Kent 1880/81* ***–*** This species was first recorded from Norwegian waters as a minute *Peridinium* [183]. They included two line drawings that were very different from each other. Saville-Kent named the species *G. lachmanni* and described it using 83 English words [117]. This species has not been seen since Claparède and Lachmann, so its status as a real species is suspect [19].
141. ***Gymnodinium lackeyi*** (Lackey) Kiselev 1954* ***–*** This species was originally described from freshwater lakes in the USA as *G. limneticum* [17]. Kiselev renamed it *G. lackeyi* with no new observations [184]. His 68 word, Russian, description contained two drawings. It can be found in Popofsky and Pfiester with the Lackey drawing, but this species has not been reported since its original description [17], [12]. Care should be taken to distinguish *G. limneticum* Woloszyńska which is a synonym of *G. uberrimum*.
142. ***Gymnodinium lacustre*** **Schiller 1933** ***–*** This species was originally described from Austrian ponds [21]. The 134 word description in German was based on observations of many individuals and included four line drawings and basic cell size measurements. Since then, this species has been observed at other locations in Europe [185], [15] in the Philippine Sea [186], Lake Tanganyika (http://www.destin-tanganyika.com/Flore-Faune-Tanganyika/flore-faune-tanganyika-6.htm) and Japan [34]. Popovsky and Pfiester synonymized *Gymnodinium profundum* Schiller 1933 with this species [12]. This species has over 49 occurrence records in GBIF.
143. ***Gymnodinium lalitae*** Sarma & Shyam 1974* ***–*** This species was described by Sarma and Shyam from ponds in India [123]. Their 182 word description in English was based on observations of many cells and included seven images and cell size measurements. This species has not been reported since its initial description.
144. ***Gymnodinium lanskoi*** **Rouchijanen 1968** ***–*** This species was described by Rouchijanen from the Red Sea [187]. His 349 word, Latin and Russian description was based on observations of at least two cells, contained five drawings and several cell measurements. This species has also been observed in the Black Sea [188]. Rouchijanen claims that the type culture of this species is housed at the Institute for Biology of the Southern Seas, Ukraine, but the authors cannot confirm this.
145. ***Gymnodinium lantzschii*** **Utermöhl 1925** ***–*** This species was first described as *G. minimum* Lantzsch and then renamed *Glenodinium minimum* (Lantzsch) Bachmann [189], [190]. These names were synonymized as *Gymnodinium lantzschii* by Utermöhl who gives a 125 word account in German with no new images [191]. This should not be confused with *G. minimum* Klebs. *G. minimum* Lantzsch and *Glenodinium minimum* (Lantzsch) Bachmann had not been observed again before being synonymized and placed under *G. lantzschii* Utermöhl. Popovsky and Pfiester add *G. albulum* Lindemann 1928, *G. lantzschii* var. *rhinophoron* Javornický 1957, *G. rhinophoron* (Javornický) Litvinenko 1977 and *G. macronucleum* Litvinenko 1963 as synonyms [12]. They showed seven drawings. This species has been reported in Europe and North America [18], [60], [192], [82], [86]. Reports of observations include cell measurements and habitat information. It has 55 occurrence records in GBIF.
146. ***Gymnodinium latum*** **Skuja 1948** ***–*** This species was described by Skuja from freshwater material collected in Sweden [193]. His 303 word, Latin and German description included four drawings which were reused in other works [185], [12]. Popovsky and Pfiester synonymize *G. alsiophyllum* Skuja 1964 with this species [12]. This species has been reported several times.
147. ***Gymnodinium lazulum*** **Hulburt 1957** ***–*** This species was described by Hulburt from brackish waters near Woods Hole area, Massachusetts, USA [77]. The 233 word description in English was based on observations of at least two cells and featured one drawing. The description included quantitative cell measurements. It has also been observed in the Chesapeake Bay, USA [47].
148. ***Gymnodinium legiconveniens*** Schiller 1957* ***–*** This species was described by Schiller from the freshwater Lake Neusiedl on the Austria/Hungary border [15]. His 110 word description in German was based on observations from at least two cells and includes three drawings. Cell size measurements were given. It has not been reported since.
149. ***Gymnodinium leptum*** Norris 1961* ***–*** This species was described by Norris from New Zealand waters [102]. The 195 word description in English was based on observations of at least two cells and contained one line drawing. Cell size measurements were included in the text. This species has not been reported since its description.
150. ***Gymnodinium limitatum*** **Skuja 1956** ***–*** This species was described from freshwater in Sweden [194]. His 26 word, Latin and German description contained three drawings. It has also been observed in a Polish freshwater lake [195] and in Japan [34]. There are a total of seven unique drawings available for this species.
151. ***Gymnodinium lineatum*** **Kofoid & Swezy 1921** ***–*** This species was described by Kofoid and Swezy from marine waters near La Jolla, California, USA [19]. The 712 word description in English contained two detailed drawings and was based on observations of one individual, even though two were seen. The description was very detailed and gave quantitative cell measurements. Schiller reported the species and gave a German description, but made no new observations [21]. This species has one occurrence record in GBIF. There are two drawings available to aid with identification and no photographs.
152. ***Gymnodinium lineopunicum*** Kofoid & Swezy 1921* ***–*** This species was described by Kofoid and Swezy from marine waters off La Jolla, California, USA [19]. Their 777 word description in English was accompanied by two detailed drawings, included quantitative cell measurements and was based on observations of one individual. Schiller gave an account in German, but made no new observations [21]. This species has not been reported since its description.
153. ***Gymnodinium lira*** **Kofoid & Swezy 1921** ***–*** This species was described by Kofoid and Swezy from material collected in marine waters near La Jolla, California, USA [19].Their 676 word, English description was based on observations of two cells and contained two detailed drawings and quantitative morphological measurements. Schiller gave a German description with no new observations [21]. It has been observed in the Mediterranean Sea [196]. One photograph is available online in addition to the two drawings in the original description.
154. ***Gymnodinium litoralis*** **Reñé 2011**– This species was thoroughly described by Reñé from marine waters in the mouth of the La Muga River, Spain [164]. The 1000+ word description in Latin and English was based on observations of many cells from a laboratory strain isolated from the Mediterranean Sea. Reñé thoroughly characterized the cell morphology, molecular sequences, phylogeny, pigments and ecology of this species. More than 20 images are available of this species showing cell morphology, ultrastructure and resting cysts. There are six sequences available in GenBank. The type material is available from the National Center for Marine Algae and Microbiota. This species has been observed throughout the western Mediterranean Sea and in Australian waters [164].
155. ***Gymnodinium lobularis*** **Campbell 1973** ***–*** This species was described by Campbell from the euryhaline portion of Gales Creek, North Carolina, USA [45]. His 198 word description in English was based on observations of at least eight cells and contained two line drawings. Cell size measurements were given. This species has also been reported from the Chesapeake Bay, USA [47].
156. ***Gymnodinium lucidum*** **Ballantine 1964** ***–*** This species was described by Ballantine [127]. Her 12 word description in English was based on an unknown number of cells and contained no images. Ballantine synonymized *Gymnodinium hyalinum* Lebour 1925 with this species. This species has also been reported from the Barents Sea (http://www.nodc.noaa.gov/OC5/BARPLANK/WWW/HTML/dino_p.html). As *G. hyalinum,* it has been reported from the Gulf of Mexico [56], the Atlantic Ocean [180]and the Aegean Sea [25].
157. ***Gymnodinium lunula*** **Schütt 1895** ***-*** This species was described by Schütt from the Atlantic Ocean [119]. His 344 word description in German included 12 drawings, but no text other than the figure captions which did not give cell measurements. This species has also been known as *Pyrocystis lunula* Schütt 1896, *Diplodinium lunula* Klebs 1912 and *Dissodinium lunula* Pascher 1916. It has been observed many times since its description and is considered to be cosmopolitan in marine waters [19]. There are 142 occurrence records for this species in GBIF. There are many drawings and photographs available in publications and on the internet.
158. ***Gymnodinium luteo-viride*** van Meel 1969* – This species was described by Van Meel from Belgian waters [97]. His 115 word description in French was based on observations of one cell and contained one line drawing and cell measurements. This species has not been reported since.
159. ***Gymnodinium maguelonnense*** Biecheler 1939* – Biecheler described this species from brackish water collected near the Maguelone Cathedral, France [197]. Her 569 word description in French was based on observations of many cells and contained one drawing. A strain of *Karenia selliformis* (GM94GAB) at the IFREMER culture collection was incorrectly known as *G. maguelonnense* [198], used in experiments under this name [199] and deposited to GenBank under this name (AF318225, now corrected). Unfortunately, several phylogenetic studies have included this sequence as *G. maguelonnense* [200], [201].
160. ***Gymnodinium mammosum*** van Meel 1969* – This species was described by Van Meel from Belgian waters [97]. His 45 word description in French was based on one cell and contained one drawing and cell measurements. It has not been seen since its original description.
161. ***Gymnodinium manchuriensis*** Thessen, Patterson & Murray 2012* – This species was described by Skvortzov from Northern Manchuria, China as *Gymnodinium autumnale* Skvortzov 1968 [43]. As this name was preoccupied by *Gymnodinium autumnale* Christen 1959, we have (below) re-named this species. Skvortzov’s 109 word, Latin and English description included cell measurements and one drawing. This species has not been observed since its description.
162. ***Gymnodinium marinum*** **Saville-Kent 1880/81**– Saville-Kent described this species from an infusion of hay in seawater from St. Heliers, Jersey, UK [117]. His 193 word description in English was based on observations of many cells and featured two drawings. He uncertainly claims that *G. marinum* is identical to *Peridinium monas* Ehrenberg 1840. This species was reported numerous times in multiple languages [19], [202], [203], [20], [21], [97]. It was not observed again until 1928, when Schiller observed it in the Adriatic Sea [48]. Then it was reported near Australia [95], Japan [161], in the North Atlantic [24], North African coastal waters [132], the Gulf of Mexico [56] and the Chesapeake Bay, USA [65]. This species has 157 occurrence records in GBIF.
163. ***Gymnodinium marylandicum*** Thompson 1947* – Thompson described this species from freshwater underneath ice near Belcamp, Maryland, USA [204]. The 142 word description in Latin and English was based on the observation of many cells and contained three drawings. Measurements of the cells and their cysts were given. This species has not been observed since.
164. ***Gymnodinium massarti*** (Conrad) Schiller 1933* – This species was described by Schiller and synonymized with *Ceratodinium asymmetricum* Conrad [205], [21]. Cell measurements were given in the 180 word description in German accompanied by one drawing. It has not been observed since its original description.
165. ***Gymnodinium maximum*** Nordli 1951* – Nordli described this species from material collected near the Lofoten Islands, Norway [206]. His 39 word description in English was accompanied by two drawings and was based on observations of one cell. This species has not been observed since its original description.
166. ***Gymnodinium meervalli*** **Redeke 1919**– This species was described by Redeke from artificial lakes in the Netherlands [207]. His 766 word description in Dutch included cell measurements, habitat information and two drawings. This species was observed by Redeke in two different lakes [207].
167. ***Gymnodinium microreticulatum*** **Bolch & Hallegraeff 1999**– Bolch and Hallegraeff described this species from cultures established from cysts collected from sediment in Australian waters [208]. Their Latin and English description was over 1000 words long, was based on observations from many cells and featured 18 photographs. The text was very descriptive, including quantitative measurements and characterizations of the molecular sequences, pigments and toxins. This species has been observed in Portugal [209] and in Australia [210]. There are seven sequences from this species in GenBank. A type culture is held at the University of Tasmania School of Plant Science Algal Culture Collection.
168. ***Gymnodinium minor*** **Lebour 1917**– This species was described by Lebour from Plymouth Sound, UK [16]. Her 60 word description in English was based on observations of at least two cells and featured two drawings. Only one cell measurement was given (length). This species has also been reported from Australia [95], Antarctica [108], [88], [133], the Pacific [102], Japan [161] and the Adriatic Sea [48]. This species has 20 occurrence records in GBIF. Several drawings are available that depict this species.
169. ***Gymnodinium minutulum*** Larsen 1994* – Larsen described this species from Australian marine waters [54]. His 274 word description in English was based on observations of more than 20 living cells and contained five images (one drawing and four photographs) and quantitative cell measurements. This species has not been observed since its description.
170. ***Gymnodinium mitratum*** **Schiller 1933**– This species was described by Schiller from material collected from the freshwater Lake Attersee, Austria [21]. His 131 word description in German was based on observations of at least four cells and contained three line drawings. Popovsky and Pfiester synonymized *G. eurytopum* Skuja 1948 and *G. simile* Skuja 1956 with this species [12]. This species has been observed in Czechoslovakia and Sweden [12], the Gulf of Mexico [56], the Mexican Pacific [75], the Mediterranean Sea [55], Romania [50], Poland (http://www.eko.org.pl/lkp/dpn/chckl_glony.html) and China [211].
171. ***Gymnodinium modestum*** **Balech 1976**– Balech described this species from Antarctica [88]. His Spanish and English description was 137 words long, based on observations of at least two cells and contained one drawing and cell size measurements. This species has been observed by others in Antarctica [212] and has one occurrence record in GBIF.
172. ***Gymnodinium multilineatum*** **Kofoid & Swezy 1921**– This species was described by Kofoid and Swezy from the marine waters near La Jolla, California, USA [19]. Their 635 word description in English was based on at least four cells and contained two detailed drawings and cell measurements. Schiller gave an account in German with no new observations [21]. It has been observed in the Mediterranean Sea [55].
173. ***Gymnodinium multistriatum*** **Kofoid & Swezy 1921**– This species was described by Kofoid and Swezy from the marine waters near La Jolla, California, USA [19]. Their 612 word description in English was based on observations of one cell and featured two drawings and cell measurements. Schiller published an account in German with no new observations [21]. This species was observed in Arctic Canada [58] the Gulf of Mexico [56] and Australian waters [95]. Five drawings and no photographs are available.
174. ***Gymnodinium myriopyrenoides*** **Yamaguchi, Nakayama, Kai & Inouye 2011**– This species was described from marine sands on Isonoura Beach, Japan [213]. Their lengthy English description contained information about the species morphology, ultrastructure and phylogeny and 23 photographs. This species has only been observed in Japan, but has been found in multiple samples collected over two years. Attempts to cultivate *G. myriopyrenoides* in the laboratory have not been successful, but a type specimen on a slide is available in the Department of Botany, National Museum of Nature and Science, Japan.
175. ***Gymnodinium najadeum*** **Schiller 1928**– Schiller described this species from the Adriatic Sea and the Gulf of Naples [48]. His 80 word description in Latin and German was based on observations of at least two cells and contained two drawings, cell measurements and a brief habitat description. This species has also been reported from the Ukraine [12]. There is one occurrence record in GBIF. Two drawings and no photographs are available.
176. ***Gymnodinium nanum*** **Schiller 1928**– This species was described by Schiller from the Adriatic Sea [48]. His 97 word description in Latin and German was based on observations of one cell and featured one drawing. Cell measurements and habitat information were given. This species has been reported from Australian waters [95], Spanish waters [51] and in the Gulf of Mexico [56]. The original drawing is the only image available to aid with identification.
177. ***Gymnodinium neapolitanum*** **Schiller 1928**– Schiller described this species from the Adriatic Sea [48]. His 214 word description was based on observations of many cells and contained two drawings. Cell measurements and habitat information was included. It has also been observed in Romania [50]. The two original drawings are the only images of this species available.
178. ***Gymnodinium nolleri*** **Ellegaard & Moestrup 1998**– Ellegaard and Moestrup described this species from Danish waters [214]. Their English-language description was well over 1000 words long and contained photographs and molecular information. This species has also been observed near Sweden [215]. There are 60 occurrences of this species in GBIF and five sequences in GenBank. A culture is available from the Scandinavian Culture Collection of Algae and Protozoa.
179. ***Gymnodinium nucaceum*** Okolodkov 1997* – Okolodkov described this species from the Greenland Sea [26]. His 295 word description in Latin and English was based on observations of one cell (which he measured) and was accompanied by one drawing. This species has not been seen since.
180. ***Gymnodinium obliquum*** Okolodkov 1997* – This species was described by Okolodkov from the Greenland Sea [26]. His 248 word description in Latin and English was based on one cell (measurements reported) and featured one drawing. This species has not been seen since its original description.
181. ***Gymnodinium oceanicum*** Hasle 1960* – This species was described by Hasle from the equatorial Pacific [30]. Her 189 word description in Latin and English was based on observations of at least two cells and featured three drawings. A range of length measurements was given. This species has not been seen since.
182. ***Gymnodinium ochraceum*** **Kofoid 1931**– Kofoid described this species from Mutsu Bay, Japan [67]. His 242 word description in English was based on observations of one cell and featured one drawing. Schiller gave an account in German with no new observations [21]. Wood observed this species in Australian waters [95]. This species has also been observed in the Gulf of Mexico [56]. Only two drawings are available to aid in identification of this species.
183. ***Gymnodinium octo*** **Larsen 1994**– Larsen described this species from Australian marine waters [54]. His 279 word description in Latin and English was based on observations of nine living cells and featured four images (one drawing and three photographs). Quantitative cell measurements were included in the text. Larsen also observed this species in Danish waters [54].
184. ***Gymnodinium olivaceum*** Skvortzov 1968* – This species was described by Skvortzov from Northern Manchuria, China [43]. His 88 word description in Latin and English included cell measurements and two drawings. This species has not been observed since its description.
185. ***Gymnodinium oppressum*** **Conrad 1926**– This species was described by Conrad from brackish water in ruins near Newport, UK [205]. His 342 word description in French featured six drawings. Schiller gave a German description with no new observations [21]. This species has also been observed in Belgian waters [18], British waters [60], the Mediterranean Sea [55] and the Black Sea (http://phyto.bss.ibss.org.ua/test/list.php).
186. ***Gymnodinium ostenfeldi*** **Schiller 1928**– Schiller described this species from the Adriatic Sea [48]. His 115 word description in Latin and German was based on at least two cells and contained two drawings. This species has been reported from the Seto Inland Sea, Japan [160], Danish waters [216], the Gulf of St. Lawrence [217] and Fram Strait [218]. All reports use the spelling *G. ostenfeldii*. This species has 18 occurrences in GBIF. Published photographs and drawings of this species are available.
187. ***Gymnodinium ovato-capitatum*** van Meel 1969* – This species was described by Van Meel from Belgian waters [97]. His 57 word description in French was based on one cell and accompanied by one drawing. The length and width of the cell was given. This species has not been seen since.
188. ***Gymnodinium ovoideum*** Okolodkov 1997* – This species was described by Okolodkov from the Norwegian Sea [26]. His 384 word, Latin and English description was based on one cell and accompanied by two drawings. Dimensions of the cell were given. It has not been observed since.
189. ***Gymnodinium ovulum*** **Kofoid & Swezy 1921**– This species was described by Kofoid and Swezy from the marine waters of La Jolla, California, USA [19]. Their 549 word description in English was based on the observation of many cells and featured two detailed drawings. Schiller gave an account in German with no new observations [21]. It has also been observed in the Mediterranean Sea [196].
190. ***Gymnodinium pachydermatum*** **Kofoid & Swezy 1921**– Kofoid and Swezy described this species in the marine waters off La Jolla, California, USA [19]. Their 680 word description in English was based on observations of three cells and contained two detailed drawings. They gave quantitative cell measurements and habitat information. Schiller gave an account in German with no new observations [21]. The drawings in Kofoid and Swezy and Schiller do not resemble each other [19], [21]. It has also been observed in the Gulf of Mexico [56].
191. ***Gymnodinium pallidum*** **Skuja 1939**– This species was described by Skuja from brackish water in the Gulf of Riga, Spain [219]. His 329 word description in Latin and German was based on observations from many cells and featured three drawings. Cell measurements and habitat information were given. This species has also been reported from British waters [60].
192. ***Gymnodinium palustriforme*** **Hansen & Flaim 2007**– This species was described by Hansen and Flaim from Lake Tovel, Italy [141]. Their 342 word description in Latin and English included cell measurements, four photographs and some habitat information. This species was observed at two separate times in Lake Tovel. A culture was established, but it is unknown if it has been deposited in a culture collection.
193. ***Gymnodinium paradoxiforme*** Schiller 1957* – This species was described by Schiller from freshwater near Vienna, Austria [15]. His 672 word description in German was based on observations of many cells and included seven drawings. Cell measurements were given. This species has not been reported since its initial description.
194. ***Gymnodinium paradoxum*** **Schilling 1891**– This species was described by Schilling from freshwater swamps near Basel, Switzerland [32]. His 121 word description in German was based on observations of one cell and contained one drawing and cell size measurements. This species has also been reported from the UK [220], [33], [105], [221], German ponds [222], [223], Romania [50], China [130], New Zealand [224] and France [225]. Popovsky and Pfiester synonymize *G. paradoxum* var. *maior* Lemmermann 1906 and *G. paradoxum* f. *astigmosa* Nygaard 1949 with this species [12]. This species has two occurrence records in GBIF. Several published drawings and photographs are available via the internet to aid in identification.
195. ***Gymnodinium parvum*** **Larsen 1994**– Larsen described this species from Australian marine waters [54]. His 266 word description in Latin and English was based on observations of 12 living cells and featured five images (four photographs and one drawing). It has also been observed in Belize [54] and the Gulf of St. Lawrence [217]. It has been misspelled in the abstract as *G. parvulum* [51 see abstract].
196. ***Gymnodinium patagonicum*** **Balech 1971**– Balech described this species from the Argentine shelf [226]. His 215 word description in Spanish was based on observations of at least three cells and featured two drawings and cell size measurements. This species has also been reported from the Black Sea (http://phyto.bss.ibss.org.ua/test/list.php).
197. ***Gymnodinium paulseni*** **Schiller 1928**– This species was described by Schiller from the Adriatic Sea [48]. His 86 word description in Latin and German was based on observations of at least two cells and contained two drawings and cell measurements. This species has also been reported from the Mediterranean Sea [55] and Canadian waters [227]. It has been misspelled as *G. paulsenii* [55], [14] and *G. paulseinii* (Catalogue of Life 2009, www.catalogueoflife.org, accessed April 23, 2012).
198. ***Gymnodinium pavlae*** Popovsky 1990* – This species was described by Popovsky from a freshwater swamp in Central Europe [86]. His 208 word, Latin and English description was based on at least two cells and contained two images. It has not been reported since its description.
199. ***Gymnodinium peisonis*** Schiller 1957* – This species was described by Schiller from the freshwater Lake Neusiedl on the Austria/Hungary border [15]. His 98 word description in German was based on observations from at least two cells and contained five drawings. Length and width measurements were given for the cells. This species has not been seen since its description.
200. ***Gymnodinium perplexum*** van Meel 1969* – Van Meel described this species from Belgian waters [97]. His 149 word description in French was based on observations from at least two cells and contained one drawing. A range of cell length and width measurements were given. This species has not been reported since.
201. ***Gymnodinium pingue*** van Meel 1969* – This species was described by Van Meel from Belgian waters [97]. His 60 word description in French was based on observations of one cell and contained two drawings. Cell length and height were given. This species has not been observed since its original description.
202. ***Gymnodinium placidum*** **Herdman 1922**– This species was described by Herdman from Port Erin, UK [228]. Her 153 word description in English was based on observations of many cells and contained one drawing and some cell measurements. This species has been observed in the Adriatic Sea [48] and from the sands at Port Erin, Isle of Man [20]. However, the cell size measurements given by Lebour [20] (32 µm length) and Herdman [228] (150 µm length) are very different and may not refer to the same species. There are five occurrence records in GBIF. Two drawings are available to aid in identification.
203. ***Gymnodinium planctonicum*** Skvortzov 1968* – This species was described by Skvortzov from Northern Manchuria, China [43]. His 95 word description in Latin and English included cell measurements and one drawing. This species has not been observed since its description.
204. ***Gymnodinium polycomma*** Larsen 1994* – Larsen described this species from Australian marine waters [54]. His 317 word descrption in Latin and English was based on observations of six living cells and contained five images (four photographs and one drawing). The text gave a thorough characterization of the morphology of the cell including measurements. This species has not been observed since its description.
205. ***Gymnodinium posthiemale*** Schiller 1957* – This species was described by Schiller from freshwater near Vienna, Austria [15]. His 117 word description in German was based on observations of at least two cells and contained four drawings. Cell size measurements were included. It has not been reported since.
206. ***Gymnodinium prolatum*** Larsen 1994* – This species was described by Larsen from Australian marine waters [54]. His 318 word description in Latin and English was based on observations from more than 20 living cells and featured six images (five photographs and one drawing) and cell size measurements. This species has not been reported since.
207. ***Gymnodiniu pseudomirabile*** **Hansen & Flaim 2007**– This species was described by Hansen and Flaim from Lake Tovel, Italy [141]. Their 345 word, Latin and English description included cell measurements, habitat information and seven photographs. A culture has been established, but it is unknown if it has been deposited in a culture collection.
208. ***Gymnodinium pulchrum*** **Schiller 1928**– This species was described by Schiller from the Adriatic Sea [48]. His 130 word description in Latin and German was based on observations of many cells and contained one drawing, cell measurements and habitat information. This species has been reported from the Black Sea (http://phyto.bss.ibss.org.ua/test/list.php) and the Mediterranean [55]. Only one published drawing is available to aid in identification.
209. ***Gymnodinium pumilum*** Larsen 1994* – This species was described by Larsen from several sites in Australian marine waters [54]. His 362 word description in Latin and English was based on observations from 20 living cells and contained four images (three photos and one drawing). It has not been observed since.
210. ***Gymnodinium punctatum*** **Pouchet 1887**– This species was described by Pouchet off the French Atlantic coast [152]. The description was based on observations of one cell and contained one drawing. This species was not adequately described by Pouchet and may be a zoospore of a larger species [19]. It has also been reported from Barnegat Bay, New Jersey, USA [134], British waters [60], the Mexican Pacific [75] and Australian waters [95]. There are 10 occurrence records in GBIF.
211. ***Gymnodinium puniceum*** **Kofoid & Swezy 1921**– This species was described by Kofoid and Swezy from the marine waters off La Jolla, California, USA [19]. Their 772 word description in English was based on observations of one cell and contained two detailed drawings, quantitative cell measurements and some habitat information. Schiller reported the species in German [21]. This species has been observed in British waters [60].
212. ***Gymnodinium purpureum*** **Skuja 1956**– This species was described by Skuja from Swedish waters [194]. His 563 word description in Latin and German was based on observations of at least two cells and contained six drawings and cell size measurements. This species has also been reported in US waters [229].
213. ***Gymnodinium pygmaeum*** **Lebour 1925**– Lebour described this species from the English Channel [20]. Her 67 word description in English was based on several cells, contained one drawing and gave only one cell length measurement. This species has also been reported from Belgian waters [18], Australian waters [95], Danish waters [216], the Gulf of St. Lawrence [217] and the Adriatic Sea [48]. This species has five occurrence records in GBIF and one sequence in GenBank. It is sometimes misspelled as *G. pigmaeum*. Several published drawings and photographs are available to aid in identification.
214. ***Gymnodinium pyrocystis*** Jörgensen 1912* – This species was described by Jörgensen from the North Sea [230]. His 652 word description was given in German. This initial report has been published several times [19]–[21], but the species has not been observed since its original description.
215. ***Gymnodinium radiatum*** **Kofoid & Swezy 1921**– This species was described by Kofoid and Swezy from the marine waters near La Jolla, California, USA [19]. Their description was based on observations of one individual and included one drawing and several quantitative cell measurements. This species has also been reported from the Black Sea [66] and the Mediterranean Sea [231].
216. ***Gymnodinium ravenescens*** **Kofoid & Swezy 1921**– Kofoid and Swezy described this species from the marine waters of La Jolla, California, USA [19]. Their 428 word description in English was based on one individual and included two detailed drawings with quantitative cell measurements. Schiller reported the species with no new observations [21]. This species was seen again in Californian waters [82] and in the Mediterranean Sea [55].
217. ***Gymnodinium regulare*** van Meel 1969* – Van Meel described this species from Belgian waters [97]. His 83 word description in French was based on at least two cells and contained one drawing. Cell size measurements were given. It has not been reported since.
218. ***Gymnodinium rete*** Schütt 1895* – This species was described by Schütt from the Atlantic Ocean [119]. His 24 word description in German did not give quantitative information, but one drawing was given. It has not been seen since its description. Kofoid and Swezy suggested that it was a mutilated cell nearing lysis [19].
219. ***Gymnodinium rhomboides*** **Schütt 1895**– This species was described by Schütt from the Atlantic Ocean [119]. His 37 word description in German included two drawings. No text was given for this species, but the two drawings had descriptive captions. No measurements or habitat information was given. This species has also been observed in the Skagerrak [232], the Mexican Pacific [75], Plymouth Sound, UK [16], in the waters off Normandy, France [157], Romania [50] and the Adriatic Sea [48]. This species has six occurrence records in GBIF. Published drawings and photographs are available.
220. ***Gymnodinium roseolum*** (Schmarda) Stein 1878* – This species was first described by Schmarda as *Glenodinium roseolum* from the Natron Sea in Egypt [169]. Stein changed it to *Gymnodinium roseolum* [233]. This species has also been referred to as *Peridinium roseolum* [234]. Neither Schmarda nor Stein described the species thoroughly [19]. This species has not been observed since its original description.
221. ***Gymnodinium roseostigma*** **Campbell 1973**– This species was described by Campbell from euryhaline waters in Gales Creek, North Carolina, USA [45]. His 178 word description in English was based on observations from many cells and included five drawings, cell measurements and some habitat information. This species has been observed in the Gulf of St. Lawrence [217] and in New Jersey, USA [80]. Published photographs and drawings are available.
222. ***Gymnodinium rotundatum*** Skvortzov 1968* - This species was described by Skvortzov from Northern Manchuria, China [43]. His 60 word description in Latin and English included cell measurements and two drawings. This species should not be confused with *G. rotundatum* Klebs 1912 which has been synonymized with *Gymnodinium uberrimum* (Allman) Kofoid & Swezy 1921. *G. rotundatum* Skvortzov has not been observed since its description.
223. ***Gymnodinium rubricauda*** Kofoid & Swezy 1921* – Kofoid and Swezy described this species from the marine waters off La Jolla, California, USA [19]. Their 812 word description in English was based on observations of many cells and included two detailed drawings. Quantitative cell morphology measurements were included. A German report was given by Schiller [21]. This species has not been seen since its original description.
224. ***Gymnodinium rubrocinctum*** **Lebour 1925**– This species was described by Lebour from Plymouth Sound, UK [20]. Her 146 word description in English was based on at least two cells and included two drawings and a length measurement. This species has also been reported from Danish waters [216]. Published drawings and photographs are available. This species has one occurrence record in GBIF.
225. ***Gymnodinium scaphium*** van Meel 1969* – This species was described by Van Meel from Belgian waters [97]. His 55 word description in French was based on observations of one cell and contained one drawing. Length and width measurements were given. It has not been observed since.
226. ***Gymnodinium schaefferi*** Morris 1937* – This species was described by Morris from the brackish waters of Cold Spring Harbor, New York, USA while forming a large, yellow-amber bloom [235]. His 293 word description in English was based on observations of many living cells and contained two drawings. Quantitative morphological cell measurements were given. This species has not been observed since its original description.
227. ***Gymnodinium schuettii*** Schiller 1957* – Schiller described this species from freshwater in Vienna, Austria [15]. His 114 word description in German was based on observations of at least two cells and contained five drawings. Some cell measurements were given. This species has not been seen since its original description.
228. ***Gymnodinium scopulosum*** **Kofoid & Swezy 1921**– This species was described by Kofoid and Swezy from marine waters off La Jolla, California, USA [19]. Their 594 word description in English was based on observations of two cells and contained two detailed drawings. Limited habitat information and extensive cell morphology measurements were given. Schiller reported the species in German [21]. It has been observed from Australian waters [95], the Gulf of Mexico [56], the Mediterranean Sea [55] and British waters [60]. Three published drawings are available.
229. ***Gymnodinium semidivisum*** **Schiller 1928**– This species was described by Schiller from the Adriatic Sea [48]. His 121 word, Latin and German description was based on observations of at least two cells and contained two drawings. Cell measurements were provided. This species was observed in the Black Sea [66].
230. ***Gymnodinium servatum*** Busch 1927* – This species was described by Busch from Antarctic waters [236]. He gave a 113 word description in German that included one drawing and was based on observations of one cell. The bulk of the description focused on the remarkable gelatinous coating around the cell and reasons the cell might have such a coating. The drawing does not bear the typical characteristics of the genus *Gymnodinium*. This species has not been observed since the original description.
231. ***Gymnodinium sinuatum*** Skvortzov 1968* – This species was described by Skvortzov from Northern Manchuria, China [43]. His 94 word description in Latin and English included cell measurements and one drawing. It has not been observed since its original description.
232. ***Gymnodinium situla*** **Kofoid & Swezy 1921**– This species was described by Kofoid and Swezy from the marine waters of La Jolla, California, USA [19]. Their 872 word description in English was based on observations of at least three cells and contained two detailed drawings. Cell measurements and some habitat information were given. Schiller gave a German description with no new observations [21]. This species has been observed in Australian waters [95], the Gulf of Mexico [56] and the Mediterranean Sea [55]. Three published drawings are available.
233. ***Gymnodinium soyai*** **Hada 1970**– This species was described by Hada from Antarctica [108]. His 153 word description in English and Spanish was based on observations of many cells and contained two drawings and cell size measurements. This species has also been observed by Balech in Antarctica [88] and has one occurrence record in GBIF.
234. ***Gymnodinium sphaericum*** **(Calkins) Kofoid & Swezy 1921**– This species was originally described as *Gymnodinium gracile* var. *sphaerica* from fresh and salt waters off the coast of Woods Hole, Massachusetts, USA [237]. He gave one drawing and one length, width measurment despite reporting the species as “common”. Kofoid and Swezy elevated its rank to species after observing it off the coast of La Jolla, California, USA [19]. They gave additional cell measurments, a 701 word description in English and two drawings. This species has been observed in Australian waters [95], the Black Sea [66], the Mediterranean Sea [55] and Romania [50]. Four published drawings are available.
235. ***Gymnodinium sphaeroideum*** **Kofoid 1931**– This species was described by Kofoid from Mutsu Bay, Japan [67]. His 275 word description in English was based on observations of three cells and contained one drawing. He gave cell measurements and habitat information. This species has 20 occurrence records in GBIF and has been reported from the Mediterranean Sea [231].
236. ***Gymnodinium steini*** **(Klebs) Lindemann 1928**– This species was originally described as *Cystodinium steinii* Klebs 1912 and was collected from a swamp in Germany [37]. His description in German included 11 drawings and cell measurements. Later the species was transferred to *Gymnodinium* by Lindemann who did not report any new field observations or give a description of the cell [99]. The vast majority of information about this species that is available on the internet is associated with the name *Cystodinium steinii*. This species has been observed in Srebarna Lake, Bulgaria [238] and North Deming Pond, Minnesota, USA [229]. Both observations are reported as *Cystodinium*.
237. ***Gymnodinium stellatum*** **Hulburt 1957**– This species was described by Hulburt from the Woods Hole area in Massachusetts, USA [77]. The location is given as Salt Pond, but the pond nearest to Woods Hole known by this name is approximately 50 miles away in Eastham, MA. It is unknown if this is the correct pond. Hulburt’s 221 word description in English was based on observations of at least three cells and contained three drawings and cell size measurments. This species has also been observed in New Jersey, USA [80], eastern Russian waters [36], the Black Sea [69], the Skagerrak-Kattegat (http://www.smhi.se/oceanografi/oce_info_data/plankton_checklist/dinoflagellate_distribution/dinodistribution.htm) and Gales Creek, North Carolina, USA [45]. Thirteen published drawings are available.
238. ***Gymnodinium submontanum*** Schiller 1957* – This species was described by Schiller from the freshwater Lake Neusiedl on the Austria/Hungary border [15]. His 86 word description in German was based on observations of at least two cells and contained zero images. He gave some cell measurements. Schiller synonymized *G. albulum* Lindemann [21] with this species [15]. It has not been observed since.
239. ***Gymnodinium subroseum*** **Campbell 1973**– Campbell described this species from the polyhaline portion of Gales Creek, North Carolina, USA [45]. His 177 word description in English was based on observations of at least 43 cells and contained three drawings. This species has also been reported from the Gulf of St. Lawrence [217], New Jersey [80] and the Chesapeake Bay [47]. Published drawings and photographs are available.
240. ***Gymnodinium subrufescens*** **Martin 1929**– This species was described by Martin from the brackish Delaware and Barnegat Bay, USA [134]. His 158 word description in English was based on observations of many cells and contained one drawing and cell size measurements. This species has also been observed in the Chesapeake Bay [47].
241. ***Gymnodinium suffuscum*** van Meel 1969* – This species was described by Van Meel from Belgian waters [97]. His 85 word description in French was based on observations of one cell and contained one drawing with cell size measurements. This species has not been observed since its description.
242. ***Gymnodinium sulcatum*** **Kofoid & Swezy 1921**– This species was described by Kofoid and Swezy from the marine waters off La Jolla, California, USA [19]. Their 624 word description in English was based on observations of one cell and contained two detailed drawings and cell measurements. Schiller reported the species in German [21]. This species has been observed from Australian waters [95], the Black Sea [69] and the Mediterranean Sea [231]. There are three published drawings available.
243. ***Gymnodinium telma*** van Meel 1969* – Van Meel described this species from Belgian waters [97]. His 181 word description in French was based on observations of one cell and contained one drawing with cell size measurements. This species has not been reported since.
244. ***Gymnodinium terrum*** Baumeister 1943* – Baumeister described this species from Eggenfelden, Germany [42]. His 114 word description in German was based on at least two cells and contained one drawing. Cell size measurements were given. This species has not been seen since its description.
245. ***Gymnodinium thomasi*** **Christen 1959**– This species was described by Christen from freshwater in Switzerland [239]. His 275 word description in German was based on observations of many cells and contained three images. No cell measurements were available in the original description, but they were given in later observations [240], [34]. This species has also been observed in Japan [34]. Four published drawings are available.
246. ***Gymnodinium tintinnicola*** Lohmann 1908* – This species was described by Lohmann as it was emerging from a tintinnid ciliate [241]. His 11 word description in German contained three drawings. This may be a zoospore of a parasitic species and not a species of *Gymnodinium* [19]. It has not been observed since its description.
247. ***Gymnodinium translucens*** **Kofoid & Swezy 1921**– This species was described by Kofoid and Swezy from the marine waters of La Jolla, California, USA [19]. Their 708 word description in English was based on observations of one cell and contained two detailed drawings, cell measurements and habitat information. Schiller (1933) reported this species in German with no new observations [21]. Campbell also described a species called *G. translucens* from the polyhaline portion of Gales Creek, North Carolina, USA [45]. His drawing does not match the drawings in Kofoid and Swezy’s description, we believe Campbell misidentified his taxon, and have created a new name for this species (see below). *G. translucens* Kofoid & Swezy has been observed in the Gulf of Mexico [56] and the Mediterranean Sea [196].
248. ***Gymnodinium trapeziforme*** **Attaran-Fariman & Bolch 2007**– Attaran-Fariman and Bolch described this species from the south coast of Iran [242]. Their 1000+ word, Latin and English description was based on observations of many cells and contained 20 images. A type culture is available at the University of Tasmania, School of Aquaculture Laboratories. One sequence is available in GenBank with the number EF192414.
249. ***Gymnodinium triangularis*** Lebour 1917* – This species was described by Lebour from Plymouth Sound, UK [16]. Her 46 word description in English was based on observations of one cell and contained two drawings. One length measurement was given. This species has not been seen since its original description. Kofoid and Swezy proposed that this is a malformed cell of another species [19].
250. ***Gymnodinium triceratium*** **Skuja 1939**– This species was described by Skuja from freshwater in Latvia [219]. His 225 word, Latin and German description was based on observations of at least two cells and contained four drawings. This species has been observed in Maryland, USA [204], Mountain Lake, Virginia, USA [243] and in a peat bog in the Czech Republic [38]. Popovsky and Pfiester synonymized *Gyrodinium asymmetricum* Woloszyńska 1936 and *Gymnodinium impar* Harris 1940 (observed in Reading, UK) with this species [12]. There are 15 drawings available and no photographs.
251. ***Gymnodinium uberrimum*** **(Allman) Kofoid & Swezy 1921**– This species was first described from Ireland as *Peridinium uberrima* by Allman [244]. Kofoid and Swezy synonymized *Melodinium uberrimum* Saville-Kent 1880–81, *Gymnodinium mirabile* var. *rufescens* Penard 1891, *Gymnodinium rufescens* Lemmermann 1900 and *Glenodinium uberrimum* Schilling 1913 with *Peridinium uberrima* Allman and transferred it to *Gymnodinium* [19]. They provided some cell size measurements without making new observations. Popovsky and Pfiester synonymized *Gymnodinium mirabile* Penard 1891, *G. limneticum* Woloszyńska 1935, *G. irregulare* Christen 1959, *G. bogoriense* Klebs 1912, *G. obesum* [21], *G. rotundatum* Klebs 1912, *G. poculiferum* Skuja 1956, *G. limitatum* Skuja 1956, *G. uberrimum* var. *rotundatum* Popovský 1968 and *Gyrodinium traunsteineri* Lindemann 1928 with *G. uberrimum* (Allman) Kofoid & Swezy [12]. However, this synonymy was rejected by Hansen and Flaim who report *Gymnodinium mirabile* Penard and *Gymnodinium obesum* Schiller from freshwater lakes in Italy [141]. This species has been reported from freshwater all over Europe [207], [60], [50], [192], [221], [86], [141], Japan [34], Australia [95], North America [17], [82], [75], Africa (http://www.destin-tanganyika.com/Flore-Faune-Tanganyika/flore-faune-tanganyika-6.htm) and India [245]. This species has 28 occurrence records in GBIF. This species has been misspelled as *Gymnodinium uberimum* and *Gymnodiniium uberrima*. Numerous drawings are available, especially under the synonymyzed names.
252. ***Gymnodinium uncatenum*** **(Hulburt) Hallegraeff 2002**– This species was originally described from Woods Hole, Massachusetts, USA and named *Gyrodinium uncatenum* Hulburt [77]. Hallegraeff transferred this species to *Gymnodinium* [246]. It has been observed in North America and Australian waters ([77], [45], [247], [248] http://www.dnr.state.md.us/bay/cblife/algae/dino/gyrodinium_uncatenum.html). Three sequences are available in GenBank under the name *Gyrodinium uncatenum*. Most of the information available online is connected to the older name.
253. ***Gymnodinium valdecompressum*** **Campbell 1973**– This species was described by Campbell from euryhaline portion of Gales Creek, North Carolina, USA [45]. His 197 word description in English was based on at least two cells and contained five drawings. Quantitative cell size measurements were given. This species was seen again in the Gulf of St. Lawrence [217] and in the Chesapeake Bay [47].
254. ***Gymnodinium variabile*** **Herdman 1924**– This species was described by Herdman from Port Erin, UK [165]. Her 149 word description in English was based on observations of many cells and contained 12 drawings. She gave a length range of 8 to 40 µm and admitted that some of the smaller cells might have been some other species. This species has been observed off the coast of France [70], the west coast of Europe [157], Cortes Island, Canada [58], San Diego, USA [82], the Chesapeake Bay, USA [47] and the Gulf of Mexico [56]. Later observations give additional photographs, drawings and measurements, helping to refine the species as 30–40 µm in length [58], [157], [70]. This species has nine occurrences in GBIF.
255. ***Gymnodinium varians*** **Maskell 1887**– Maskell described this species from New Zealand [249]. His 68 word description in English was based on observations from many cells and contained two drawings. He gave one length measurement. Kofoid and Swezy synonymized *Gymnodinium minimum* Klebs 1912 from freshwater in Java with this species [37], [19]. Their description goes into some additional morphological detail without making new observations. However, additional direct measurements were made later [185], [95], [38]. This species has also been reported from Australian waters [95], the Czech Republic [38], Spain [192], [51], Lake Tanganyika (http://www.destin-tanganyika.com/Flore-Faune-Tanganyika/flore-faune-tanganyika-6.htm) and the Netherlands [12]. This species has 17 occurrence records in GBIF. Nine published drawings are available. A strain is available from Canadian Center for the Culture of Microorganisms.
256. ***Gymnodinium vas*** van Meel 1969* – This species was described by Van Meel from Belgian waters [97]. His 68 word description in French was based on observations of one cell and contained one drawing with length and width measurements. This species has not been observed since.
257. ***Gymnodinium vastum*** Busch 1927* – This species was described by Busch from the Indian Ocean [236]. His 45 word description in German gave cell measurements and one drawing. This species has not been observed since its original description.
258. ***Gymnodinium venator*** **Flø Jørgensen & Murray 2004**– This species was described by Flø Jørgensen and Murray [250]. Their 32 word description in English was based on observations of many cells. They synonymized *Gymnodinium pellucidum* (Herdman) Flø Jørgensen & Murray, *Amphidinium pellucidum* Herdman 1922 and *Amphidinium subsalsum* Biecheler 1952 with this species. This species has also been reported from the UK [60], Kuwait [251] and Romania [50]. There are two sequences available in GenBank and three published photographs.
259. ***Gymnodinium vernale*** Skvortzov 1968* – This species was described by Skvortzov from Northern Manchuria, China [43]. His 112 word description in Latin and English included cell measurements and one drawing. This species has not been observed since its description.
260. ***Gymnodinium verruculosum*** **Cambell 1973**– Campbell described this species from the polyhaline portion of Gales Creek, North Carolina, USA [45]. His 163 word description in English was based on at least 43 cells and contained four drawings. He gave cell measurements and habitat information. This species has also been reported from New Jersey, USA [80], the Chesapeake Bay [47] and the Gulf of St. Lawrence [217]. Published drawings and photographs are available.
261. ***Gymnodinium vestifici*** **Schütt 1895**– This species was described by Schütt from the Atlantic Ocean [119]. He gave descriptive captions (30 German words) for two drawings. Lohmann observed it in the Baltic Sea and Ostenfeld observed it in the Kattegat [241], [252]. This species was reported by Kofoid and Swezy and Lebour with no new observations [19], [20]. Lebour stated that the species was not sufficiently described by Schütt [119]. It has been observed in the Mexican Pacific [75]. It has also been misspelled as *Gymnodinium vestificii*.
262. ***Gymnodinium violescens*** Kofoid & Swezy 1921* – This species was described by Kofoid and Swezy from the marine waters near La Jolla, California, USA [19]. Their 665 word description in English was based on observations of one cell and contained two detailed drawings. A detailed, quantitative description of the cell morphology was given. Schiller reported the species with no new observations [21]. This species has not been observed since its description.
263. ***Gymnodinium viridaliut*** Schiller 1957* – This species was described by Schiller from freshwater near Seewiesen, Austria [15]. His 158 word description in German was based on observations of many cells and contained three drawings. Cell measurements were included. This species has not been seen since.
264. ***Gymnodinium viridans*** van Meel 1969* – Van Meel described this species from Belgian waters [97]. His 80 word description in French was based on observations of at least two cells and contained one drawing. Cell size measurements were included. This species has not been reported since.
265. ***Gymnodinium viridescens*** **Kofoid 1931**– This species was described by Kofoid from Mutsu Bay, Japan [67]. His 261 word description in English was based on observations of one cell and contained one drawing. Cell measurements and habitat information was given. Schiller reported this species in German [21]. This species has also been observed in Hong Kong (http://www.epd.gov.hk/epd/english/environmentinhk/water/marine_quality/files/m01_c14.pdf), Xiamen, China [253] and the Mexican Pacific [75].
266. ***Gymnodinium voukii*** Schiller 1928* – This species was described by Schiller from the Adriatic Sea [21]. His 159 word description in German was based on many cells and contained one drawing. Cell measurements and habitat information were included. This species has not been observed since its description.
267. ***Gymnodinium wawrikae*** **Schiller 1957**– This species was described by Schiller from the freshwater Lake Neusiedl on the Austria/Hungary border [15]. His 72 word description in German was based on observations of at least two cells and contained two drawings and cell measurements. This species was also reported from Japan [34] and Ohio, USA [85]. Eight published drawings are available.
268. ***Gymnodinium wilczeki*** **Pouchet 1894**– This species was described by Pouchet from the Arctic Ocean near Spitzbergen [254]. The 205 word description in French gave one drawing, but the proportions of the drawing do not match the measurments stated in the text [19]. This species has been reported by Kofoid and Swezy, Lebour and Schiller, but observed only one other time on the east coast of the USA [19]–[21], [62]. Only one drawing is available to aid with identification.
269. ***Gymnodinium wulffii*** **Schiller 1933**– This species was described by Schiller from the Barents Sea [21]. His 85 word description in German was based on observations of at least two cells and contained five drawings. This species has also been reported from the Bering and Chuckchi Seas [255] and eastern Russian waters [36]. This species has over 500 occurrence records in GBIF. Published drawings and photographs are available.
270. ***Gymnodinium zachariasi*** **Lemmermann 1900**– This species was described by Lemmermann from a German freshwater Lake [256]. His three word description in German was “Verbreitung: Europa (Deutschland)”. He synonymized *G. palustre* Schilling 1891 with this species. Schilling described *G. palustre* in German using 192 words and one drawing [32]. This species has also been observed in Hungary [257] Germany [258] and Ireland [259]. Strains are available as *G. palustre* from the Scandinavian Culture Collection of Algae and Protozoa. This species has been misspelled as *G. zachariasii*.

Our concept of 'oncers' overlaps with uniques and singletons [5]. 'Singletons' are those species known from a single specimen. 'Uniques' are species that have only been collected once, but this term is most usually used in the sense of sampling procedures [260]–[264], that is, is a measure of the abundance of a taxon. Both terms are ambiguous as they have both ecological and taxonomic connotations. We use 'oncer' exclusively in the taxonomic sense, being a species that was described based on material from a single collection event and no further new information has been added subsequent to the description. Previous estimates of the number of oncers from surveys of other taxa [5] are consistent with our analysis.

We encountered 643 unique *Gymnodinium* names, of which 265 (41%) represent extant species still recognized as members of this genus. Six new names are presented in this paper. The other names represent taxa that have been transferred to other genera, nomen nudum, erroneosly formed names or misspellings. Of the remaining species (including the three new names), 103 (38%) satisfy our definition of oncers. There are 36 names for extinct species of *Gymnodinium*, 15 of which are still within the genus (Appendix S3). We do not discuss extinct species.

If synonymies are not taken into account, the number of nominal taxais 327, of which 108 (33%) are oncers. The species *G. acidotum* Nygaard, *G. albulum* Lindemann, *G. bogoriense* Klebs, *G. caudatum* Prescott, *G. helveticum* Penard, *G. inversum* Nygaard, *G. limneticum* Woloszyńska, *G. luteofaba* Javornický, *G. mirabile* Penard, *G. palustre* Schilling, *G. skvortzowii* Schiller, *G. thompsonii* (Thompson) Kiselev, *G. undulatum* Woloszyńska and *G. viride* Penard are included as synonyms here, but are considered by others as accepted species. All but two of these names (*G. helveticum* Penard and *G. palustre* Schilling) were synonymized with other species by Popovsky and Pfiester [12]. They are not accepted by all dinoflagellate taxonomists [265]. Although we have accepted the Popovsky and Pfiester synonymies, we recognize their controversial nature and the likelihood that some or all will be rejected.

The genus *Gymnodinium* was originally described by Stein [233]. It underwent a major revision over 100 years later [7]. Daugbjerg et al. redefined the genus based on characters such as the apical loop and flagellar root [7]. These characters are not known for many species in *Gymnodinium* and we presume that some species will be shown not to meet the new criteria. The concept of *Gymnodinium* as presented in this survey is broader than that of Daugbjerg et al. [7].This continues a familiar taxonomic trend, illustrated with *G. pyrenoidosum* or *G. quadrilobatum*, in which species meet the criteria for inclusion in *Gymnodinium* initially, but later fall outside the evolving scope of the genus ([4], Appendix S4). Our ‘nominal’ approach takes no responsibility for taxonomic judgements but simply includes taxa that have been referred to *'Gymnodinium'* and have not been rendered into synonymy or moved to other genera The new concept of the genus does not affect our conclusions about the proportion of oncers across the dinoflagellates because no new observations or synonymies are presented. However, as oncers are investigated and moved out of the genus, the proportion of oncers within *Gymnodinium* may change.

The estimate that almost 40% of species are oncers is unexpectedly high. Lim et al. [5] suggest that the proportion of taxonomic uniques ( = oncers) is similar across a very broad taxonomic spectrum. For reasons given below, we attribute this number in *Gymnodinium* largely to poor quality species descriptions. ’Oncers’ are of concern because they inflate global species estimates.

There are many reasons why taxa may be observed and reported once. Some reasons relate to properties that are inherent within the organism (i.e. are intrinsic) while others may have little to do with their biology (are extrinsic). We discuss these in more detail below.

### Extrinsic Factors

#### 1. The number of organisms observed

Among the descriptions of the 103 oncers are some based on a single cell. Any description based on a small number of specimens will fail to represent the natural variation within the species, and may be observations of damaged or teratological specimens of a known species. With narrow sampling, the author may fail to recognize the organism observed as a previously described species, and may introduce a new taxon where that act is not appropriate. Enough specimens should be studied to give accurate knowledge of the intraspecific variation [4], but we concede that this is not always possible.

#### 2. Language

**Figure 1:**
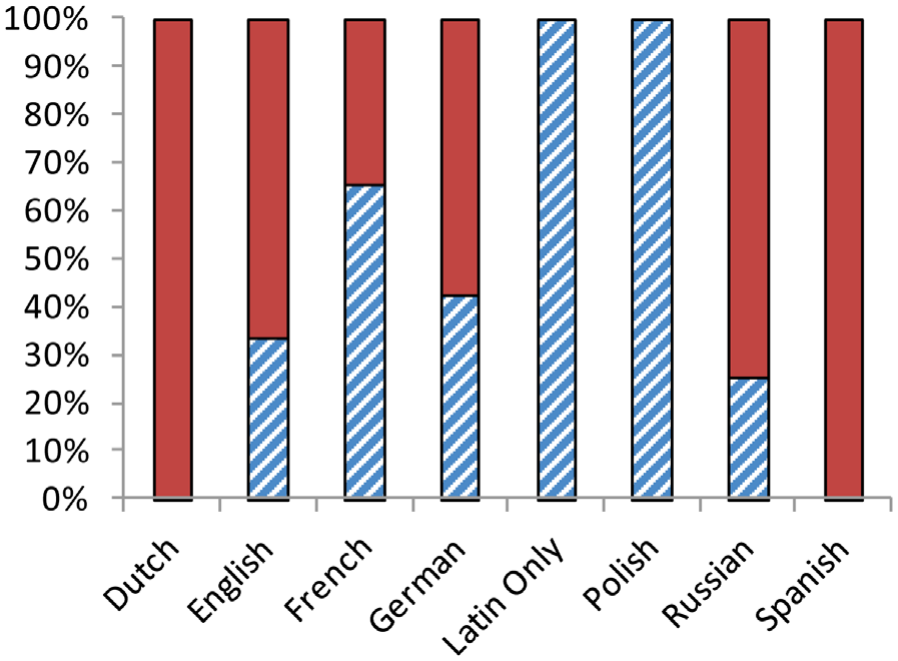
Proportion of oncers described in each language. The proportion of oncers in *Gymnodinium* originally described in each language is given in blue stripes.

**Figure 2:**
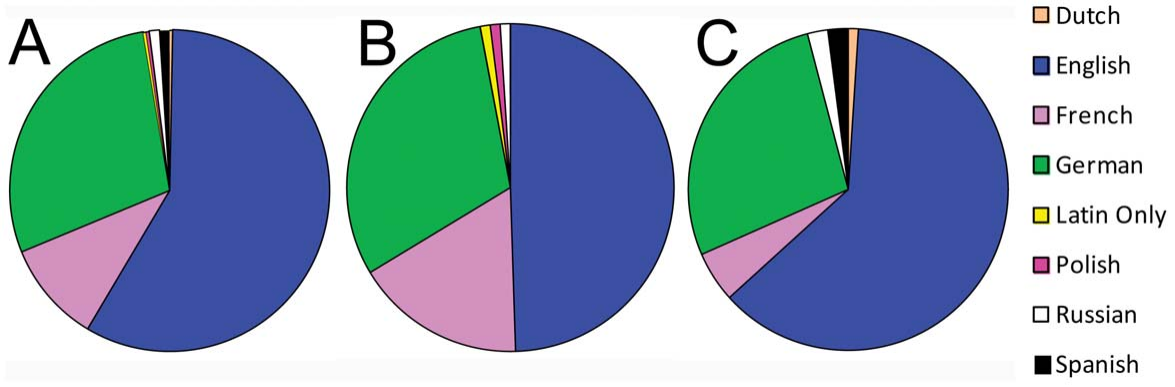
Proportion of *Gymnodinium* original species descriptions written in different languages. A) Proportion of original species descriptions in each language across the entire genus. B) Proportion of original species descriptions in each language for oncers. C) Proportion of original species descriptions in each language for species that have been observed since their original description.

The proportion of oncers differs among languages (Fig. 1) with two languages having no oncers (Dutch and Spanish) and two having only taxa that were oncers (Latin only and Polish). Of the species described in French, 65% are oncers (Fig. 1). Most descriptions of *Gymnodinium* are in English (Fig. 2A). Fewer oncers are described in English than are 'Seen Again' (50% vs 61%, Fig. 2B, C). A higher proportion of species described in English have been seen again (65%, Fig. 1). It seems reasonable to attribute this to English being the leading language of international scientific discourse [266], and that descriptions in other languages are less likely to be read or cited. Yet, species described in Russian, Dutch and Spanish have the highest percentage of “Seen Again” (Fig. 1). Whatever the cause, choice of language influences repeat observations.

#### 3. Length of description

Descriptions of species that have been seen only once are typically shorter than descriptions of species that have been seen multiple times (Table 1). If the number of words is a token of the care with which the characters of the species are described or compared to others, then longer descriptions are more thorough.

**Table 1:** Length of original species description in words for oncers and species that have been observed in multiple samples.

Length (in words)		
	**Seen Once**	**Seen Again**
Minimum	4	3
Maximum	847	1000+
Mode	46	1000
Median	147	230
Mean	217.74	336.38

#### 4. Revisionary component of a description

Authors of new species believe that they have observed species that have not been previously recognized. It is expected that all new descriptions will have a revisionary component in which the new species is compared with all existing species in the genus [4]. The Code of Zoological Nomenclature explicitly requires this (Article 13.1 [267]), but not all species of *Gymnodinium* have been described under the zoological code. Most descriptions refer to few if any other species. Without such comparisons, the identity of the new taxa may not be clear, such that it will be hard to later confirm their existence. Nearly half (45%) of the oncers contained no reference to known species in their description. Of 'seen agains', a lesser proportion (35%) lacked any reference to another species suggesting that they were describved more thoughtfully. The average number of species referred to in descriptions of “Seen Again” taxa is 1.6 versus 1.0 for the oncers.

#### 5. Author

The author of the largest number of species that have been seen only once is Schiller (19% of oncers). Fifty-four percent of the *Gymnodinium* species that he has described have not been observed by anyone else [15], [48], [21]. Van Meel has authored 13% of the oncers, 93% of his species of *Gymnodinium* have not been observed by anyone else [97]. No-one has re-observed the species of Skvortzov (who described 8 species of *Gymnodinium*) [43] and Okolodkov (who described 5 species of *Gymnodinium*) [26], [64]. At the opposite end of the spectrum, only 20% of the species of *Gymnodinium* described by Kofoid and Swezy have not been seen by anyone else [19].

#### 6. Uninterpreted materials

**Figure 3:**
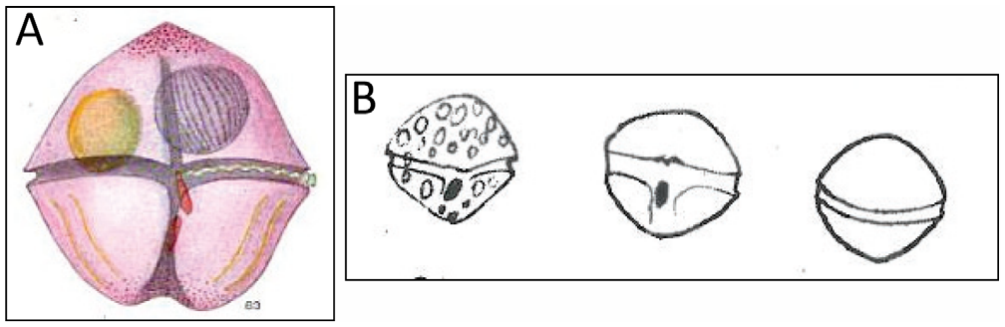
Drawings of *Gymnodinium* that accompany original descriptions. A) Drawing of *Gymnodinium sulcatum* Kofoid and Swezy 1921 [19]. B) Drawing of *Gymnodinium amphiconicoides* Schiller 1957 [15]. Items out of copyright. Note the difference in detail between the two drawings.

Many protists are hard to preserve and type material is often not available [268]. Under these circumstances, images become a valuable source of information [269]. Drawings are interpretations, can be inaccurate [270] and vary from very detailed to highly stylized (Fig. 3). Very good drawings often require observations of multiple cells, lots of time and a high degree of care. Photographs are uninterpreted records. Some protists, such as *Petalomonas boadicea,* have a photograph as the reference material for the type specimen [271]. Photographs are available for only 10% of the *Gymnodinium* oncers. The lack of uninterpreted images can contribute to uncertainty as to the identity of the taxon.

#### 7. Date of description

Table 2 suggests that species that have been known for a longer time are more likely to be re-reported than those described more recently, but the relationship is weak and this probably reflects the Author Effect (see #5) and the large number of species described by Schiller in 1957 [15] and Kofoid and Swezy in 1921 [19]. It makes logical sense that, as more time that passes after a species is known to science, the more likely it is to accumulate observations. This relationship is not clear from our data.

**Table 2:** Year in which *Gymnodinium* species were described for oncers and species that have been observed in multiple samples.

Year Described		
	**Seen Once**	**Seen Again**
Oldest	1878	1877
Youngest	1997	2011
Mode	1957	1921
Median	1957	1931

#### 8. Undersampling

Undersampling refers to techniques that intend to survey the diversity of organisms in habitats but that fail to report all species present. No study of natural habitats is expected to be comprehensive, but sampling protocols that involve small and occasional samples, samples that do not access microhabitats, all times of day or all yearly seasons are likely to under-report the species present and lead to more reports of oncers. Given that an array of communities have been subject to long term monitoring (such as at Helgoland, http://www.awi.de/en/research/research_divisions/biosciences/shelf_sea_ecology/long_term_studies/helgoland_roads_long_term_data_series/), undersampling will not be a universal issue. Not all reasons for taxa being reported only once are addressed by additional sampling [272].

#### 9. Skills and attitudes of observers

Non-taxonomists who are called upon to make species identifications from field samples may lack the skills or literature to appropriately discriminate among species [273]. Some species may be reported once because no one is looking for them. This is likely to bias reporting towards familiar taxa. This is compounded by a readiness to link observations to a species that does not quite fit rather than undertake the task of describing a new species [274], [275]. Such subjectivism is likely to lead to more records of species that are often referred to (such as *G. aeruginosum* or *G. fuscum* that appear in several algal identification guides), and will draw observations away from less familiar species. That is, these factors will increase the number of oncers. Researchers with a belief in cosmopolitanism will follow this trend, whereas those who assume a high degree of endemism are likely to assign taxa of uncertain identity to a new species [276]. Given the overall lack of taxonomic training and access of comprehensive guides to the genus, we suspect that the trends that favor repeat observations of familiar species will be greater.

#### 10. Technology

**Figure 4:**
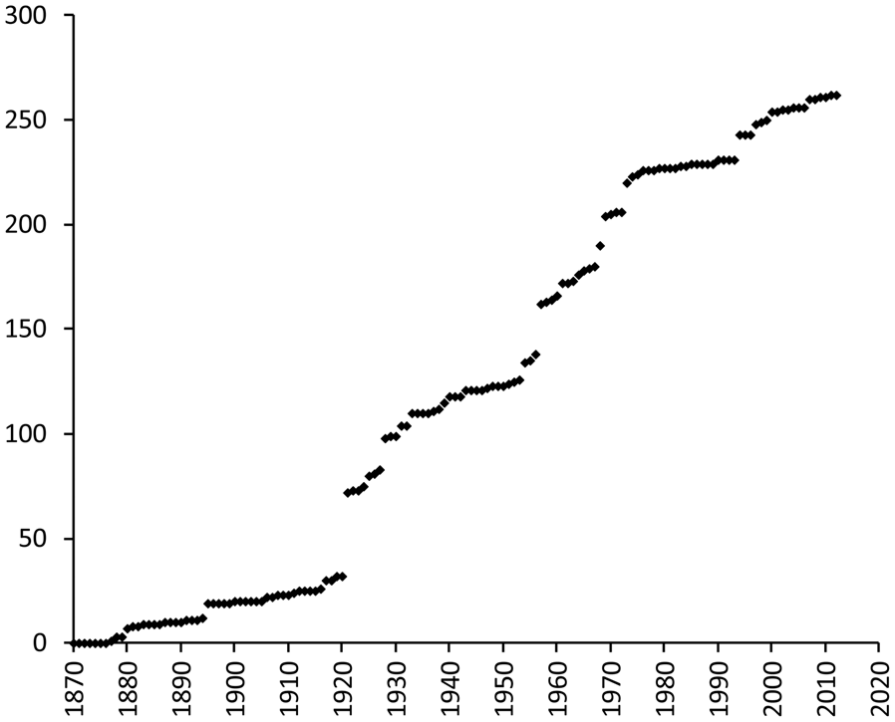
*Gymnodinium* species discovery curve. This figure shows the accumulated number of species of *Gymnodinium* known to science over time.

The application of newer technologies to the taxonomy of microbial eukaryotes [277], [278] leads to the description of new species distinguished by previously inaccessible characters. The discovery curve for *Gymnodinium* species (Fig. 4) shows a jump in new descriptions in the late 1950s and early 1960s, reflecting the intrusion of electron microscopy in protistan taxonomy [273], [279]. A smaller jump in the late 1990s may reflect the access to molecular information. Members of the *G. catenatum* Graham, *G. nolleri* Ellegaard & Moestrup and *G. microreticulatum* Bolch & Hallegraeff complex [103], [214], [208] are highly similar using light microscopy, but are clearly identifiable using genetic sequences and toxins [208]. *Gymnodinium nolleri* Ellegaard & Moestrup and *G. microreticulatum* Bolch & Hallegraeff were described in the 1990s.

### Intrinsic Factors

#### 1. Endemism

This refers to the occurrence of a taxon within a geographically constrained region. If species have a geographically restricted distribution, they are less likely to be re-encountered in later studies in different areas - that is, endemism will promote 'oncers'. It is difficult to assess endemism versus cosmopolitanism when faced with undersampling and poor taxonomic resolution [280]. The consensus for free-living protozoa is that the distribution is most usually cosmopolitan [276], [281], and in particular for flagellates [282]–[284]. Within *Gymnodinium*, many species (such as *Gymnodinium aeruginosa* Stein, *Gymnodinium fuscum* (Ehrenberg) Stein and *Gymnodinium uberrimum* (Allman) Kofoid & Swezy) occur over broad temporal and spatial scales.

**Figure 5:**
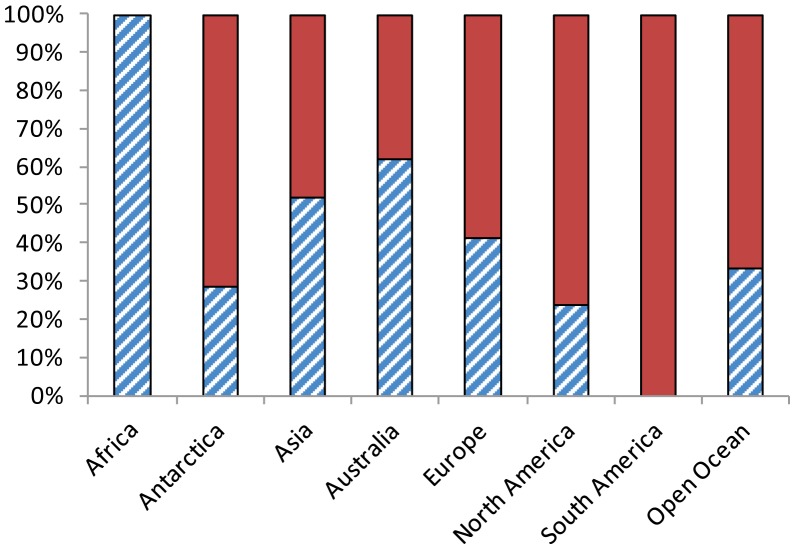
Proportion of species of *Gymnodinium* oncers described from each region. The proportion of oncers originally described from each region is given in blue stripes.

**Figure 6:**
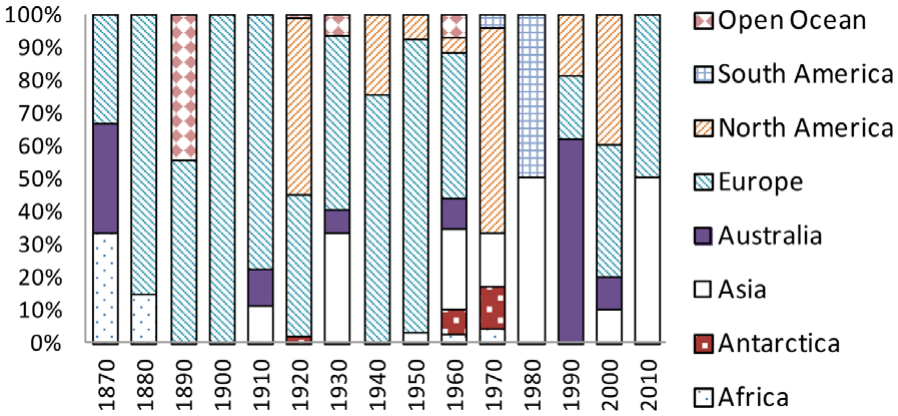
Proportion of original *Gymnodinium* descriptions from each region by decade.

No more than 13% of species of *Gymnodinium* have been described from Africa, Australia, South America and Antarctica together. Many species from Africa and Australia are oncers, but most from South America and Antarctica have been observed subsequently (Fig. 5). Increased sampling will erode arguments of endemism [285], and we note that Africa and Australia are undersampled (Fig. 6). Care must be applied, as the location of the taxonomist can have an effect on the assesment of biodiversity of a location; that is, areas with more taxonomists can appear to be more diverse [285].

*Gymnodinium baicalense* Antipova has so far been described from Lake Baikal, Russia [89]. Its morphology, molecular sequences and life history are well characterized. It has been observed numerous times in Lake Baikal, but not elsewhere. It may be endemic. Much of the literature on this species is in Russian and at the time of writing it is not included in AlgaeBase, extrinsic factors that make subsequent reporting less likely.

#### 2. Rarity

The concept of a rare biosphere refers to taxa that are present in very low numbers in ecosystems [6], a concept initially applied to prokaryotes but since extended to microbial eukaryotes [286], [287]. One suggested reason for rarity is highly selective niche preferences [288]. Rarity is not restricted to microscopic taxa [5]. Rarity will compound the favoring of oncers with undersampling. Some rarely reported yet distinctive protists may be examples of rare microbial eukaryote species. Examples are *Postgaardi mariagerensis*, *Chasmostoma nieuportense*, *Neobursaridium gigas* and *Amphidinium salinum* [289]–[294]. Interestingly these species may not be endemic to one region. This problem of undersampling may be more effectively addressed with the new high-throughput approach to sampling [287] than through traditional approaches.

#### 3. Damaged organisms

Observations made on a small number of cells may be of atypical cells, such as aberrant organisms or ones deformed through handling or disease. We believe this to be the most likely explanation for *G. massarti*, *G. rete* [19] and *G. triangularis* [19]. Molecular evidence may, in due course, clarify the status of these taxa.

### Our Thoughts as to the Cause of Oncers

The largest contribution to the number of oncers in the genus *Gymnodinium* appears to relate to extrinsic factors associated with the original descriptions. The association of particular authors (Schiller, van Meel, Okolodkov, and Skvortzkov) with oncers is striking. Such authors may describe taxa with uninformative brevity, make incomplete descriptions, rely on small numbers of taxa, provide no uninterpreted records or type material, fail to make comparisons with all other taxa in the genus, or observe damaged cells. The poor quality of the work of one of these authors has already required special action [295]. Poor descriptions ensure that taxa have uncertain or ambiguous identities, with the consequence that subsequent observations cannot be associated with the original description with confidence, or indeed require a massive revisionary effort [271]. The use of multiple codes of nomenclature (zoological and botanical) to describe *Gymnodinium* species adds to the confusion. Poorly described species are a familiar problem, but guidelines to address this cannot be applied retroactively [4]. Such an effort is now under way for another group of microscopic animals [296].

As observers are more likely to encounter common and widespread species, we can presume that the majority of the oncers described by these authors are of familiar species. That is, their oncers incorrectly inflate our estimate of species in the genus.

Additional factors that contribute to the number of oncers may be undersampling and rarity. Some oncers are described well and with uninterpreted materials [54] and reflect the continuing process of discovery within the undescribed parts of the biosphere.

We do not regard all oncers as being unsound. We offer a revised list of species within the genus *Gymnodinium* (Appendix S5), including species based on one or more of the following criteria.

1. The species has been observed on more than one occasion or in more than one place
2. The text description contains more than 500 words
3. More than one cell was observed to write the description
4. A laboratory strain is available
5. Molecular sequences are available
6. Photographs are available

This process eliminates some but not all of the ambiguous taxa. The taxa that are excluded by these criteria are listed as (Appendix S6).

**Figure 7:**
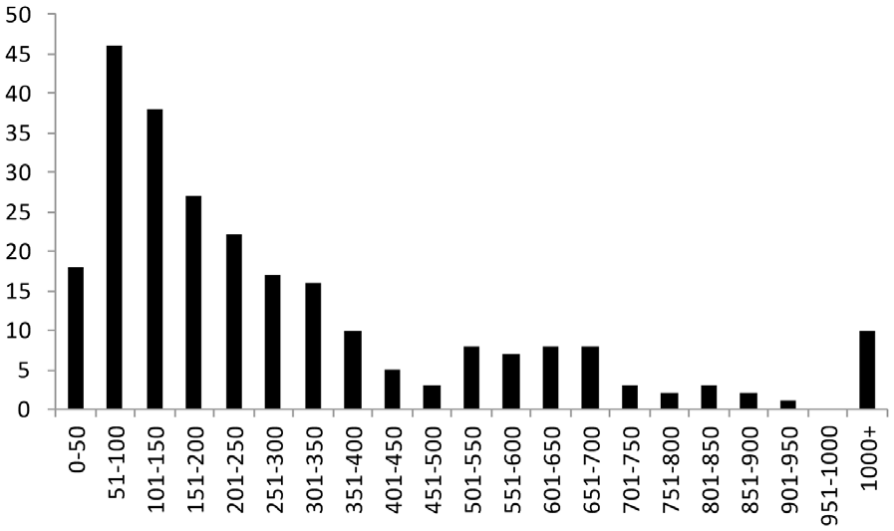
Distribution of the number of words used in species descriptions. This histogram show the number of species that have original descriptions of a given length divided into increments of 50. Note that the majority of species are described by less that 500 words.

Criterion number 2 is somewhat arbitrary and high (Fig. 7). Since only one of the criteria must be met in order for the species to be kept in Appendix S5, we wanted species that do not meet any of the other criteria to meet a rigorous text description standard. The 500 word requirement could be increased or decreased by 100 words before changing our result.

### Estimating Diversity and Number of Species

Some oncers result from poor descriptions that fail to provide taxa with clear identities. Some may be of species not described anywhere else, but most, we suspect, will be of taxa that had previously been or have subsequently been described under different names. We will never be sure of the identity of dubiously described taxa. Because of this, the current tally of known biodiversity [1] is not correct, but is an over estimate. In turn, that impacts estimates of the amount of biodiversity that has yet to be described [3].

This issue is not limited to *Gymnodinium*. Different approaches conclude that current estimates of (ciliate) biodiversity are excessive [281]. Dinoflagellate genera (such as *Prorocentrum*) have also undergone major downwards revisions [297] although the determinations are controversial [298]. A contributing factor for protists may be the relatively small number of available taxonomists. There are ten times more ornithologists (∼100,000) than species of bird (∼10,000; according to International Ornithological Congress, www.worldbirdnames.org), yet two orders of magnitude fewer diatomists (using the Diatom-L email listserve as a guide) than the estimated 100,000 diatom species. Yet, Lim et al. [5] found that 17.7% of invertebrate species and 19% of vertebrates were described from a single specimen (i.e. are singletons) and that the proportion of species described from a single location was 27.5% for invertebrates and 35% for vertebrates. That is, the larger number of taxonomists associated with vertebrates does not seem to affect the number of oncers. Lim noted that the proportion of singletons of vascular plants is lower (8%). By Lim et al.'s criteria, 12.4% of species of *Gymnodinium* are singletons. From our evaluation of the data on *Gymnodinium*, we conclude that between 10% and 25% of the species still currently assigned to the genus are not valid. This is consistent with other estimates of overdescription as being between 10 and 40% [5], [299]. This leads to overestimates of the biodiversity that has yet to be discovered [299].

### Can We Resolve Uncertainty with Molecular Analyses?

Molecular mechanisms that catalog biodiversity, especially for microbial eukaryotes [6], [286], [287], offer opportunities to clarify the diversity of species and to discriminate among species. The success of this approach to established taxa will depend on a reference system of sequences from as many known species as possible. Yet, only 7% of the taxonomically recognized species in *Gymnodinium* has a corresponding sequence in GenBank (Appendix S1). Very few species have been studied for variation around the species level [300]. Despite the investment in sequencing, this situation is not improving quickly. An increasing proportion of sequences deposited in GenBank do not have taxonomic names associated with them (http://iphylo.blogspot.com/2011/04/dark-taxa-genbank-in-post-taxonomic.html). As of 2011, only 5% of sequences from mammals had a species name, in 2007, only 30% of fungal sequences in the International Nucleotide Sequence Database had a species name [301]. There are 250 sequences in GenBank that referred to *Gymnodinium,* but only 86 (30%) are labeled with a proper species name. The proportion of the sequences that are incorrectly labeled is not known, and users are rarely provided with mechanisms to confirm identities. There is a clear need for closer engagement of traditional taxonomists and culture collections with these analyses. Under the present circumstances, any estimates of unknown diversity deriving from molecular studies are likely to be over-estimates.

### Digital Resources

As we move towards a digital data world [302], we are increasingly reliant on the internet as a source of information. This study has allowed us to assess resources available on the internet versus traditional print and word-of-mouth sources. We searched for original descriptions and nomenclatural acts using Google, Google Scholar and WorldCat. Thirty-one percent of publications had citations that were discoverable online and were digitally available to us online through a library subscription, Biodiversity Heritage Library, AlgaeBase or Google Books. A further 51% had discoverable citations but the content was not accessible to us on-line. As for the remaining 18%, they were not discoverable or obtainable through the internet. As a significant proportion of content is not freely available on-line, analyses that depend on the accessibility of content will be compromised [11]. Similarly, any study that relies only on traditional sources will not take advantage of information that is exclusively available via the internet. These include online species records, such as: Les algues, cyanobactéries et apparentés du lac Tanganyika (http://www.destin-tanganyika.com/Flore-Faune-Tanganyika/flore-faune-tanganyika-6.htm), B-NEAT (http://test.b-neat.org/home/), Nordic Microalgae (http://nordicmicroalgae.org/), Phytoplankton from the White Sea, Barents Sea, Norwegian Sea and Arctic Basin 1993–2003 (http://dw.sfos.uaf.edu/rest/metadata/ArcOD/2007P6), Algae noted in Drawa National Park, Poland (http://www.eko.org.pl/lkp/dpn/chckl_glony.html), micro*scope (http://starcentral.mbl.edu/microscope/portal.php) and the Black Sea Phytoplankton Checklist (http://phyto.bss.ibss.org.ua/test/list.php). All were used in this study, but maintaining awareness of on-line resources will become an increasing challenge for taxonomists.

Gathering species data online is hampered by some peculiarly unique biological problems. We rely heavily on species names to discover content, but that content may be labelled with any of the synonyms, and indeed the names may be spelled in sufficiently different ways as to make the content undiscoverable. A search using the name *Gymnodinium adriaticum* is unlikely to find content under *Heteroaulax adriatica* (Appendix S2). Much information on the same species may be attached to variant spellings or different names. To gather the data for Appendix S1, 413 individual searches were performed for the 265 nominal *Gymnodinium* species. Devices that will embed taxonomic knowledge within the internet and can manage problems associated with alternative names are now being developed [303]. That process is incomplete. The Biodiversity Heritage Library uses NameBank (http://www.ubio.org/index.php?pagename=namebank) as a reference system for indexing content. NameBank currently contains approximately 11 million names strings, 824 of which are of *Gymnodinium*. Yet these represent only 63% of the nominal species names in this manuscript (Appendix S5). When we searched BHL, 211 names returned no results. Half (51%) are likely to result from the absence of names in NameBank rather than the absence of content in BHL. An alternate list, the Global Names Index (gni.globalnames.org), holds approximately 17 million name strings, 1350 of which are names of *Gymnodinium*. This list is also likely to be incomplete. In order to improve the value of the internet as a scholarly data source, especially for taxonomic information, taxonomists will need to embed all names with all alternate forms into the infrastructure. This will improve the discovery of biological data [302].

### New Gymnodinium Names

We propose to eliminate the homonymy of *G. translucens* Campbell 1973 with *G. translucens* Kofoid & Swezy 1921, *G. autumnale* Skvortzov 1968 with *G. autumnale* Christen 1959, *G. irregulare* Christen 1959 and *G. irregulare* Conrad & Kufferath 1954 with *G. irregulare* Hope 1954, and *G. frigidum* Woloszynska 1952 with *G. frigidum* Balech 1965 and *G. frigidum* Skvortzov 1968 with the following new names:

1. *Gymnodinium campbelli* Thessen, Patterson and Murray nom. nov. Basionym: *Gymnodinium translucens*. Campbell 1973. Thesis Univ. North Carolina 143–144, pl. 7, fig 43.
2. *Gymnodinium antarcticum* Thessen, Patterson and Murray nom. nov. Basionym: *Gymnodinium frigidum*. Balech 1965. The Biology of Antarctic Seas II. Antarctic Research, Series 5∶112–114, pl. 1, fig 6–7.
3. *Gymnodinium chinensis* Thessen, Patterson and Murray nom. nov. Basionym: *Gymnodinium frigidum* Skvortzov 1968. Quarterly Journal of the Taiwan Museum 21∶87, pl. 2, fig 1.
4. *Gymnodinium manchuriensis* Thessen, Patterson and Murray nom. nov. Basionym: *Gymnodinium autumnale* Skvortzov 1968. Quarterly Journal of the Taiwan Museum 21∶88, pl. 2, fig 2.
5. *Gymnodinium christenum* Thessen, Patterson and Murray nom. nov. Basionym: *Gymnodinium irregulare* Christen 1959. Mitteilungen der Naturwissenschaftlichen Gesellschaft in Winterthur 29∶187, fig 6.
6. *Gymnodinium conkufferi* Thessen, Patterson and Murray nom. nov. Basionym: *Gymnodinium irregulare* Conrad & Kufferath 1954. Mémoires Institut Royal des Sciences Naturelles de Belgique 127∶97, pl. 2, fig 9.

### Conclusion

Over one third of the species of *Gymnodinium* have only been seen once. Using a number of criteria, 13% lack any clear identity. The status of these taxa is uncertain. The uncertainty is unsatisfactory but can be resolved through purposeful taxonomic revision. Similar proportions of uncertain taxa have been reported across all life. The figure of 1.9 million known living species is likely to be an overestimate, as are dependent estimates of the numbers of species to be discovered.

Authoritative statements about taxonomic issues must be attentive to all taxonomic and nomenclatural acts in over 250 years of literature. Traditional resources are becoming increasingly accessible through the internet, and new knowledge is appearing there without being replicated in traditional media. Yet, much digital content is not discoverable and/or is not accessible. A key to the issue of discoverability is to embed taxonomic knowledge, especially all names of all organisms, as a taxonomically intelligent component of the cyberinfrastructure upon which we will increasingly depend.

## Supporting Information

Appendix S1 Internet search results for each species of *Gymnodinium*. Websites searched were Biodiversity Heritage Library (BHL, www.biodiversitylibrary.org), Global Biodiversity Information Facility (GBIF, www.gbif.org), GenBank (www.ncbi.nlm.nih.gov/genbank/), Google Scholar (scholar.google.com) and the Web of Science (ISI, www.webofknowledge.com). Numbers indicate number of hits.
(DOCX)

Appendix S2 Names of species of *Gymnodinium* and their synonym groups.
(DOCX)

Appendix S3 Names associated with extinct species of *Gymnodinium* [304]–[308].
(DOCX)

Appendix S4 Names of *Gymnodinium* no longer associated with the genus [309]–[337]. The current name and/or the reason for rejecting the name is given. A name is listed as not code compliant if it is used without the existence of an original description. A name is listed as erroneous if it is an incorrect combination of genus name and species epithet.
(DOCX)

Appendix S5 List of species of *Gymnodinium* following removal of oncers that do not meet the selection criteria used here.
(DOCX)

Appendix S6 List of rejected *Gymnodinium* names.
(DOCX)
