# Supplementary material for: The Taxonomic Significance of Species That Have Only Been Observed Once: The Genus Gymnodinium (Dinoflagellata) as an Example
Source: PLoS One. 2012 Aug 30;7(8):e44015. doi: 10.1371/journal.pone.0044015 (PMC3431360; doi:10.1371/journal.pone.0044015)
Supplement: Appendix S1 — Internet search results for each species of Gymnodinium. Websites searched were Biodiversity Heritage Library (BHL, www.biodiversitylibrary.org), Global Biodiversity Information Facility (GBIF, www.gbif.org), GenBank (www.ncbi.nlm.nih.gov/genbank/), Google Scholar (scholar.google.com) and the Web of Science (ISI, www.webofknowledge.com). Numbers indicate number of hits. (DOCX) [file pone.0044015.s001.docx]

Appendix S1

| **Species Epithet** | **BHL** | **GBIF** | **GenBank** | **Google Scholar** | **ISI** |
| --- | --- | --- | --- | --- | --- |
| *absumens* | 0* | 0 | 0 | 1 | 0 |
| *achromaticum* | 2 | 2 | 0 | 19 | 0 |
| *achroum* | 0* | 0 | 0 | 1 | 0 |
| *acutiusculum* | 0* | 0 | 0 | 0 | 0 |
| *adriaticum* | 0 | 0 | 0 | 10 | 0 |
| *aequatoriale* | 0* | 0 | 0 | 2 | 0 |
| *aeruginosum* | 47 | 5 | 0 | 219 | 21 |
| *aesculum* | 0* | 0 | 0 | 0 | 0 |
| *aestivale* | 0* | 0 | 0 | 1 | 0 |
| *affine* | 0* | 0 | 0 | 0 | 0 |
| *agaricoides* | 0 | 0 | 0 | 2 | 0 |
| *agiliforme* | 0 | 281 | 0 | 23 | 0 |
| *alaskensis* | 0* | 0 | 0 | 2 | 0 |
| *allophron* | 0 | 0 | 0 | 2 | 1 |
| *amphiconicoides* | 0* | 0 | 0 | 0 | 0 |
| *amphityphlum* | 0 | 0 | 0 | 2 | 0 |
| *amphora* | 0* | 0 | 0 | 4 | 0 |
| *amplinucleum* | 0 | 0 | 0 | 1 | 0 |
| *antarcticum* | 0* | 0 | 0 | 0 | 0 |
| *arcticum* | 1 | 136 | 0 | 41 | 0 |
| *arcuatum* | 0 | 0 | 0 | 5 | 0 |
| *arenicolus* | 0 | 1 | 0 | 4 | 0 |
| *armoricanum* | 0* | 0 | 0 | 0 | 0 |
| *atomatum* | 0 | 0 | 0 | 1 | 0 |
| *attenuatum* | 0* | 0 | 0 | 3 | 0 |
| *aurantium* | 0 | 0 | 0 | 6 | 0 |
| *auratum* | 0* | 0 | 0 | 6 | 0 |
| *aureolum* | 2 | 397 | 16 | 1644 | 117 |
| *aureum* | 4 | 0 | 0 | 4 | 0 |
| *australe* | 1 | 0 | 0 | 0 | 0 |
| *australense* | 0 | 0 | 0 | 1 | 0 |
| *austriacum* | 0 | 0 | 0 | 13 | 1 |
|  |  |  |  |  |  |
| *baccatum* | 0 | 1 | 0 | 8 | 0 |
| *baicalense* | 0* | 0 | 5 | 24 | 1 |
| *baumeisteri* | 0* | 0 | 0 | 0 | 0 |
| *biciliatum* | 0* | 0 | 0 | 3 | 0 |
| *biconicum* | 0 | 14 | 0 | 7 | 0 |
| *bicorne* | 0* | 0 | 0 | 3 | 0 |
| *bifurcatum* | 0* | 0 | 0 | 3 | 0 |
| *bilobatum* | 0 | 1 | 0 | 2 | 0 |
| *birotundatum* | 0 | 1 | 0 | 1 | 0 |
| *bisaetosum* | 0* | 0 | 0 | 1 | 0 |
| *boguensis* | 0 | 1 | 0 | 2 | 0 |
| *bonaerense* | 0* | 0 | 0 | 2 | 0 |
| *caerulescens* | 0* | 0 | 0 | 1 | 0 |
| *campbelli* | 0* | 0 | 0 | 0 | 0 |
| *canus* | 0* | 0 | 0 | 4 | 0 |
| *capitatum* | 0 | 1 | 0 | 2 | 0 |
| *caput* | 0 | 0 | 0 | 5 | 0 |
| *cassiei* | 0* | 0 | 0 | 2 | 0 |
| *catenatum* | 23 | 122 | 102 | 2400 | 296 |
| *chiastosporum* | 0* | 3 | 0 | 2 | 0 |
| *chinensis* | 0* | 0 | 0 | 0 | 0 |
| *chukwanii* | 0* | 0 | 0 | 6 | 0 |
| *cinctum* | 3 | 0 | 0 | 5 | 0 |
| *cnecoides* | 0 | 1 | 0 | 17 | 0 |
| *cnodax* | 0 | 1 | 0 | 1 | 0 |
| *coeruleum* | 0 | 0 | 0 | 0 | 0 |
| *colymbeticum* | 0 | 1 | 0 | 0 | 0 |
| *concavum* | 0* | 0 | 0 | 1 | 0 |
| *conicum* | 2 | 20 | 0 | 32 | 1 |
| *contractum* | 0* | 0 | 0 | 2 | 0 |
| *corii* | 0 | 0 | 4 | 22 | 0 |
| *corollarium* | 0* | 0 | 1 | 2 | 2 |
| *corpusculum* | 0* | 0 | 0 | 0 | 0 |
| *costatum* | 2 | 0 | 0 | 9 | 0 |
| *cryophilum* | 0 | 0 | 0 | 4 | 0 |
| *cucumis* | 9 | 0 | 0 | 3 | 0 |
| *cyaneofungiforme* | 0 | 1 | 0 | 1 | 0 |
| *cyaneum* | 0 | 0 | 0 | 18 | 0 |
| *danicans* | 1 | 0 | 0 | 12 | 0 |
| *danubiense* | 0* | 0 | 0 | 1 | 0 |
| *deformabile* | 0 | 0 | 0 | 0 | 0 |
| *dentatum* | 0* | 0 | 0 | 1 | 0 |
| *depressum* | 0* | 0 | 0 | 0 | 0 |
| *devorans* | 0* | 0 | 0 | 0 | 0 |
| *diamphidium* | 0* | 0 | 0 | 1 | 0 |
| *diploconus* | 10 | 6 | 0 | 3 | 0 |
| *discoidale* | 0 | 2 | 0 | 2 | 0 |
| *dissimile* | 3 | 0 | 0 | 15 | 0 |
| *dodgei* | 0* | 0 | 0 | 2 | 0 |
| *dogieli* | 0* | 0 | 0 | 0 | 0 |
| *doma* | 0* | 0 | 0 | 2 | 0 |
| *dorsalisulcum* | 0* | 0 | 3 | 2 | 1 |
| *endofasciculum* | 0 | 1 | 0 | 5 | 0 |
| *enorme* | 0 | 1 | 0 | 1 | 0 |
| *eucyaneum* | 0 | 0 | 0 | 10 | 0 |
| *eufrigidum* | 0* | 0 | 0 | 1 | 0 |
| *excavatum* | 0 | 1 | 0 | 16 | 0 |
| *exechegloutum* | 0* | 0 | 0 | 1 | 0 |
| *filum* | 3 | 0 | 0 | 7 | 0 |
| *flavum* | 5 | 0 | 0 | 61 | 1 |
| *fossarum* | 0 | 0 | 0 | 1 | 0 |
| *frigidum* | 0 | 0 | 0 | 2 | 0 |
| *fukushimai* | 0* | 0 | 0 | 0 | 0 |
| *fulgens* | 0* | 0 | 0 | 2 | 0 |
| *fuscum* | 106 | 3 | 3 | 188 | 6 |
| *fusiforme* | 0* | 0 | 0 | 4 | 0 |
| *galeaeforme* | 0 | 15 | 0 | 7 | 0 |
| *galeatum* | 0 | 0 | 0 | 4 | 0 |
| *galeiforme* | 0* | 0 | 0 | 1 | 0 |
| *galesianum* | 0 | 1 | 0 | 4 | 0 |
| *gelbum* | 0 | 24 | 0 | 6 | 0 |
| *gibbera* | 0 | 0 | 0 | 4 | 0 |
| *glandiforme* | 0 | 1 | 0 | 1 | 0 |
| *glaucum* | 0 | 0 | 0 | 3 | 0 |
| *gleba* | 3 | 0 | 0 | 2 | 0 |
| *gracile* | 35 | 6 | 0 | 26 | 0 |
| *gracilentum* | 0 | 0 | 0 | 24 | 2 |
| *grammaticum* | 4 | 17 | 0 | 18 | 0 |
| *granii* | 0* | 0 | 0 | 1 | 0 |
| *guttiforme* | 0 | 0 | 0 | 1 | 0 |
| *guttula* | 0* | 0 | 0 | 3 | 0 |
| *hamulus* | 4 | 0 | 0 | 2 | 0 |
| *herbaceum* | 2 | 0 | 0 | 3 | 0 |
| *heterostriatum* | 3 | 81 | 0 | 37 | 1 |
| *hiemale* | 0 | 12 | 0 | 4 | 0 |
| *hiroshimaensis* | 0* | 0 | 0 | 1 | 0 |
| *huber-pestalozzii* | 0* | 0 | 0 | 1 | 0 |
| *hulburtii* | 0 | 0 | 0 | 2 | 0 |
| *impatiens* | 0 | 0 | 2 | 2 | 0 |
| *impudicum* | 0 | 0 | 8 | 78 | 10 |
| *incertum* | 0 | 1 | 0 | 12 | 0 |
| *incisum* | 0* | 0 | 0 | 3 | 0 |
| *incoloratum* | 0 | 0 | 0 | 7 | 0 |
| *inconstans* | 0 | 0 | 0 | 2 | 0 |
| *indicum* | 0* | 0 | 0 | 3 | 1 |
| *inerme* | 0* | 0 | 0 | 0 | 0 |
| *instriatum* | 0 | 0 | 0 | 171 | 6 |
| *intercalaris* | 0* | 0 | 0 | 2 | 0 |
| *irregulare* | 0 | 27 | 0 | 3 | 0 |
| *japonicum* | 0* | 0 | 0 | 6 | 0 |
| *katodiniforme* | 0 | 0 | 0 | 1 | 0 |
| *klebsi* | 0 | 0 | 0 | 7 | 0 |
| *knollii* | 0* | 0 | 0 | 1 | 0 |
| *kowalevskii* | 0* | 0 | 0 | 18 | 1 |
| *kujavense* | 0* | 0 | 0 | 0 | 0 |
| *lachmanni* | 0* | 0 | 0 | 3 | 0 |
| *lackeyi* | 0* | 28 | 0 | 9 | 0 |
| *lacustre* | 0 | 49 | 0 | 24 | 0 |
| *lalitae* | 0* | 0 | 0 | 2 | 0 |
| *lanskoi* | 0* | 0 | 0 | 2 | 0 |
| *lantzschii* | 1 | 55 | 0 | 218 | 1 |
| *latum* | 0 | 1 | 0 | 3 | 0 |
| *lazulum* | 4 | 0 | 0 | 2 | 0 |
| *legiconveniens* | 0* | 0 | 0 | 0 | 0 |
| *leptum* | 0* | 0 | 0 | 1 | 0 |
| *limitatum* | 0* | 0 | 0 | 4 | 0 |
| *lineatum* | 3 | 1 | 0 | 3 | 0 |
| *lineopunicum* | 0* | 0 | 0 | 2 | 0 |
| *lira* | 0* | 0 | 0 | 4 | 0 |
| *litoralis* | 0* | 0 | 6 | 1 | 1 |
| *lobularis* | 0 | 0 | 0 | 0 | 0 |
| *lucidum* | 0 | 0 | 0 | 0 | 0 |
| *lunula* | 157 | 142 | 38 | 996 | 51 |
| *luteo-viride* | 0* | 0 | 0 | 1 | 0 |
| *maguelonnense* | 0 | 0 | 0 | 17 | 1 |
| *mammosum* | 0 | 0 | 0 | 2 | 0 |
| *manchuriensis* | 0 | 0 | 0 | 1 | 0 |
| *marinum* | 15 | 157 | 0 | 23 | 0 |
| *marylandicum* | 0* | 0 | 0 | 2 | 0 |
| *massarti* | 0 | 0 | 0 | 1 | 0 |
| *maximum* | 0* | 0 | 0 | 2 | 0 |
| *meervalli* | 0* | 0 | 0 | 0 | 0 |
| *microreticulatum* | 0 | 0 | 7 | 44 | 5 |
| *minor* | 6 | 20 | 0 | 10 | 0 |
| *minutulum* | 0 | 0 | 0 | 1 | 0 |
| *mitratum* | 0 | 0 | 0 | 16 | 1 |
| *modestum* | 0 | 1 | 0 | 3 | 0 |
| *multilineatum* | 1 | 0 | 0 | 3 | 0 |
| *multistriatum* | 2 | 0 | 0 | 4 | 0 |
| *myriopyrenoides* | 0* | 0 | 0 | 3 | 1 |
| *najadeum* | 0 | 1 | 0 | 13 | 0 |
| *nanum* | 0 | 0 | 0 | 17 | 0 |
| *neapolitanum* | 0 | 0 | 0 | 4 | 0 |
| *nolleri* | 0 | 60 | 5 | 131 | 17 |
| *nucaceum* | 0* | 0 | 0 | 1 | 0 |
| *obliquum* | 0* | 0 | 0 | 1 | 0 |
| *oceanicum* | 0* | 0 | 0 | 1 | 0 |
| *ochraceum* | 2 | 1 | 0 | 3 | 0 |
| *octo* | 0 | 0 | 0 | 1 | 0 |
| *olivaceum* | 0 | 0 | 0 | 1 | 0 |
| *oppressum* | 0 | 0 | 0 | 3 | 0 |
| *ostenfeldi* | 0* | 18 | 0 | 4 | 0 |
| *ovato-capitatum* | 0* | 0 | 0 | 2 | 0 |
| *ovoideum* | 0* | 0 | 0 | 1 | 0 |
| *ovulum* | 0* | 0 | 0 | 13 | 0 |
| *pachydermatum* | 6 | 0 | 0 | 3 | 0 |
| *pallidum* | 0 | 0 | 0 | 1 | 0 |
| *palustriforme* | 0* | 0 | 0 | 1 | 0 |
| *paradoxiforme* | 0* | 0 | 0 | 1 | 0 |
| *paradoxum* | 13 | 2 | 0 | 34 | 1 |
| *parvum* | 1 | 0 | 0 | 1 | 0 |
| *patagonicum* | 0* | 0 | 0 | 2 | 0 |
| *paulseni* | 0* | 0 | 0 | 4 | 0 |
| *pavlae* | 0 | 0 | 0 | 0 | 0 |
| *peisonis* | 0* | 0 | 0 | 1 | 0 |
| *perplexum* | 0 | 0 | 0 | 2 | 0 |
| *pingue* | 1 | 0 | 0 | 2 | 0 |
| *placidum* | 0 | 5 | 0 | 7 | 0 |
| *planctonicum* | 0* | 0 | 0 | 1 | 0 |
| *polycomma* | 0 | 0 | 0 | 1 | 0 |
| *posthiemale* | 0* | 0 | 0 | 1 | 0 |
| *prolatum* | 0 | 0 | 0 | 1 | 0 |
| *pseudomirabile* | 0* | 0 | 0 | 1 | 0 |
| *pulchrum* | 0 | 0 | 0 | 4 | 0 |
| *pumilum* | 0 | 0 | 0 | 2 | 0 |
| *punctatum* | 7 | 10 | 0 | 24 | 0 |
| *puniceum* | 4 | 0 | 0 | 1 | 0 |
| *purpureum* | 0* | 0 | 0 | 2 | 0 |
| *pygmaeum* | 0 | 5 | 1 | 28 | 0 |
| *pyrocystis* | 2 | 0 | 0 | 6 | 0 |
| *radiatum* | 3 | 0 | 0 | 5 | 0 |
| *ravenescens* | 0* | 0 | 0 | 3 | 0 |
| *regulare* | 0 | 0 | 0 | 2 | 0 |
| *rete* | 0* | 0 | 0 | 1 | 0 |
| *rhomboides* | 19 | 6 | 0 | 28 | 0 |
| *roseolum* | 0* | 0 | 0 | 3 | 0 |
| *roseostigma* | 1 | 0 | 0 | 3 | 0 |
| *rotundatum* | 0 | 0 | 0 | 1 | 0 |
| *rubricauda* | 0* | 0 | 0 | 1 | 0 |
| *rubrocinctum* | 0 | 1 | 0 | 4 | 0 |
| *scaphium* | 0 | 0 | 0 | 2 | 0 |
| *schaefferi* | 0 | 0 | 0 | 3 | 0 |
| *schuettii* | 0* | 0 | 0 | 1 | 0 |
| *scopulosum* | 3 | 0 | 0 | 4 | 0 |
| *semidivisum* | 0 | 0 | 0 | 5 | 0 |
| *servatum* | 0* | 0 | 0 | 0 | 0 |
| *sinuatum* | 0 | 0 | 0 | 1 | 0 |
| *situla* | 2 | 0 | 0 | 3 | 0 |
| *soyai* | 0 | 1 | 0 | 3 | 0 |
| *sphaericum* | 4 | 0 | 0 | 3 | 0 |
| *sphaeroideum* | 0 | 20 | 0 | 5 | 0 |
| *steini* | 0 | 0 | 0 | 2 | 0 |
| *stellatum* | 5 | 0 | 0 | 23 | 0 |
| *submontanum* | 0* | 0 | 0 | 0 | 0 |
| *subroseum* | 1 | 0 | 0 | 3 | 0 |
| *subrufescens* | 1 | 0 | 0 | 1 | 0 |
| *suffuscum* | 0 | 0 | 0 | 2 | 0 |
| *sulcatum* | 7 | 0 | 0 | 5 | 0 |
| *telma* | 0 | 0 | 0 | 2 | 0 |
| *terrum* | 0* | 0 | 0 | 0 | 0 |
| *thomasi* | 0 | 0 | 0 | 0 | 0 |
| *tintinnicola* | 0* | 0 | 0 | 2 | 0 |
| *translucens* | 2 | 0 | 0 | 4 | 0 |
| *trapeziforme* | 0* | 0 | 1 | 5 | 1 |
| *triangularis* | 0* | 0 | 0 | 3 | 0 |
| *triceratium* | 1 | 0 | 0 | 9 | 0 |
| *uberrimum* | 34 | 28 | 0 | 237 | 6 |
| *uncatenum* | 0* | 1 | 3 | 240 | 24 |
| *valdecompressum* | 0 | 0 | 0 | 1 | 0 |
| *variabile* | 1 | 9 | 0 | 27 | 0 |
| *varians* | 9 | 17 | 3 | 66 | 1 |
| *vas* | 0 | 0 | 0 | 2 | 0 |
| *vastum* | 0* | 0 | 0 | 0 | 0 |
| *venator* | 2 | 0 | 2 | 24 | 0 |
| *vernale* | 0* | 0 | 0 | 1 | 0 |
| *verruculosum* | 1 | 0 | 0 | 4 | 0 |
| *vestifici* | 0 | 0 | 0 | 8 | 0 |
| *violescens* | 0* | 0 | 0 | 2 | 0 |
| *viridaliut* | 0* | 0 | 0 | 0 | 0 |
| *viridans* | 0 | 0 | 0 | 2 | 0 |
| *viridescens* | 0 | 0 | 0 | 3 | 0 |
| *voukii* | 0 | 0 | 0 | 4 | 0 |
| *wawrikae* | 0 | 0 | 0 | 6 | 0 |
| *wilczeki* | 0* | 0 | 0 | 1 | 0 |
| *wulffii* | 2 | 583 | 0 | 15 | 0 |
| *zachariasi* | 0* | 0 | 1 | 47 | 0 |
