# Supplementary material for: The Taxonomic Significance of Species That Have Only Been Observed Once: The Genus Gymnodinium (Dinoflagellata) as an Example
Source: PLoS One. 2012 Aug 30;7(8):e44015. doi: 10.1371/journal.pone.0044015 (PMC3431360; doi:10.1371/journal.pone.0044015)
Supplement: Appendix S2 — Names of species of Gymnodinium and their synonym groups. (DOCX) [file pone.0044015.s002.docx]

Appendix S2

| ***Gymnodinium* names and their reconciliation groups** | | **Class** |
| --- | --- | --- |
| *G. adriaticum* (Schmarda) Kofoid & Swezy 1921 | |  |
|  | *Heteraulacus adriaticum* Diesing 1850 | Heterotypic Synonym |
|  | *Heteroaulax adriatica* Diesing 1886 | Heterotypic Synonym |
|  | *Peridinium adriaticum* Schmarda 1846 | Heterotypic Synonym |
| *G. aeruginosum* Stein 1883 | |  |
|  | *Gymnodinium acidotum* Nygaard 1949 | Heterotypic Synonym |
|  | *Gymnodinium aeruginosa* | Lexical Variant |
|  | *Gymnodinium areuginosum* Stein | Lexical Variant |
|  | *Gymnodinium campaniforme* Popovsky 1971 | Heterotypic Synonym |
|  | *Gymnodinium p.dohrni* Wawrik 1956 | Heterotypic Synonym |
|  | *Gymnodinium viride* Penard 1891 | Heterotypic Synonym |
| *G. agiliforme* Schiller 1928 | |  |
|  | *Gymnodinium agiliformis* Schiller | Lexical Variant |
| *G. alaskensis* Bursa 1963 | |  |
|  | *Gymnodinium alaskense* Bursa | Lexical Variant |
| *G. arenicolus* Dragesco 1965 | |  |
|  | *Gymnodinium arenicola* Dragesco 1965 | Lexical Variant |
|  | *Gymnodinium arenicolum* Dragesco 1965 | Lexical Variant |
| *G. aureolum* (Hulburt) Hansen 2000 | |  |
|  | *Gyrodinium aureolum* Hulburt 1957 | Homotypic Synonym |
| *G. australe* Playfair 1919 | |  |
|  | *Gymnodinium australe* var. *acutum* Playfair 1919 |  |
|  | *Gymnodinium fuscum* var. *cornifax* (Schilling) Playfair 1917 | Heterotypic Synonym |
| *G. austriacum* Schiller 1933 | |  |
|  | *Gymnodinium autumnale* Christen 1959 | Heterotypic Synonym |
|  | *Gymnodinium cruciatum* Thompson 1950 | Heterotypic Synonym |
|  | *Gymnodinium thompsonii* (Thompson) Kiselev 1954 | Heterotypic Synonym |
|  | *Gymnodinium titubans* Christen 1958 | Heterotypic Synonym |
|  | *Gymnodinium tituhans* | Lexical Variant |
|  | *Gymnodinium tridentatum* Schiller 1933 | Heterotypic Synonym |
|  | *Gymnodinium waltzi* Baumeister 1957 | Heterotypic Synonym |
|  | *Gymnodinium waltzii* Baumeister 1957 | Heterotypic Synonym |
| *G. boguensis* Campbell 1973 | |  |
|  | *Gymnodinium boguense* | Lexical Variant |
| *G. cassiei* Norris 1961 | |  |
|  | *Gymnodinium caesiei* | Lexical Variant |
| *G. catenatum* Graham 1943 | |  |
|  | *Gymnodinium catena* | Lexical Variant |
|  | *Gymnodinium catenata* | Lexical Variant |
| *G. chiastosporum* (Harris) Cridland 1958 | |  |
|  | *Dinastridium chiastosporum* (Harris) Starmach 1974 | Homotypic Synonym |
|  | *Dinastridium sexangulare* Pascher 1927 | Heterotypic Synonym |
|  | *Gymnodinium chiastosporum* Elbrachter and Schnepf | Chresonym |
|  | *Gymnodinium hippocastanum* Cridland 1958 | Heterotypic Synonym |
|  | *Tetradinium chiastosporum* Harris 1940 | Homotypic Synonym |
| *G. cnecoides* Harris 1940 | |  |
|  | *Gymnodinium saginatum* Harris 1940 | Heterotypic Synonym |
|  | *Gymnodinium luteofaba* Javornický 1965 | Heterotypic Synonym |
| *G. coeruleum* Dogiel 1906 | |  |
|  | *Gymnodinium coerulatum* | Lexical Variant |
| *G. colymbeticum* Harris 1940 | |  |
|  | *Gymnodinium pulvisculcus* Klebs 1912 | Heterotypic Synonym |
| *G. conicum* Kofoid & Swezy 1921 | |  |
|  | *Gymnodinium viridis* Lebour 1917 | Heterotypic Synonym |
| *G. corpusculum* (Perty) Saville-Kent 1880/81 | |  |
|  | *Peridinium corpusculum* Perty 1852 | Homotypic Synonym |
| *G. cryophilum* (Wedemayer, Wilcox & Graham) Hansen & Moestrup 2000 | |  |
|  | *Amphidinium cryophilum* Wedemayer, Wilcox & Graham 1982 | Homotypic Synonym |
| *G. discoidale* Harris 1940 | |  |
|  | *Glenodinium eurystomum* Harris 1940 | Heterotypic Synonym |
|  | *Gymnodinium disoidale* | Lexical Variant |
| *G. dogieli* Kofoid & Swezy 1921 | |  |
|  | *Gymnodinium dogielii* | Lexical Variant |
| *G. dorsalisulcum* (Hulbert, McLaughlin & Zahl) Murray, de Salas & Hallegraeff 2007 | | |
|  | *Katodinium dorsalisulcum* Hulbert, McLaughlin & Zahl 1960 | Homotypic Synonym |
| *G. enorme* Ballantine 1964 | |  |
|  | *Gymnodinium irregulare* Conrad & Kufferath 1954 | Heterotypic Synonym |
|  | *Gymnodinium conkufferi* Thessen, Patterson & Murray 2012 | Heterotypic Synonym |
| *G. eucyaneum* Hu 1983 | |  |
|  | *Gymnodinium cyaneum* Hu 1980 | Heterotypic Synonym |
| *G. excavatum* van Meel 1969 | |  |
|  | *Gymnodinium excavata* | Lexical Variant |
| *G. fuscum* (Ehrenberg) Stein 1883 | |  |
|  | *Peridinium fuscum* Ehrenberg 1834 | Homotypic Synonym |
|  | *Gymnodinium caudatum*Prescott 1944 | Heterotypic Synonym |
|  | *Gymnocystodinium gessneri* Baumeister 1957 | Heterotypic Synonym |
|  | *Cystodinium gessneri* (Baumeister) Bourelly 1970 | Heterotypic Synonym |
| *G. fusiforme* Kofoid & Swezy 1921 | |  |
|  | *Spirodinium fusus* Meunier 1910 | Homotypic Synonym |
| *G. galeaeforme* Matzenauer 1933 | |  |
|  | *Gymnodinium galaeforme* | Lexical Variant |
| *G. gracile* Bergh 1881/82 | |  |
|  | *Gymnodinium abbreviatum* Kofoid & Swezy 1921 | Heterotypic Synonym |
|  | *Gymnodinium abbreviatum splendens* Lebour 1925 | Heterotypic Synonym |
|  | *Gymnodinium lohmanni* Paulsen 1908 | Lexical Variant |
|  | *Gymnodinium lohmannii* Paulsen 1908 | Heterotypic Synonym |
|  | *Gymnodinium roseum* Lohman 1908 | Lexical Variant |
|  | *Gymnodinium roseum* Lohmann 1908 | Heterotypic Synonym |
|  | *Gymnodinium spirale* var. *nobilis* Pouchet 1883 | Heterotypic Synonym |
| *G. grammaticum* (Pouchet) Kofoid & Swezy 1921 | |  |
|  | *Gymnodinium punctatum* var. *grammaticum* Poucet 1887 | Heterotypic Synonym |
| *G. guttula* (Hada) Balech 1976 | |  |
|  | *Gymnodinium cinctum* Hada 1970 | Heterotypic Synonym |
| *G. heterostriatum* Kofoid & Swezy 1921 | |  |
|  | *Gymnodinium spirale* var. *obtusum* Dogiel 1906 | Heterotypic Synonym |
| *G. hiemale* (Schiller) Popovsky | |  |
|  | *Katodinium hiemale* (Schiller) Loeblich 1965 | Homotypic Synonym |
|  | *Katodinium intermedium* Christen 1959 | Homotypic Synonym |
| *G. hiroshimaensis* Hada 1968 | |  |
|  | *Gymnodinium hiroshimaense* Hada | Lexical Variant |
| *G. huber-pestalozzii* Schiller 1957 | |  |
|  | *Gymnodinium austriacum* Schiller in Huber-Pestalozzi | Chresonym |
| *G. impudicum* (Fraga & Bravo) Hansen & Moestrup 2000 | |  |
|  | *Gyrodinium impudicum* Fraga & Bravo 1995 | Homotypic Synonym |
| *G. inerme* (Schmarda) Saville-Kent 1880/81 | |  |
|  | *Peridinium inerme* Schmarda 1854 | Homotypic Synonym |
| *G. instriatum* (Freudenthal & Lee) Coats 2002 | |  |
|  | *Gyrodinium instriatum* Freudenthal & Lee 1963 | Homotypic Synonym |
| *G. intercalaris* Bursa 1961 | |  |
|  | *Gymnodinium intercalare* | Lexical Variant |
| *G. japonicum* Hada 1974 | |  |
|  | *Gymnodinium japonica* Hada | Lexical Variant |
|  | *Gymnodinium japonica* var. *throndseni* Konovalova |  |
| *G. klebsi* Lindemann 1928 | |  |
|  | *Hypnodinium sphaericum* Klebs 1912 | Homotypic Synonym |
| *G. kowalevskii* Pitzik 1967 | |  |
|  | *Gymnodinium koyalevskii* | Lexical Variant |
| *G. lackeyi* (Lackey) Kiselev 1954 | |  |
|  | *Gymnodinium limneticum* Lackey 1936 | Heterotypic Synonym |
| *G. lacustre* Schiller 1933 | |  |
|  | *Gymnodinium profundum* Schiller 1933 | Heterotypic Synonym |
| *G. lantzschii* Utermöhl 1925 | |  |
|  | *Gymnodinium minimum* Lantzsch 1914 | Heterotypic Synonym |
|  | *Glenodinium minimum* (Lanzsch) Bachmann 1924 | Heterotypic Synonym |
|  | *Gymnodinium albulum* Lindemann 1928 | Heterotypic Synonym |
|  | *Gymnodinium alubulum* | Lexical Variant |
|  | *Gymnodinium lantzschii* var. *rhinophoron* Javornicky 1957 | Heterotypic Synonym |
|  | *Gymnodinium macronucleum* Litvinenko 1963 | Heterotypic Synonym |
|  | *Gymnodinium rhinophoron* (Javornicky) Litvinenko 1977 | Heterotypic Synonym |
| *G. latum* Skuja 1948 | |  |
|  | *Gymnodinium alsiophyllum* Skuja 1964 | Heterotypic Synonym |
| *G. lucidum* Ballantine 1964 | |  |
|  | *Gymnodinium hyalinum* Lebour 1925 | Heterotypic Synonym |
| *G. lunula* Schütt 1895 | |  |
|  | *Pyrocystic lunula* Schütt 1896 | Homotypic Synonym |
|  | *Diplodinium lunula* Klebs 1912 | Homotypic Synonym |
|  | *Dissodinium lunula* Pascher 1916 | Homotypic Synonym |
| *G. luteo-viride* Van Meel 1969 | |  |
|  | *Gymnodinium luteoviride* Van Meel | Lexical Variant |
| *G. marinum* Saville-Kent 1880/81 | |  |
|  | *Peridinium monas* Ehrenberg 1840 | Homotypic Synonym |
| *G. massarti* (Conrad) Schiller 1933 | |  |
|  | *Ceratodinium asymmetricum* Conrad 1926 | Homotypic Synonym |
|  | *Gymnofinium massartii* | Lexical Variant |
| *G. minor* Lebour 1917 | |  |
|  | *Gymnodinium minus* Lebour | Lexical Variant |
| *G. mitratum* Schiller 1933 | |  |
|  | *Gymnodinium eurytopum* Skuja 1948 | Heterotypic Synonym |
|  | *Gymnodinium simile* Skuja 1956 | Heterotypic Synonym |
| *G. modestum* Balech 1976 | |  |
|  | *Gymnodinium baccatum* Hada 1970 | Heterotypic Synonym |
| *G. nanum* Schiller 1928 | |  |
|  | *Gymnodinium nannum* | Lexical Variant |
| *G. oceanicum* Hasle 1960 | |  |
|  | *Gymnodinium aceanicum* | Lexical Variant |
| *G. oppressum* Conrad 1926 | |  |
|  | *Gymnodinium opressum* | Lexical Variant |
| *G. ostenfeldi* Schiller 1928 | |  |
|  | *Gymnodinium ostenfeldii* | Lexical Variant |
| *G. ovato-capitatum* van Meel 1969 | |  |
|  | *Gymnodinium ovatocapitatium* van Meel | Lexical Variant |
| *G. paradoxum* Schilling 1891 | |  |
|  | *Gymnodinium paradoxum* var. *maior* Lemmermann 1906 | Heterotypic Synonym |
|  | *Gymnodinium paradoxum* f. *astigmosa* Nygaard 1949 | Heterotypic Synonym |
| *G. parvum* Larsen 1994 | |  |
|  | *Gymnodinium parvulum* Larsen | Lexical Variant |
| *G. paulseni* Schiller 1928 | |  |
|  | *Gymnodinium paulsenii* | Lexical Variant |
|  | *Gymnodinium paulseinii* | Lexical Variant |
| *G. roseolum* (Schmarda) Stein 1878 | |  |
|  | *Glenodinium roseolum* Schmarda 1854 | Homotypic Synonym |
|  | *Peridinium roseolum* Maggi 1880 | Homotypic Synonym |
| *G. sphaericum* (Calkins) Kofoid & Swezy 1921 | |  |
|  | *Gymnodinium gracile* var. *sphaerica* Calkins 1902 | Homotypic Synonym |
| *G. steini* (Klebs) Lindemann | |  |
|  | *Cystodinium steinii* Klebs | Homotypic Synonym |
|  | *Cystodinium steini* | Lexical Variant |
| *G. submontanum* Schiller 1957 | |  |
|  | *Gymnodinium albulum* Lindemann in Schiller 1933 | Heterotypic Synonym |
| *G. thomasi* Christen 1959 | |  |
|  | *Gymnodinium thomasii* | Lexical Variant |
| *G. triangularis* Lebour 1917 | |  |
|  | *Gymnodinium triangulare* Lebour | Lexical Variant |
| *G. triceratium* Skuja 1939 | |  |
|  | *Gymnodinium impar* Harris 1939 | Heterotypic Synonym |
|  | *Gyrodinium asymmetricum* Woloszynska 1936 | Heterotypic Synonym |
| *G. uberrimum*(Allman) Kofoid & Swezy 1921 | |  |
|  | *Glenodinium uberrimum* Schilling 1913 | Homotypic Synonym |
|  | *Gymnodinium bogoriense*Klebs 1912 | Heterotypic Synonym |
|  | *Gymnodinium irregulare* Christen 1959 | Heterotypic Synonym |
|  | *Gymnodinium christenum* Thessen, Patterson & Murray 2012 | Heterotypic Synonym |
|  | *Gymnodinium limitatum* Skuja 1956 | Heterotypic Synonym |
|  | *Gymnodinium limneticum* Woloszynska 1935 | Heterotypic Synonym |
|  | *Gymnodinium mirabile* Penard 1891 | Heterotypic Synonym |
|  | *Gymnodinium mirabile* var. *rufescens* Penard 1891 | Heterotypic Synonym |
|  | *Gymnodinium obesum* Schiller 1933 | Heterotypic Synonym |
|  | *Gymnodinium poculiferum* Skuja 1956 | Heterotypic Synonym |
|  | *Gymnodinium rotundatum* Klebs 1912 | Heterotypic Synonym |
|  | *Gymnodinium rufescens* Lemmermann 1910 | Heterotypic Synonym |
|  | *Gymnodinium uberrimum* var. *rotundatum* Popovsky |  |
|  | *Gyrodinium traunsteineri* Lindemann 1928 | Heterotypic Synonym |
|  | *Melodinium uberrimum* Saville-Kent 1880/81 | Heterotypic Synonym |
|  | *Peridinium uberrima* Allman 1855 | Homotypic Synonym |
|  | *Gymnodinium ubberimum* | Lexical Variant |
| *G. uncatenum* (Hulburt) Hallegraeff | |  |
|  | *Gymnodinium uncatenatum* | Lexical Variant |
|  | *Gyrodinium uncatenum* Hulburt 1957 | Homotypic Synonym |
| *G. varians* Maskell 1877 | |  |
|  | *Gymnodinium minimum* Klebs 1912 | Heterotypic Synonym |
| *G. venator* Flø Jørgensen & Murray 2004 | |  |
|  | *Gymnodinium pellucidum* (Herdman) Flø Jørgensen & Murray 2004 | Homotypic Synonym |
|  | *Amphidinium pellucidum* Herdman 1922 | Homotypic Synonym |
|  | *Amphidinium subsalsum* Biecheler 1952 | Heterotypic Synonym |
| *G. vestifici* Schütt 1895 | |  |
|  | *Gymnodinium vestificii* | Lexical Variant |
| *G. wulffii* Schiller 1933 | |  |
|  | *Gymnodinium wulffi* | Lexical Variant |
|  | *Gymnodinium wulfii* | Lexical Variant |
| *G. zachariasi* Lemmermann 1900 | |  |
|  | *Gymnodinium palustre* Schilling 1891 | Heterotypic Synonym |
|  | *Gymnodinium zachariasii* | Lexical Variant |
