# Supplementary material for: The Taxonomic Significance of Species That Have Only Been Observed Once: The Genus Gymnodinium (Dinoflagellata) as an Example
Source: PLoS One. 2012 Aug 30;7(8):e44015. doi: 10.1371/journal.pone.0044015 (PMC3431360; doi:10.1371/journal.pone.0044015)
Supplement: Appendix S3 — Names associated with extinct species of Gymnodinium [304]–[308]. (DOCX) [file pone.0044015.s003.docx]

Appendix S3

| **Name** | **Current Name** | **Reference** |
| --- | --- | --- |
| *Gymnodinium albertii* Vozzhennikova | |  |
| *Gymnodinium attedalense* Cookson & Eisenack | |  |
|  | *Endoscrinium attadalense* (Cookson & Eisenack) Riding & Fensome | [304] |
| *Gymnodinium australiense* Deflandre & Cookson | |  |
|  | *Apteodinium australiense* (Deflandre & Cookson) Williams | [305] |
| *Gymnodinium avellana* Lejeune-Carpentier | |  |
|  | *Dinogymnium avellana* (Lejeune-Carpentier) Evitt, Clarke & Verdier | [306] |
| *Gymnodinium cretaceum* Deflandre | |  |
|  | *Dinogymnium cretaceum* (Deflandre) Evitt, Clarke & Verdier | [306] |
| *Gymnodinium cretaceum* var. *undulacostata* Boltenhagen | |  |
|  | *Dinogymnium cretaceum* var. *undulacostata* | [306] |
| *Gymnodinium curvatum* Vozzhennikova | |  |
| *Gymnodinium dabendorfense* Alberti | |  |
|  | *Luxadinium dabendorfense* (Alberti) Bujak & Davies | [307] |
| *Gymnodinium decorum* Deflandre | |  |
|  | *Dinogymnium decorum* (Deflandre) Evitt, Clarke & Verdier | [306] |
| G*ymnodinium denticulatum* Alberti | |  |
|  | *Dinogymnium denticulatum* (Alberti) Evitt, Clarke & Verdier | [306] |
| *Gymnodinium digitus* Deflandre | |  |
|  | *Dinogymnium digitus* (Deflandre) Evitt, Clarke & Verdier | [308] |
| *Gymnodinium digitus* var. *crassus* Vozzhennikova | |  |
| *Gymnodinium fehmarnense* Morgenroth | |  |
| *Gymnodinium gabonense* Deflandre | |  |
| *Gymnodinium galeritum* Delfandre | |  |
|  | *Endoscrinium galeritum* (Deflandre) Vozzhennikova | [304] |
| *Gymnodinium heterocostatum* Deflandre | |  |
|  | *Dinogymnium heterocostatum* (Deflandre) Evitt, Clarke & Verdier | [306] |
| *Gymnodinium heterocostatum* var. *kolpaschevi* Vozzhennikova | |  |
| *Gymnodinium hexagonum* Deflandre-Rigaud | |  |
|  | *Dinogymnium hexagonum* (Deflandre-Rigaud) Evitt, Clarke & Verdier | [306] |
| *Gymnodinium hyalinum* Vozzhennikova 1967 | |  |
| *Gymnodinium kasachstanicum* Vozzhennikova | |  |
| *Gymnodinium laticinctum* Deflandre | |  |
|  | *Dinogymnium laticinctum* (Deflandre) Evitt, Clarke & Verdier | [306] |
| *Gymnodinium longicorne* Vozzhennikova | |  |
| *Gymnodinium longicornis* Vozzhennikova | |  |
| *Gymnodinium marthae* Deflandre | |  |
|  | *Dinogymnium marthae* (Deflandre) Evitt, Clarke & Verdier | [306] |
| *Gymnodinium muticum* Vozzhennikova 1967 | |  |
| *Gymnodinium nelsonense* Cookson | |  |
|  | *Dinogymnium nelsonense* (Cookson) Evitt, Clarke & Verdier | [306] |
| *Gymnodinium parvimarginatum* Cookson & Eisenack | |  |
|  | *Scriniodinium parvimarginatum* (Cookson & Eisenack) Eisenack | [304] |
| *Gymnodinium pontismariae* Deflandre | |  |
| *Gymnodinium pontis-mariae* Deflandre | |  |
| *Gymnodinium sibiricum* Vozzhennikova | |  |
| *Gymnodinium sphaerocephalum* var. *laevis* Vozzhennikova | |  |
| *Gymnodinium sphaerocephalum* Vozzhennikova | |  |
| *Gymnodinium strombomorphum* Deflandre | |  |
|  | *Dinogymnium strombomorphum* (Deflandre) Evitt, Clarke & Verdier | [306] |
| *Gymnodinium torulosum* Deflandre | |  |
| *Gymnodinium ventriosum* Alberti | |  |
| *Gymnodinium westralium* Cookson & Eisenack | |  |
|  | *Dinogymnium westralium* (Cookson & Eisenack) Evitt, Clarke & Verdier | [308] |
