# Supplementary material for: The Taxonomic Significance of Species That Have Only Been Observed Once: The Genus Gymnodinium (Dinoflagellata) as an Example
Source: PLoS One. 2012 Aug 30;7(8):e44015. doi: 10.1371/journal.pone.0044015 (PMC3431360; doi:10.1371/journal.pone.0044015)
Supplement: Appendix S4 — Names of Gymnodinium no longer associated with the genus [309]–[337]. The current name and/or the reason for rejecting the name is given. A name is listed as not code compliant if it is used without the existence of an original description. A name is listed as erroneous if it is an incorrect combination of genus name and species epithet. (DOCX) [file pone.0044015.s004.docx]

Appendix S4

| **Gymnodinium name** | **Correct Name or Reason for Rejecting** | **Reference** |
| --- | --- | --- |
| *Gymnodinium accuminatum* | *Biecheleria pseudopalustris* (Schiller) Moestrup, Lindberg & Daugbjerg 2009 | [12,309] |
| *Gymnodinium acrotrochum* | *Gyrodinium acrotrochum* Larsen 1996 | [72] |
| *Gymnodinium acuminatum* Christen | *Biecheleria pseudopalustris* (Schiller) Moestrup, Lindberg & Daugbjerg 2009 | [12,309] |
| *Gymnodinium acutissimum* Okolodkov 1997 | Not code compliant |  |
| *Gymnodinium agile* Herdman 1922 | *Herdmania litoralis* Dodge 1981 | [310] |
| *Gymnodinium agilis* | *Herdmania litoralis* Dodge 1981 | [310] |
| *Gymnodinium alatum* Litvienko 1963 | *Peridiniopsis berolinense* (Lemmermann) Bourrelly 1968 | [12] |
| *Gymnodinium alba* | Not code compliant |  |
| *Gymnodinium albida* Lackey & Lackey 1970 | Not code compliant |  |
| *Gymnodinium albidum* Lackey & Lackey 1970 | Not code compliant |  |
| *Gymnodinium amphibinioides* Geitler 1924 | *Amphidinium amphidinioides* (Geitler) Schiller 1932 | [311] |
| *Gymnodinium amphidinioides* Geitler 1924 | *Amphidinium amphidinioides* (Geitler) Schiller 1932 | [311] |
| *Gymnodinium archimedes* Pouchet 1883 | *Cochlodinium archimedes* (Pouchet) Lemmermann 1899 | [312] |
| *Gymnodinium archimedis* Pouchet 1883 | *Cochlodinium archimedes* (Pouchet) Lemmermann 1899 | [312] |
| *Gymnodinium astigmatica* Christen 1959 | Not code compliant |  |
| *Gymnodinium asymmetricum* Massart 1900 | *Katodinium asymmetricum* (Massart) Loeblich | [313] |
| *Gymnodinium bei* | *Pelagodinium béii* (Spero) Siano et al. 2010 | [314] |
| *Gymnodinium béii* H.J. Spero 1987 | *Pelagodinium béii* (Spero) Siano et al. 2010 | [314] |
| *Gymnodinium bicaudatum* Pavillard 1905 | *Heterodinium bicaudatum* (Pavillard) Kofoid & Swezy 1921 | [19] |
| *Gymnodinium bisetosum* Lindemann | *Cystodinium bisetosum* (Lindemann) Huber-Pestalozzi 1950 | [185] |
| *Gymnodinium blax* Harris 1939 | *Katodinium fungiforme* (Anissimova) Loeblich 1965 | [12] |
| *Gymnodinium bohemicum* Fott 1938 | *Katodinium bohemicum* (Fott) Litvinenko 1977 | [12] |
| *Gymnodinium boreale* Gaarder | Not code compliant | [315] |
| *Gymnodinium brene* | *Karenia brevis* (Davis) Hansen and Moestrup 2000 | [7] |
| *Gymnodinium breve* Davis 1948 | *Karenia brevis* (Davis) Hansen and Moestrup 2000 | [7] |
| *Gymnodinium brevis* | *Karenia brevis* (Davis) Hansen and Moestrup 2000 | [7] |
| *Gymnodinium brevisulcatum* F.H. Chang | *Karenia brevisulcata* (Chang) Hansen and Moestrup 2000 | [7] |
| *Gymnodinium carinatum* var. *hiemalis* Woloszynska 1917 | *Woloszynskia pascheri* (Suchlandt) von Stosch 1973 | [12] |
| *Gymnodinium carinatum* Schilling 1891 | *Biecheleria pseudopalustris* (Schiller) Moestrup, Lindberg & Daugbjerg 2009 | [12,309] |
| *Gymnodinium cestocoetes* Thompson 1947 | *Woloszynskia cestocoedes* (Thompson) Thompson 1950 | [12] |
| *Gymnodinium chiasmonetrium* Norris 1961 | Erroneously formed name, *Gyrodinium chiasmonetrium* Norris 1961 |  |
| *Gymnodinium chloroforum* | *Lepidodinium chlorophorum* (Elbrächter and Schnepf) Hansen et al. 2007 | [316] |
| *Gymnodinium chlorophorum* Elbrächter & Schnepf 1996 | *Lepidodinium chlorophorum* (Elbrächter and Schnepf) Hansen et al. 2007 | [316] |
| *Gymnodinium cladochroma* | *Takayama cladochroma* (Larsen) de Salas et al. 2003 | [317] |
| *Gymnodinium cladochromum* | *Takayama cladochroma* (Larsen) de Salas et al. 2003 | [317] |
| *Gymnodinium coerulea* | *Balechina coerulea* (Dogiel) Taylor 1976 | [318] |
| *Gymnodinium coeruleum* Dogiel 1906 | *Balechina coerulea* (Dogiel) Taylor 1976 | [318] |
| *Gymnodinium conicum* Kofoid & Swezy 1921 | *Spatulodinium pseudonoctiluca* (Pouchet) Cachon and Cachon 1969 | [319] |
| *Gymnodinium constrictum* Schütt 1895 | *Cochlodinium constrictum* (Schütt) Lemmermann 1899 | [312] |
| *Gymnodinium contortum* Schütt 1895 | *Gyrodinium contortum* (Schütt) Kofoid and Swezy 1921 | [19] |
| *Gymnodinium cornutum* Pouchet | *Gyrodinium cornutum* (Pouchet) Kofoid and Swezy 1921 | [19] |
| *Gymnodinium cornutum* Schütt 1895 | *Gyrodinium schuetti* (Schütt) Kofoid and Swezy 1921 | [19] |
| *Gymnodinium coronatum* var. *glabrum* Woloszynska | *Tovellia glabra* (Woloszynska) Moestrup et al. 2005 | [320] |
| *Gymnodinium coronatum* Woloszynska 1917 | *Tovellia coronata* (Woloszynska) Moestrup et al. 2005 | [320] |
| *Gymnodinium corsicum* | Erroneously formed name, *Gyrodinium corsicum* Paulmier |  |
| *Gymnodinium costatum* var. *glabrum* Woloszynska | *Gyrodinium coronatum* var. *glabrum* Woloszynska |  |
| *Gymnodinium crassum* Pouchet 1885 | *Gyrodinium crassum* (Pouchet) Kofoid and Swezy 1921 | [19] |
| *Gymnodinium cruciatum* (Massart) Schiller | Rejected by Silva as nomen nudum | ucjeps.berkeley.edu |
| *Gymnodinium cruciatum* J. Massart | *Chilodinium cruciatum* Massart 1920 | [321] |
| *Gymnodinium cuneatum* | Erroneously fomed name, *Gyrodinium cuneatum* Kofoid & Swezy 1921 |  |
| *Gymnodinium cuspidatum* Cleve-Euler | Rejected by Silva as nomen nudum | ucjeps.berkeley.edu |
| *Gymnodinium decussata* | Not code compliant |  |
| *Gymnodinium dextrorsum* | Not code compliant |  |
| *Gymnodinium dimorphe* Baumeister 1938 | *Cystodinium phaseolus* Pascher 1928 | [12] |
| *Gymnodinium diplococcus* Cleve-Euler | Rejected by Silva as nomen nudum | ucjeps.berkeley.edu |
| *Gymnodinium dorsum* | Erroneosuly formed name, *Gyrodinium dorsum* Kofoid & Swezy 1921 |  |
| *Gymnodinium dubium* Cleve-Euler | Rejected by Silva as nomen nudum | ucjeps.berkeley.edu |
| *Gymnodinium edax* Schilling | Erroneously formed name, *Glenodinium edax* Schilling |  |
| *Gymnodinium elonga* | *Lessardia elongata* Saldarriaga & Taylor | [322] |
| *Gymnodinium elongatum* Hope 1954 | *Lessardia elongata* Saldarriaga & Taylor | [322] |
| *Gymnodinium estuariale* | Erroneously formed name, *Gyrodinium estuariale* Hulbert 1957 |  |
| *Gymnodinium exavatum* | *Biecheleria pseudopalustris* (Schiller) Moestrup, Lindberg & Daugbjerg 2009 | [12,309] |
| *Gymnodinium excavatum* var. *dextrorsum* | *Biecheleria pseudopalustris* (Schiller) Moestrup, Lindberg & Daugbjerg 2009 | [12,309] |
| *Gymnodinium excavatum* Nygaard | *Biecheleria pseudopalustris* (Schiller) Moestrup, Lindberg & Daugbjerg 2009 | [12,309] |
| *Gymnodinium excavatum* var. *dextrosum* Nygaard 1954 | *Biecheleria pseudopalustris* (Schiller) Moestrup, Lindberg & Daugbjerg 2009 | [12,309] |
| *Gymnodinium faba* Cleve-Euler | Rejected by Silva as nomen nudum | ucjeps.berkeley.edu |
| *Gymnodinium falcatum* | Erroneously formed name, *Gyrodinium falcatum* Kofoid and Swezy 1921 |  |
| *Gymnodinium fissoides* | Erroneously formed name, *Gyrodinium fissoides* Elbrachter 1979 |  |
| *Gymnodinium fissum* Levander 1894 | *Gyrodinium fissum* (Leavander) Kofoid and Swezy 1921 | [19] |
| *Gymnodinium foliaceum* Stein 1919 | Erroneously formed name, *Glenodinium foliaceum* Stein 1919 |  |
| *Gymnodinium formica* | *Karlodinium veneficum* (Ballantine) Larsen 2000 | [7] |
| *Gymnodinium fucorum* Kuster 1908 | *Crypthecodinium cohnii* (Seligo) Chatton 1952 | [323] |
| *Gymnodinium fungiforme* Schiller 1933 | *Katodinium fungiforme* (Anissimova) Loeblich 1965 | [313] |
| *Gymnodinium fungiforme* Anisimova 1925 | *Katodinium fungiforme* (Anissimova) Loeblich 1965 | [313] |
| *Gymnodinium fusiforme* Kofoid & Swezy | Erroneously formed name, *Gyrodinium fusiforme* Kofoid & Swezy 1921 |  |
| *Gymnodinium fusum* | *Gyrodinium falcatum* Kofoid and Swezy 1921 | [19] |
| *Gymnodinium fusus* Schütt 1895 | *Gyrodinium falcatum* Kofoid and Swezy 1921 | [19] |
| *Gymnodinium galatheanum* Braarud 1957 | *Karlodinium veneficum* (Ballantine) Larsen 2000 | [7] |
| *Gymnodinium geminatum* Schütt 1895 | *Cochlodinium geminatum* (Schütt) Lemmermann 1899 | [312] |
| *Gymnodinium glabra* Woloszynska 1918 | *Tovellia glabra* (Woloszynska) Moestrup et al. 2005 | [320] |
| *Gymnodinium glaciale* Danysz 1886 | Not code compliant | [324] |
| *Gymnodinium glandula* Herdman 1924 | *Katodinium glandulum* (Herdman) Loeblich 1965 | [313] |
| *Gymnodinium glaucum* Conrad 1926 | *Amphidinium conradii* (Conrad) Schiller 1933 | [21] |
| *Gymnodinium gracile* var. *exiguum* Pouchet 1883 | Rejected by Kofoid and Swezy as nomen nudum | [19] |
| *Gymnodinium gracilis* Lackey | Rejected by Silva as nomen nudum | ucjeps.berkeley.edu |
| *Gymnodinium grave* | Erroneously formed name, *Gyrodinium grave* (Meunier) Kofoid and Swezy 1921 |  |
| *Gymnodinium halophila* | *Biecheleria baltica* (Biecheler) Moestrup, Lindberg & Daugbjerg 2009 | [309] |
| *Gymnodinium halophilum* Biecheler 1952 | *Biecheleria baltica* (Biecheler) Moestrup, Lindberg & Daugbjerg 2009 | [309] |
| *Gymnodinium helicoides* | Not code compliant |  |
| *Gymnodinium helix* Schütt 1895 | *Cochlodinium helix* (Pouchet) Lemmermann 1899 | [312] |
| *Gymnodinium helix* Pouchet 1887 | *Cochlodinium helix* (Pouchet) Lemmermann 1899 | [312] |
| *Gymnodinium helveticum* f. *achroum* Skuja 1948 | *Gyrodinium helveticum* (Penard) Takano & Horiguchi 2004 | [325] |
| *Gymnodinium helveticum* var. *apiculatum* | *Gyrodinium helveticum* (Penard) Takano & Horiguchi 2004 | [325] |
| *Gymnodinium helveticum* Penard 1891 | *Gyrodinium helveticum* (Penard) Takano & Horiguchi 2004 | [325] |
| *Gymnodinium helveticum* var. *apiculata* (Zacharias) Utermöhl | *Gyrodinium helveticum* (Penard) Takano & Horiguchi 2004 | [325] |
| *Gymnodinium hiemale* Skvortzov 1927 | *Woloszynskia pascheri* (Suchlandt) von Stosch 1973 | [12] |
| *Gymnodinium hiemale* Woloszynska 1917 | *Woloszynskia pascheri* (Suchlandt) von Stosch 1973 | [12] |
| *Gymnodinium hyalinum* Schilling 1891 | *Gyrodinium hyalinum* (Schilling) Kofoid and Swezy 1921 | [19] |
| *Gymnodinium inversum* Nygaard 1949 | *Woloszynskia pascheri* (Suchlandt) von Stosch 1973 | [12] |
| *Gymnodinium inversum* var. *elongatum* Nygaard 1949 | *Woloszynskia pascheri* (Suchlandt) von Stosch 1973 | [12] |
| *Gymnodinium lachryma* (Meunier) Kofoid & Swezy 1921 | Erroneously formed name, *Gyrodinium lachryma* (Meunier) Kofoid & Swezy 1921 |  |
| *Gymnodinium latum* Cleve-Euler | Rejected by Silva as nomen nudum | ucjeps.berkeley.edu |
| *Gymnodinium lebourae* Pavillard | *Spatulodinium pseudonoctiluca* (Pouchet) Cachon and Cachon 1969 | [319] |
| *Gymnodinium lebouriae* Pavillard 1924 | *Spatulodinium pseudonoctiluca* (Pouchet) Cachon and Cachon 1969 | [319] |
| *Gymnodinium lebourii* Pavillard | *Spatulodinium pseudonoctiluca* (Pouchet) Cachon and Cachon 1969 | [319] |
| *Gymnodinium lens* Fott 1957 | *Borghiella tenuissima* (Lauterborn) Moestrup et al. 2008 | [326] |
| *Gymnodinium lenticula* | Erroneously formed name, *Glenodinium lenticula* Pouchet |  |
| *Gymnodinium leopoliense* Woloszynska 1917 | *Tovellia leopoliensis* (Woloszynska) Moestrup et al. 2005 | [320] |
| *Gymnodinium leopoliensis* Woloszynska 1917 | *Borghiella tenuissima* (Lauterborn) Moestrup et al. 2008 | [326] |
| *Gymnodinium limneticum* Lackey 1936 | Not code compliant |  |
| *Gymnodinium linguliferum* Lebour | Erroneously formed name, *Gyrodinium lingulifera* Lebour 1925 |  |
| *Gymnodinium linucheae* Trench & Thinh 1995 | *Symbiodinium linucheae* (Trench & Thinh) Lajeunesse | [327] |
| *Gymnodinium loburare* Campbell 1973 | Not code compliant |  |
| *Gymnodinium marianae* | *Balechina marianae* Taylor | [318] |
| *Gymnodinium medium* Cleve-Euler | Rejected by Silva as nomen nudum | ucjeps.berkeley.edu |
| *Gymnodinium metum* Hulburt 1957 | Not code compliant |  |
| *Gymnodinium microadriaticum* (Freudenthal) Taylor 1971 | *Symbiodinium microadriaticum* Freudenthal 1962 | [328] |
| *Gymnodinium micrum* (Leadbeater and Dodge) Loeblich 1970 | *Karlodinium veneficum* (Ballantine) Larsen 2000 | [7] |
| *Gymnodinium mikimotoi*Miyake & Kominami 1935 | *Karenia mikimotoi* (Miyake and Kominami) Hansen and Moestrup 2000 | [7] |
| *Gymnodinium mikimotoii* | *Karenia mikimotoi* (Miyake and Kominami) Hansen and Moestrup 2000 | [7] |
| *Gymnodinium mikinotoi* | *Karenia mikimotoi* (Miyake and Kominami) Hansen and Moestrup 2000 | [7] |
| *Gymnodinium minutissimum* Massart 1901 | Rejected by Silva as nomen nudum | ucjeps.berkeley.edu |
| *Gymnodinium minutum* Lebour 1925 | *Heterocapsa rotundata* (Lohmann) Hansen 1995 | [329] |
| *Gymnodinium mirum* Utermöhl 1923 | *Woloszynskia pascheri* (Suchlandt) von Stosch 1973 | [12] |
| *Gymnodinium monadicum* (Perty) Saville-Kent 1880 | *Katodinium monadicum* (Perty) Javornicky | [12] |
| *Gymnodinium musaei* Danysz 1887 | *Katodinium woloszynskae* (Schiller) Loeblich 1965 | [313] |
| *Gymnodinium musei* Danysz 1887 | *Katodinium woloszynskae* (Schiller) Loeblich 1965 | [12] |
| *Gymnodinium nagasakiense*Takayama and Adachi 1985 | *Karenia mikimotoi* (Miyake and Kominami) Hansen and Moestrup 2000 | [7] |
| *Gymnodinium nagasakiensis* | *Karenia mikimotoi* (Miyake and Kominami) Hansen and Moestrup 2000 | [7] |
| *Gymnodinium nagasakii* | *Karenia mikimotoi* (Miyake and Kominami) Hansen and Moestrup 2000 | [7] |
| *Gymnodinium nasutum* (Stein) Levander 1900 | *Hemidinium nasutum* Kofoid & Swezy 1921 | [19] |
| *Gymnodinium natalense* Horiguchi & Pienaar 1994 | *Biecheleria natalensis* (Horiguchi and Pienaar) Moestrup 2009 | [309] |
| *Gymnodinium neglectum* (Schilling) Lindemann 1929 | *Woloszynskia neglecta* (Schilling) Thompson 1950 | [12] |
| *Gymnodinium neglectum* var. *astigmata* Christen 1959 | *Woloszynskia neglecta* (Schilling) Thompson 1950 | [12] |
| *Gymnodinium neglectum* var. *astigmatica* Christen 1959 | *Woloszynskia neglecta* (Schilling) Thompson 1950 | [12] |
| *Gymnodinium nelsoni* Martin 1929 | *Akashiwo sanguinea* (Hirasaka) Hansen and Moestrup 2000 | [7] |
| *Gymnodinium nelsonii* Martin 1929 | *Akashiwo sanguinea* (Hirasaka) Hansen and Moestrup 2000 | [7] |
| *Gymnodinium noctiluca* Pouchet 1885 | *Noctiluca miliaris* Kofoid & Swezy | [19] |
| *Gymnodinium novaculosum* Baumeister 1939 | *Cystodinium novaculosum* (Baumeister) Huber-Pestalozzi | [12] |
| *Gymnodinium nygaardi* Christen 1958 | *Tovellia nygaardii* (Christen) Moestrup et al. 2005 | [320] |
| *Gymnodinium nygaardii* Christen 1958 | *Tovellia nygaardii* (Christen) Moestrup et al. 2005 | [320] |
| *Gymnodinium oblongum* | Erroneously formed name, *Gyrodinium oblongum* Larsen & Patterson 1990 |  |
| *Gymnodinium oligoplacatum* Skuja 1956 | *Perdinium umbonatum* Stein 1883 | [12] |
| *Gymnodinium opimum* Schütt 1895 | *Gyrodinium opimum* (Schütt) Lebour 1925 | [20] |
| *Gymnodinium ordinatum* Skuja 1939 | *Woloszynskia ordinata* (Skuja) Thompson 1950 | [12] |
| *Gymnodinium ordinatum* var. *sparsum* Popovsky 1971 | *Woloszynskia ordinata* (Skuja) Thompson 1950 | [12] |
| *Gymnodinium ovatum* Gourret 1883 | *Gyrodinium ovatum* (Gourret) Kofoid and Swezy 1921 | [19] |
| *Gymnodinium ovum* Schütt 1895 | *Gyrodinium ovum* (Schütt) Kofoid and Swezy 1921 | [19] |
| *Gymnodinium palustre* A. J. Schilling 1891 | *Biecheleria pseudopalustris* (Schiller) Moestrup, Lindberg & Daugbjerg 2009 | [12,309] |
| *Gymnodinium parasitica* | *Oodinium parasiticum* (Dogiel) Kofoid and Swezy 1921 | [19] |
| *Gymnodinium parasiticum* Dogiel 1906 | *Oodinium parasiticum* (Dogiel) Kofoid and Swezy 1921 | [19] |
| *Gymnodinium parvulum* Schutt 1895 | *Gyrodinium parvulum* (Schütt) Kofoid and Swezy 1921 | [19] |
| *Gymnodinium pascheri* (Suchlandt) Schiller 1954 | *Woloszynskia pascheri* (Suchlandt) von Stosch 1973 | [12] |
| *Gymnodinium pellucidum* Wulff 1919 | *Gyrodinium pellucidum* (Wulff) Schiller 1933 | [21] |
| *Gymnodinium pepo* | Erroneously formed name, *Gyrodinium pepo* (Schütt) Kofoid and Swezy 1921 |  |
| *Gymnodinium peridinium* | Not code compliant | [330] |
| *Gymnodinium pigmentosum* (Dodge) Loeblich 1970 | *Aureodinium pigmentosum* Dodge 1967 | [331] |
| *Gymnodinium pirum* Schütt 1895 | *Cochlodinium pirum* (Schütt) Lemmermann 1921 | [312] |
| *Gymnodinium polonicum* (Woloszynska) Woloszynska 1917 | *Woloszynskia pascheri* (Suchlandt) von Stosch 1973 | [12] |
| *Gymnodinium polyphemus* var. *nigrum* Pouchet 1887 | *Protospis nigra* (Pouchet) Kofoid and Swezy 1921 | [19] |
| *Gymnodinium polyphemus* var. *roseum* Pouchet Pouchet 1897 | *Pouchetia roseum* (Pouchet) Kofoid and Swezy 1921 | [19] |
| *Gymnodinium polyphemus* Pouchet 1885 | *Warnowia polyphemus* (Pouchet) Schiller 1933 | [21] |
| *Gymnodinium polyphemus* var. *magna* Dogiel 1906 | Rejected by Kofoid and Swezy as nomen nudum | [19] |
| *Gymnodinium poucheti* Lemmermann 1899 | *Oodinium pouchetii* (Lemmermann) Chatton 1912 | [19] |
| *Gymnodinium pouchetii* Lemmermann 1899 | *Oodinium pouchetii* (Lemmermann) Chatton 1912 | [19] |
| *Gymnodinium pouchettii* | *Oodinium pouchetii* (Lemmermann) Chatton 1912 | [19] |
| *Gymnodinium prunus* | Erroneously formed name, *Gyrodinium prunus* (Wulff) Lebour 1925 |  |
| *Gymnodinium pseudonoctiluca* Pouchet 1885 | *Spatulodinium pseudonoctiluca* (Pouchet) Cachon and Cachon 1969 | [319] |
| *Gymnodinium pseudopalustre* | *Biecheleria pseudopalustris* (Schiller) Moestrup et al. 2009 | [309] |
| *Gymnodinium pseudopalustre* Woloszynska | *Biecheleria pseudopalustris* (Schiller) Moestrup, Lindberg & Daugbjerg 2009 | [12,309] |
| *Gymnodinium pseudopalustris* Schiller 1932 | *Biecheleria pseudopalustris* (Schiller) Moestrup, Lindberg & Daugbjerg 2009 | [309] |
| *Gymnodinium pulchellum*Larsen 1994 | *Takayama pulchella* (Larsen) de Salas et al. 2003 | [317] |
| *Gymnodinium pulvisculus* (Ehrenberg) Stein 1878 | Erroneously formed name, *Glenodinium pulvisculus* (Ehrenberg) Stein | [31] |
| *Gymnodinium pulvisculus* Pouchet 1885 | *Oodinium pouchetii* (Lemmermann) Chatton 1912 | [332] |
| *Gymnodinium pulvisculus* var. *oculatum* Largajolli 1907 | *Glenodinium pulvisculus* (Ehrenberg) Stein | [333] |
| *Gymnodinium pusillum* Schilling 1891 | *Gyrodinium pusillum* (Schilling) Kofoid and Swezy 1921 | [19] |
| *Gymnodinium pyrenoidosum* Horiguchi & Chihara 1988 | Needs to be placed in new genus | [334] |
| *Gymnodinium quadridens* | *Peridiniopsis quadridens* (Thompson) Bourrelly | [12] |
| *Gymnodinium quadrilobatum* Horguchi & Pienaar 1994 | Needs to be placed in new genus | [335] |
| *Gymnodinium rarum* Litvinenko 1963 | *Amphidinium elenkinii* Skvortsov 1925 | [311] |
| *Gymnodinium resplendens* Hulburt 1975 | Erroneously formed name, *Gyrodinium resplendens* Hulburt 1957 |  |
| *Gymnodinium robusta* | Misspelling of nomen nudum *Gymnodinium robustum* Cleve-Euler |  |
| *Gymnodinium robustum* Cleve-Euler | Rejected by Silva as nomen nudum | ucjeps.berkeley.edu |
| *Gymnodinium roseum* Dogiel 1906 | *Chytriodinium roseum* (Dogiel) Chatton 1912 | [19] |
| *Gymnodinium rubrum* Kofoid & Swezy 1921 | *Gyrodinium rubrum* (Kofoid & Swezy) Takano & Horiguchi 2004 | [325] |
| *Gymnodinium sangineum* | *Akashiwo sanguinea* (Hirasaka) Hansen and Moestrup 2000 | [7] |
| *Gymnodinium sanguineum* Hirasaka 1922 | *Akashiwo sanguinea* (Hirasaka) Hansen and Moestrup 2000 | [7] |
| *Gymnodinium simplex* (Lohmann) Kofoid & Swezy 1921 | Needs to be placed in new genus | [309] |
| *Gymnodinium simplicissimum* Stein 1878 | Not code compliant |  |
| *Gymnodinium skvortzovii* Schiller 1933 | *Woloszynskia pascheri* (Suchlandt) von Stosch 1973 | [12] |
| *Gymnodinium skvortzowii* | *Woloszynskia pascheri* (Suchlandt) von Stosch 1973 | [12] |
| *Gymnodinium spinifera* Claparède & Lachmann | Erroneously formed name, *Gonyaulax spinifera* (Claparède & Lachmann) Diesing |  |
| *Gymnodinium spirale* var. *acuta* Schütt 1895 | *Gyrodinium acutum* (Schütt) Kofoid and Swezy 1921 | [19] |
| *Gymnodinium spirale* var. *cornutum* Pouchet 1885 | *Gyrodinium cornutum* (Pouchet) Kofoid and Swezy 1921 | [19] |
| *Gymnodinium spirale* var. *mitra* Schütt, 1895 | *Gyrodinium mitrum* Kofoid and Swezy 1921 | [19] |
| *Gymnodinium spirale* var. *obtusa* Schütt 1895 | *Gyrodinium obtusum* (Schütt) Kofoid and Swezy 1921 | [7] |
| Gymnodinium *spirale* var. *pepo* Schütt 1895 | *Gyrodinium pepo* (Schütt) Kofoid and Swezy 1921 | [7] |
| Gymnodinium *spirale* var. *pinguis* Schütt 1895 | *Gyrodinium pingue* (Schütt) Kofoid and Swezy 1921 | [7] |
| *Gymnodinium spirale* var. *striatum* Pouchet 1883 | *Gyrodinium striatum* Kofoid & Swezy 1921 | [19] |
| *Gymnodinium spirale* Bergh 1881 | *Gyrodinium spirale* (Bergh) Kofoid and Swezy 1921 | [19] |
| *Gymnodinium splendens* Lebour 1925 | *Akashiwo sanguinea* (Hirasaka) Hansen and Moestrup 2000 | [7] |
| *Gymnodinium stagnale* J. Schiller 1955 | *Prosoaulax lacustris* (Stein) Calado and Moestrup 2005 | [311] |
| *Gymnodinium stagnate* | *Prosoaulax lacustris* (Stein) Calado and Moestrup 2005 | [311] |
| *Gymnodinium stigmaticum* Lindemann | *Katodinium stigmaticum* (Lindemann) Loeblich 1965 | [313] |
| *Gymnodinium strangulatum* Schütt 1895 | *Cochlodinium strangulatum* (Schütt) Schütt 1896 | [312] |
| *Gymnodinium striassimum* | Erroneously formed name, *Gyrodinium striatissimum* (Hulburt) Hansen and Moestrup 2000 | [7] |
| *Gymnodinium striata* | *Gyrodinium striatissimum* (Hulburt) Hansen and Moestrup 2000 | [7] |
| *Gymnodinium striatissimum* Hulburt 1957 | *Gyrodinium striatissimum* (Hulburt) Hansen and Moestrup 2000 | [7] |
| *Gymnodinium striatum* | *Gyrodinium striatissimum* (Hulburt) Hansen and Moestrup 2000 | [7] |
| *Gymnodinium sugashimanii* Cachon et al. 1989 | *Gyrodinium falcatum* Kofoid and Swezy 1921 | [336] |
| *Gymnodinium tamarensis* | Erroneously formed name, *Gonyaulax tamarensis* Lebour |  |
| *Gymnodinium tatricum* Woloszynska 1919 | *Woloszynskia pascheri* (Suchlandt) von Stosch 1973 | [12] |
| *Gymnodinium tenuissimum* Lauterborn 1894 | *Borghiella tenuissima* (Lauterborn) Moestrup et al. 2008 | [326] |
| *Gymnodinium teredo* Pouchet | *Torodinium teredo* (Pouchet) Kofoid and Swezy 1921 | [19] |
| *Gymnodinium trigonocephalum* Cleve-Euler | Rejected by Silva as nomen nudum | ucjeps.berkeley.edu |
| *Gymnodinium tripos* | Erroneously formed name, *Ceratium tripos* (Müller) Ehrenberg |  |
| *Gymnodinium tripos* var. *ponctic* | Erroneously formed name, *Ceratium tripos* var. *ponticum* Jorgensen |  |
| *Gymnodinium trochoideum* Stein | Erroneously formed name, *Glenodinium trochoideum* Stein |  |
| *Gymnodinium tylota* | *Woloszynskia tylota* (Mapletoft et al.) Bibby and Dodge 1972 | [12] |
| *Gymnodinium tylotum* Mapletoft et al. 1966 | *Woloszynskia tylota* (Mapletoft et al.) Bibby and Dodge 1972 | [320] |
| *Gymnodinium undulans* | Erroneously formed name, *Gyrodinium undulans* Hulburt |  |
| *Gymnodinium undulatum* Woloszynska 1925 | *Woloszynskia pascheri* (Suchlandt) von Stosch 1973 | [12] |
| *Gymnodinium vation* | Not code compliant |  |
| *Gymnodinium veneficum* Stein 1878 | *Karlodinium veneficum* (Ballantine) Larsen 2000 | [7] |
| *Gymnodinium veneficum* Ballantine 1956 | *Karlodinium veneficum* (Ballantine) Larsen 2000 | [7] |
| *Gymnodinium veris* E. Lindemann 1925 | *Woloszynskia pascheri* (Suchlandt) von Stosch 1973 | [12] |
| *Gymnodinium vertebralis* | Rejected by Silva as nomen nudum | ucjeps.berkeley.edu |
| *Gymnodinium vinugo* | *Karlodinium vitiligo* (Ballantine) Larsen 2000 | [7] |
| *Gymnodinium virescens* Wood 1963 | Not code compliant |  |
| *Gymnodinium viride* Schütt 1895 | *Gyrodinium viride* Kofoid & Swezy 1921 | [19] |
| *Gymnodinium viridis* Lebour 1917 | *Spatulodinium pseudonoctiluca* (Pouchet) Cachon and Cachon 1969 | [319] |
| *Gymnodinium vitiligo* Ballantine 1956 | *Karlodinium vitiligo* (Ballantine) Larsen 2000 | [7] |
| *Gymnodinium vorax* Massart | Rejected by Silva as nomen nudum | [337] |
| *Gymnodinium vorticella* Stein | *Katodinium vorticellum* (Stein) Loeblich 1965 | [313] |
| *Gymnodinium wigrense* Woloszynska 1925 | *Biecheleria pseudopalustris* (Schiller) Moestrup, Lindberg & Daugbjerg 2009 | [12,309] |
| *Gymnodinium woloszynskae* Pascher | *Woloszynskia pascheri* (Suchlandt) von Stosch 1973 | [12] |
| *Gymnodium pigmentosum* | *Aureodinium pigmentosum* Dodge 1967 | [331] |
