# Supplementary material for: The Taxonomic Significance of Species That Have Only Been Observed Once: The Genus Gymnodinium (Dinoflagellata) as an Example
Source: PLoS One. 2012 Aug 30;7(8):e44015. doi: 10.1371/journal.pone.0044015 (PMC3431360; doi:10.1371/journal.pone.0044015)
Supplement: Appendix S5 — List of species of Gymnodinium following removal of oncers that do not meet the selection criteria used here. (DOCX) [file pone.0044015.s005.docx]

Appendix S5

*Gymnodinium absumens* Schiller 1957

*Gymnodinium achromaticum* Lebour 1917

*Gymnodinium adriaticum* (Schmarda) Kofoid & Swezy 1921

*Gymnodinium aequatoriale* Hasle 1960

*Gymnodinium aeruginosum* Stein 1883

*Gymnodinium aesculum* Baumeister 1943

*Gymnodinium affine* Dogiel 1906

*Gymnodinium agaricoides* Campbell 1973

*Gymnodinium agiliforme* Schiller 1928

*Gymnodinium alaskensis* Bursa 1963

*Gymnodinium allophron* Larsen 1994

*Gymnodinium amphiconicoides* Schiller 1957

*Gymnodinium amphityphlum* Larsen 1994

*Gymnodinium amphora* Kofoid & Swezy 1921

*Gymnodinium amplinucleum* Campbell 1973

*Gymnodinium antarcticum* Thessen, Patterson & Murray 2012

*Gymnodinium arcticum* Wulff 1919

*Gymnodinium arcuatum* Kofoid 1931

*Gymnodinium arenicolus* Dragesco 1965

*Gymnodinium armoricanum* Villeret 1953

*Gymnodinium atomatum* Larsen 1994

*Gymnodinium attenuatum* Kofoid & Swezy 1921

*Gymnodinium aurantium* Campbell 1973

*Gymnodinium auratum* Kofoid & Swezy 1921

*Gymnodinium aureolum* (Hulburt) Hansen 2000

*Gymnodinium aureum* Kofoid & Swezy 1921

*Gymnodinium australe* Playfair 1919

*Gymnodinium australense* Ruinen 1938

*Gymnodinium austriacum* Schiller 1933

*Gymnodinium manchuriensis* Thessen, Patterson & Murray 2012

*Gymnodinium baccatum* Balech 1965

*Gymnodinium baicalense* Antipova 1955

*Gymnodinium baumeisteri* Schiller 1957

*Gymnodinium biciliatum* Ohno 1911

*Gymnodinium biconicum* Schiller 1928

*Gymnodinium bicorne* Kofoid & Swezy 1921

*Gymnodinium bifurcatum* Kofoid & Swezy 1921

*Gymnodinium birotundatum* van Goor 1925

*Gymnodinium bisaetosum* Lindemann 1928

*Gymnodinium boguensis* Campbell 1973

*Gymnodinium bonaerense* Akselman 1983

*Gymnodinium caerulescens* Schiller 1957

*Gymnodinium campbelli* Thessen, Patterson & Murray 2012

*Gymnodinium canus* Kofoid & Swezy 1921

*Gymnodinium capitatum* Conrad & Kufferath 1954

*Gymnodinium caput* Schiller 1928

*Gymnodinium cassiei* Norris 1961

*Gymnodinium catenatum* Graham 1943

*Gymnodinium chiastosporum* (Harris) Cridland 1958

*Gymnodinium chukwanii* Ballantine 1961

*Gymnodinium cinctum* Kofoid & Swezy 1921

*Gymnodinium cnecoides* Harris 1940

*Gymnodinium coeruleum* Dogiel 1906

*Gymnodinium colymbeticum* Harris 1940

*Gymnodinium concavum* Skvortzov 1968

*Gymnodinium conicum* Kofoid & Swezy 1921

*Gymnodinium contractum* Kofoid & Swezy 1921

*Gymnodinium corii* Schiller 1928

*Gymnodinium corollarium* Sundström, Kremp & Daugbjerg 2009

*Gymnodinium corpusculum* (Perty) Saville-Kent 1880/81

*Gymnodinium costatum* Kofoid & Swezy 1921

*Gymnodinium cryophilum* (Wedemayer, Wilcox & Graham) Hansen & Moestrup 2000

*Gymnodinium cucumis* Schütt 1895

*Gymnodinium cyaneofungiforme* Conrad & Kufferath 1954

*Gymnodinium danicans* Campbell 1973

*Gymnodinium danubiense* Schiller 1957

*Gymnodinium deformabile* Schiller 1957

*Gymnodinium dentatum* Larsen 1994

*Gymnodinium devorans* Schiller 1957

*Gymnodinium diploconus* Schütt 1895

*Gymnodinium discoidale* Harris 1940

*Gymnodinium dissimile* Kofoid & Swezy 1921

*Gymnodinium dodgei* Sarma & Shyam 1974

*Gymnodinium dogieli* Kofoid & Swezy 1921

*Gymnodinium doma* Kofoid & Swezy 1921

*Gymnodinium dorsalisulcum* (Hulburt, McLaughlin & Zahl) Murray, de Salas & Hallegraeff 2007

*Gymnodinium endofasciculum* Campbell 1973

*Gymnodinium enorme* Ballantine 1964

*Gymnodinium eucyaneum* Hu 1983

*Gymnodinium eufrigidum* Schiller 1957

*Gymnodinium excavatum* van Meel 1969

*Gymnodinium exechegloutum* Norris 1961

*Gymnodinium filum* Lebour 1917

*Gymnodinium flavum* Kofoid & Swezy 1921

*Gymnodinium fossarum* Conrad & Kufferath 1954

*Gymnodinium fulgens* Kofoid & Swezy 1921

*Gymnodinium fuscum* (Ehrenberg) Stein 1883

*Gymnodinium galeaeforme* Matzenauer 1933

*Gymnodinium galeatum* Larsen 1994

*Gymnodinium galesianum* Campbell 1973

*Gymnodinium gelbum* Kofoid 1931

*Gymnodinium gibbera* Schiller 1928

*Gymnodinium glandiforme* Conrad & Kufferath 1954

*Gymnodinium glaucum* Schiller 1957

*Gymnodinium gleba* Schütt 1895

*Gymnodinium gracile* Bergh 1881/82

*Gymnodinium gracilentum* Campbell 1973

*Gymnodinium grammaticum* (Pouchet) Kofoid & Swezy 1921

*Gymnodinium granii* Schiller 1957

*Gymnodinium guttiforme* Larsen 1994

*Gymnodinium guttula* (Hada) Balech 1976

*Gymnodinium hamulus* Kofoid & Swezy 1921

*Gymnodinium herbaceum* Kofoid 1921

*Gymnodinium heterostriatum* Kofoid & Swezy 1921

*Gymnodinium hiemale* (Schiller) Popovsky 1990

*Gymnodinium hiroshimaensis* Hada 1968

*Gymnodinium huber-pestalozzii* Schiller 1957

*Gymnodinium hulburtii* Campbell 1973

*Gymnodinium impatiens* Skuja 1964

*Gymnodinium impudicum* (Fraga & Bravo) Hansen & Moestrup 2000

*Gymnodinium incertum* Herdman 1924

*Gymnodinium incisum* Kofoid & Swezy 1921

*Gymnodinium incoloratum* Conrad & Kufferath 1954

*Gymnodinium inconstans* van Meel 1969

*Gymnodinium indicum* Shyam & Sarma 1974

*Gymnodinium inerme* (Schmarda) Saville-Kent 1880/81

*Gymnodinium instriatum* (Freudenthal & Lee) Coats 2002

*Gymnodinium intercalaris* Bursa 1961

*Gymnodinium irregulare* Hope 1954

*Gymnodinium japonicum* Hada 1974

*Gymnodinium katodiniforme* Elbrächter & Schnepf 1979

*Gymnodinium klebsi* Lindemann 1928

*Gymnodinium knollii* Schiller 1957

*Gymnodinium kowalevskii* Pitzik 1967

*Gymnodinium kujavense* Liebetanz 1925

*Gymnodinium lachmanni* Saville-Kent 1880/81

*Gymnodinium lackeyi* (Lackey) Kiselev 1954

*Gymnodinium lacustre* Schiller 1933

*Gymnodinium lalitae* Sarma & Shyam 1974

*Gymnodinium lanskoi* Rouchijanen 1968

*Gymnodinium lantzschii* Utermöhl 1925

*Gymnodinium latum* Skuja 1948

*Gymnodinium lazulum* Hulburt 1957

*Gymnodinium legiconveniens* Schiller 1957

*Gymnodinium leptum* Norris 1961

*Gymnodinium limitatum* Skuja 1956

*Gymnodinium lineatum* Kofoid & Swezy 1921

*Gymnodinium lineopunicum* Kofoid & Swezy 1921

*Gymnodinium lira* Kofoid & Swezy 1921

*Gymnodinium litoralis* Reñé 2011

*Gymnodinium lobularis* Campbell 1973

*Gymnodinium lucidum* Ballantine 1964

*Gymnodinium lunula* Schütt 1895

*Gymnodinium maguelonnense* Biecheler 1939

*Gymnodinium marinum* Saville-Kent 1880/81

*Gymnodinium marylandicum* Thompson 1947

*Gymnodinium meervalli* Redeke 1919

*Gymnodinium microreticulatum* Bolch & Hallegraeff 1999

*Gymnodinium minor* Lebour 1917

*Gymnodinium minutulum* Larsen 1994

*Gymnodinium mitratum* Schiller 1933

*Gymnodinium modestum* Balech 1976

*Gymnodinium multilineatum* Kofoid & Swezy 1921

*Gymnodinium multistriatum* Kofoid & Swezy 1921

*Gymnodinium myriopyrenoides* Yamaguchi, Nakayama, Kai & Inouye 2011

*Gymnodinium najadeum* Schiller 1928

*Gymnodinium nanum* Schiller 1928

*Gymnodinium neapolitanum* Schiller 1928

*Gymnodinium nolleri* Ellegaard & Moestrup 1998

*Gymnodinium oceanicum* Hasle 1960

*Gymnodinium ochraceum* Kofoid 1931

*Gymnodinium octo* Larsen 1994

*Gymnodinium olivaceum* Skvortzov 1968

*Gymnodinium oppressum* Conrad 1926

*Gymnodinium ostenfeldi* Schiller 1928

*Gymnodinium ovulum* Kofoid & Swezy 1921

*Gymnodinium pachydermatum* Kofoid & Swezy 1921

*Gymnodinium pallidum* Skuja 1939

*Gymnodinium palustriforme* Hansen & Flaim 2007

*Gymnodinium paradoxiforme* Schiller 1957

*Gymnodinium paradoxum* Schilling 1891

*Gymnodinium parvum* Larsen 1994

*Gymnodinium patagonicum* Balech 1971

*Gymnodinium paulseni* Schiller 1928

*Gymnodinium pavlae* Popovsky 1990

*Gymnodinium peisonis* Schiller 1957

*Gymnodinium perplexum* van Meel 1969

*Gymnodinium placidum* Herdman 1922

*Gymnodinium polycomma* Larsen 1994

*Gymnodinium posthiemale* Schiller 1957

*Gymnodinium prolatum* Larsen 1994

*Gymnodinium pseudomirabile* Hansen & Flaim 2007

*Gymnodinium pulchrum* Schiller 1928

*Gymnodinium pumilum* Larsen 1994

*Gymnodinium punctatum* Pouchet 1887

*Gymnodinium puniceum* Kofoid & Swezy 1921

*Gymnodinium purpureum* Skuja 1956

*Gymnodinium pygmaeum* Lebour 1925

*Gymnodinium pyrocystis* Jörgensen 1912

*Gymnodinium radiatum* Kofoid & Swezy 1921

*Gymnodinium ravenescens* Kofoid & Swezy 1921

*Gymnodinium regulare* van Meel 1969

*Gymnodinium rhomboides* Schütt 1895

*Gymnodinium roseolum* (Schmarda) Stein 1878

*Gymnodinium roseostigma* Campbell 1973

*Gymnodinium rubricauda* Kofoid & Swezy 1921

*Gymnodinium rubrocinctum* Lebour 1925

*Gymnodinium schaefferi* Morris 1937

*Gymnodinium schuettii* Schiller 1957

*Gymnodinium scopulosum* Kofoid & Swezy 1921

*Gymnodinium semidivisum* Schiller 1928

*Gymnodinium situla* Kofoid & Swezy 1921

*Gymnodinium soyai* Hada 1970

*Gymnodinium sphaericum* (Calkins) Kofoid & Swezy 1921

*Gymnodinium sphaeroideum* Kofoid 1931

*Gymnodinium steini* (Klebs) Lindemann 1928

*Gymnodinium stellatum* Hulburt 1957

*Gymnodinium submontanum* Schiller 1957

*Gymnodinium subroseum* Campbell 1973

*Gymnodinium subrufescens* Martin 1929

*Gymnodinium sulcatum* Kofoid & Swezy 1921

*Gymnodinium terrum* Baumeister 1943

*Gymnodinium thomasi* Christen 1949

*Gymnodinium tintinnicola* Lohmann 1908

*Gymnodinium translucens* Kofoid & Swezy 1921

*Gymnodinium trapeziforme* Attaran-Fariman & Bolch 2007

*Gymnodinium triceratium* Skuja 1939

*Gymnodinium uberrimum* (Allman) Kofoid & Swezy 1921

*Gymnodinium uncatenum* (Hulburt) Hallegraeff 2002

*Gymnodinium valdecompressum* Campbell 1973

*Gymnodinium variabile* Herdman 1924

*Gymnodinium varians* Maskell 1877

*Gymnodinium venator* Flø Jørgensen & Murray 2004

*Gymnodinium verruculosum* Campbell 1973

*Gymnodinium vestifici* Schütt 1895

*Gymnodinium violescens* Kofoid & Swezy 1921

*Gymnodinium viridaliut* Schiller 1957

*Gymnodinium viridans* van Meel 1969

*Gymnodinium viridescens* Kofoid 1931

*Gymnodinium voukii* Schiller 1928

*Gymnodinium wawrikae* Schiller 1957

*Gymnodinium wilczeki* Pouchet 1894

*Gymnodinium wulffii* Schiller 1933

*Gymnodinium zachariasi* Lemmermann 1900
