# Supplementary material for: The Taxonomic Significance of Species That Have Only Been Observed Once: The Genus Gymnodinium (Dinoflagellata) as an Example
Source: PLoS One. 2012 Aug 30;7(8):e44015. doi: 10.1371/journal.pone.0044015 (PMC3431360; doi:10.1371/journal.pone.0044015)
Supplement: Appendix S6 — List of rejected Gymnodinium names. (DOCX) [file pone.0044015.s006.docx]

Appendix S6

*Gymnodinium acutiusculum* Okolodkov 1997

*Gymnodinium aestivale* Skvortzov 1968

*Gymnodinium bilobatum* van Meel 1969

*Gymnodinium cnodax* Conrad & Kufferath 1954

*Gymnodinium cyaneum* Schiller 1957

*Gymnodinium depressum* Skvortzov 1968

*Gymnodinium diamphidium* Norris 1961

*Gymnodinium frigidum* Woloszynska 1952

*Gymnodinium fukushimai* Hada 1966

*Gymnodinium galeiforme* Okolodkov 1997

*Gymnodinium luteo-viride* van Meel 1969

*Gymnodinium mammosum* van Meel 1969

*Gymnodinium massarti* (Conrad) Schiller 1933

*Gymnodinium maximum* Nordli 1951

*Gymnodinium nucaceum* Okolodkov 1997

*Gymnodinium obliquum* Okolodkov 1997

*Gymnodinium ovato-capitatum* van Meel 1969

*Gymnodinium ovoideum* Okolodkov 1997

*Gymnodinium pingue* van Meel 1969

*Gymnodinium planctonicum* Skvortzov 1968

*Gymnodinium rete* Schütt 1895

*Gymnodinium rotundatum* Skvortzov 1968

*Gymnodinium scaphium* van Meel 1969

*Gymnodinium servatum* Busch 1927

*Gymnodinium sinuatum* Skvortzov 1968

*Gymnodinium suffuscum* van Meel 1969

*Gymnodinium telma* van Meel 1969

*Gymnodinium triangularis* Lebour 1917

*Gymnodinium vas* van Meel 1969

*Gymnodinium vastum* Busch 1927

*Gymnodinium vernale* Skvortzov 1968
